# Design, Synthesis and Preliminary Evaluation of the Cytotoxicity and Antibacterial Activity of Novel Triphenylphosphonium Derivatives of Betulin

**DOI:** 10.3390/molecules27165156

**Published:** 2022-08-12

**Authors:** Mirosława Grymel, Anna Lalik, Alicja Kazek-Kęsik, Marietta Szewczyk, Patrycja Grabiec, Karol Erfurt

**Affiliations:** 1 Department of Organic Chemistry, Bioorganic Chemistry and Biotechnology, Silesian University of Technology, B. Krzywoustego 4, 44-100 Gliwice, Poland; 2 Department of Chemical Organic Technology and Petrochemistry, Silesian University of Technology, B. Krzywoustego 4, 44-100 Gliwice, Poland; 3 Biotechnology Center, Silesian University of Technology, B. Krzywoustego 8, 44-100 Gliwice, Poland; 4 Department of Systems Biology and Engineering, Silesian University of Technology, Akademicka 16, 44-100 Gliwice, Poland; 5 Department of Inorganic, Analytical Chemistry and Electrochemistry, Silesian University of Technology, B. Krzywoustego 6, 44-100 Gliwice, Poland

**Keywords:** betulin, triphenylphosphonium cation, anticancer, antibacterial activity

## Abstract

For several decades, natural products have been widely researched and their native scaffolds are the basis for the design and synthesis of new potential therapeutic agents. Betulin is an interesting biologically attractive natural parent molecule with a high safety profile and can easily undergo a variety of structural modifications. Herein, we describe the synthesis of new molecular hybrids of betulin via covalent linkage with an alkyltriphenylphosphonium moiety. The proposed strategy enables the preparation of semi-synthetic derivatives (28-TPP^⊕^ BN and 3,28-*bis*TPP^⊕^ BN) from betulin through simple transformations in high yields. The obtained results showed that the presence of a lipophilic cation improved the solubility of the tested analogs compared to betulin, and increased their cytotoxicity. Among the triphenylphosphonium derivatives tested, analogs **7a** (IC_50_ of 5.56 µM) and **7b** (IC_50_ of 5.77 µM) demonstrated the highest cytotoxicity against the colorectal carcinoma cell line (HCT 116). TPP^⊕^-conjugates with betulin showed antimicrobial properties against Gram-positive reference *Staphylococcus aureus ATCC 25923* and *Staphylococcus epidermidis ATCC 12228* bacteria, at a 200 µM concentration in water. Hence, the conjugation of betulin’s parent backbone with a triphenylphosphonium moiety promotes transport through the hydrophobic barriers of the mitochondrial membrane, making it a promising strategy to improve the bioavailability of natural substances.

## 1. Introduction

Advancements in medical science have allowed for the treatment of a wide range of diseases, however, many disorders lack necessary pharmaceuticals. The high systemic toxicity of medicinal preparations and increasing resistance of tumor cells to a significant number of drugs often limits anticancer therapy success. According to World Health Organization (WHO) reports, cancerous diseases are one of the biggest problems of modern medicine and are one of the main causes of death in the world in the 21st century [1]. Therefore, drug design is an important issue in modern medicinal chemistry. Despite many innovative tools that allow for the development of extremely advanced methods of treatment, many therapies are still based on active substances of natural origin. These substances act as basic structures that can be subjected to various chemical modifications in order to improve their physicochemical and pharmacokinetic properties.

**Figure 1:**
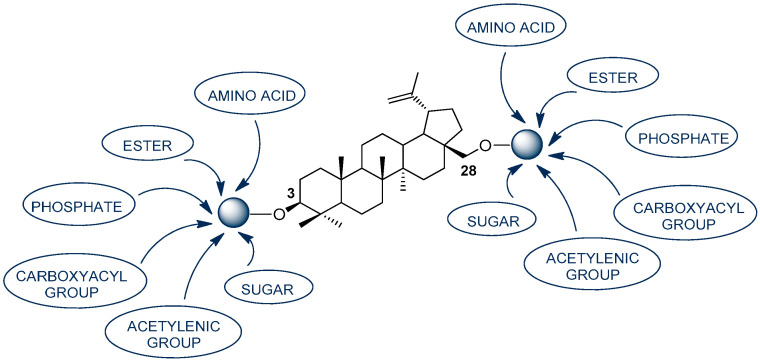
The selected modifications of the *BN* skeleton at positions C-3 and C-28 [3,4,5].

For several decades, natural products (*NPs*) have been widely researched in terms of searching for new drugs. It is *NPs* that are an invaluable source of native scaffolds, which are the basis for the design and development of new potential therapeutic agents. Naturally occurring pentacyclic lupane-type triterpenoids have attracted a lot of attention including betulin (*BN*, 3-lup-20(29)-ene-3,28-diol), which is one of the most available terpenoids in the plant kingdom. *BN* is a cheap, easily accessible natural active substance that can be readily extracted from the bark of several species of trees, especially white birch (*Betula pubescens*) [2,3]. Due to the presence of simply transformable functional groups in its skeleton (including C^3^-OH, C^28^-OH), *BN* has high synthetic potential for numerous semi-synthetic derivatives. *BN* is an interesting example of a biologically attractive natural parent molecule with a high safety profile and the possibility of making a variety of structural modifications (Figure 1) [3,4,5].

The multidirectional biological activity of natural *BN* has been confirmed by numerous research articles. Additionally, the reported derivatives of *BN* have shown a broad spectrum of bio-activity in terms of anticancer [4,6,7,8,9,10], antimalarial activity [11], antibacterial [12,13,14], antiviral [4,15,16,17], anti-inflammatory [18,19], or hepatoprotective properties [3]. Moreover, *BN* has a positive effect on the treatment of atopic dermatitis [20]. However, despite the abundance of *BN*, well-developed isolation methods from plant material as well as many studies confirming its very good biological properties, its use as a potential therapeutic agent is limited due to its low bioavailability, high hydrophobicity, and insufficient intracellular accumulation. The pentacyclic molecule and hydrophobic nature of the skeleton of *BN* hinders its ability to reach the target in vivo and obtain the desired therapeutic effect in acceptable therapeutic doses [21].

One of the most promising strategies for the design and synthesis of effective therapeutic agents is the conjugation of a native skeleton (e.g., *BN*) with triphenylphosphonium cation (TPP^⊕^) of low molecular weight, which promotes accumulation inside the cell’s mitochondria. Furthermore, the presence of the TPP^⊕^ group in a molecular hybrid improves the pharmacokinetic properties including solubility, bioavailability, and intermembrane transport as well as selectivity in targeting drugs for a specific purpose. The high lipophilicity and large ionic radius of TPP^⊕^ effectively reduce the activation energy required for membrane passage. The presence of a delocalized lipophilic cation can accelerate the transport of biologically active molecules across the mitochondrial membrane [22]. Studies have shown that, in contrast to other cellular organelles, mitochondria have a high negative transmembrane potential (Δψm). This potential is much higher for tumor cells, providing an opportunity for the selective delivery and accumulation of anticancer agents in mitochondria-targeted therapies [21,22,23,24].

**Figure 2:**
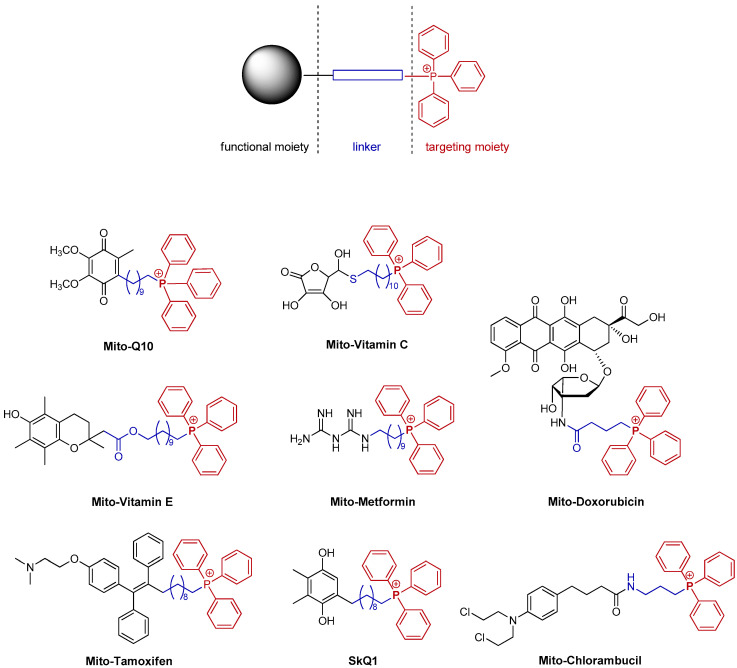
Examples of the TPP^⊕^-conjugated compounds [24,26,29,30,31,32,33,34,35].

Thus, the TPP^⊕^ moiety not only affects the physical properties, but also the mechanism of action of a potential drug. In addition, it increases the selectivity, which often reduces the drug dose, and in turn diminishes the harmful side effects [24,25]. Therefore, research into mitochondria-targeted anticancer drugs is an attractive prospect [24,25,26,27,28]. The strategy of modification of the native skeleton via conjugating with the TPP^⊕^ group has been used successfully for anticancer drugs such as doxorubicin [29], cisplatin [26], chlorambucil [30], metformin [31], and tamoxifen [32] because it facilitates their transport and selective accumulation in cancer cells (Figure 2).

Mitochondria play a vital role in a wide range of physiological and pathological processes. They are the main source of reactive oxygen species, and at the same time, are particularly susceptible to oxidative damage, contributing to the development of many diseases. Due to their functions, mitochondria may be an important molecular target for anticancer drugs as well as in the treatment of cardiovascular diseases or neurodegenerative diseases (e.g., Alzheimer’s disease or Parkinson’s disease) [36]. Non-targeted antioxidant therapeutics show low effectiveness, therefore, attempts have been made to modify them to increase the drug accumulation in the mitochondrial matrix. For example, Mito-Q10, (coenzyme Q10) has been described as a potential agent for the treatment of sepsis or Parkinson’s disease (Figure 2) [24,36].

**Figure 3:**
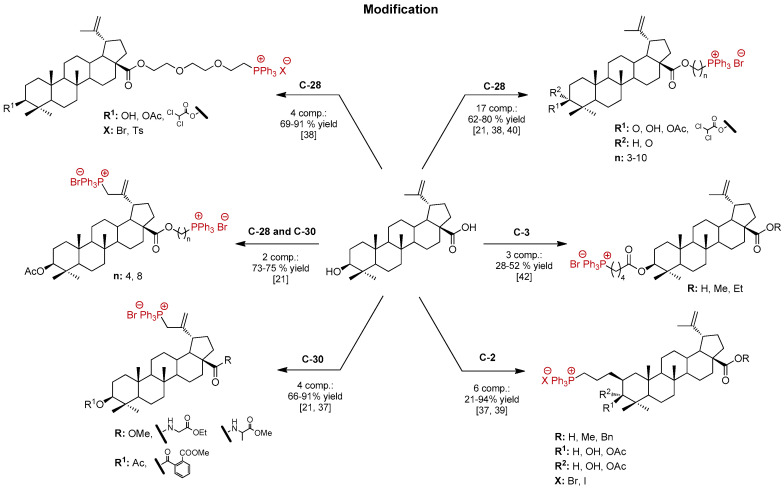
The chemical structures of the TPP^⊕^-conjugated with *BA*.

**Figure 4:**
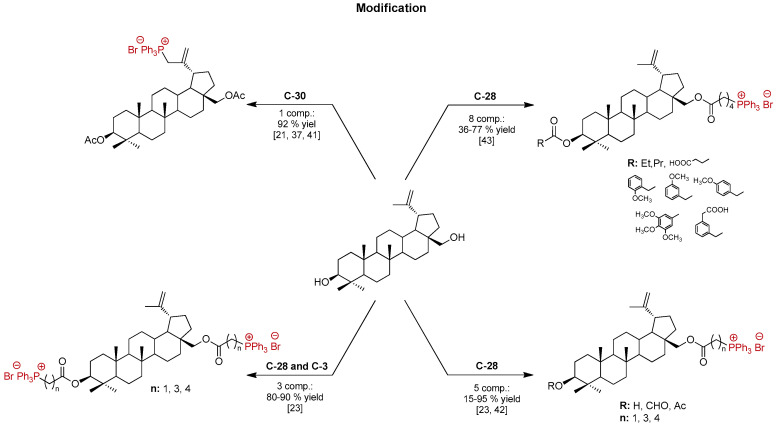
The chemical structures of the TPP^⊕^-conjugated with *BN*.

A relatively novel group of potential *mitocans* (acronym derived from the terms mitochondria and cancer) is conjugates of pentacyclic lupane-type triterpenoids including *BN* or betulinic acid (*BA*) with the lipophilic cation TPP^⊕^ [28]. Spivak et al. [21,28,37,38,39], Tsepaeva et al. [23,40,41], Ye et al. [42], and Xu et al. [43] reported the preparation of *BA* or *BN* conjugates, in which one or two TPP^⊕^ moieties were linked to the triterpenoid skeleton at positions C-2, C-3, C-28, or C-30 by the carbon–carbon or ester bonds, as shown in Figure 3 and Figure 4. The cytotoxic effect of these TPP^⊕^-analogs against various types of tumor cells toward *Schistosoma Mansoni* and antibacterial activity was analyzed [21,23,28,37,38,39,40,41,42,43,44].

In the library of known triphenylphosphonium derivatives of pentacyclic lupane-type triterpenoids, betulinic acid derivatives definitely dominate (TPP^⊕^-conjugated with *BA*, about 30 compounds, Figure 3). Both *BN* and *BA* are common in the plant kingdom, especially in the outer layer of the birch bark (*Betulaceae*, *Betula*, *Betula pendula*). However, *BN* is considerably more available (*BN* content was up to 34%, and *BA* was only 0.3% of dry weight [6]), which may be an advantage in terms of the economic analysis of methods in obtaining potential therapeutic agents.

**Scheme 1:**
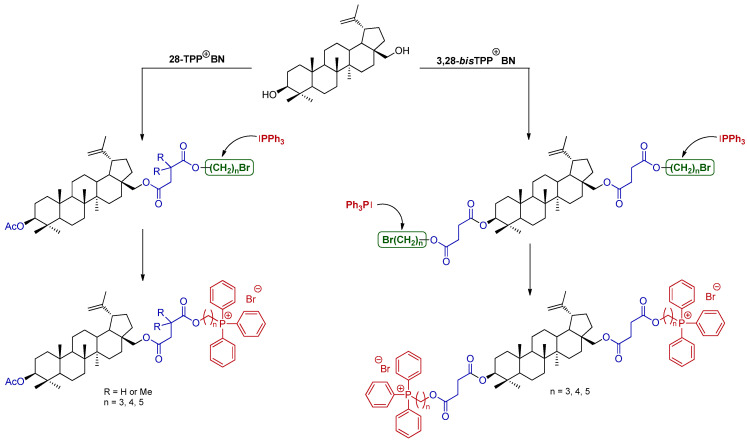
The synthetic route for the preparation of 28-TPP^⊕^ ΒΝ and 3,28-*bis*TPP^⊕^ ΒΝ.

Although *BN* derivatives have been extensively explored, to date, no structures have demonstrated the desired biological properties at a satisfactory dosage that would allow them to be used as drugs. The aim of the presented study was to evaluate the relationships between the biological effects and the structure of the new triphenylphosphonium derivatives of *BN*. Hence, we designed and synthesized molecular hybrids of *BN* by covalently linking a lipophilic alkyltriphenylphosphonium moiety (shown in dark red, Scheme 1) to the parent skeleton of *BN* through a linker (shown in blue, Scheme 1). In addition, in order to obtain a library of compounds intended for the initial assessment of biological activity (e.g., cytotoxicity, antibacterial activity), structural modifications of the *BN* skeleton were conducted via the introduction of one or two TPP^⊕^ moieties at the C-28 or C-3 and C-28 positions. We prepared a series of the *mono-* and *bis*TPP^⊕^ derivatives of *BN* through the multi-step synthesis (3 or 5 step) and their bio-activity was analyzed.

## 2. Results and Discussion

### 2.1. Synthesis of BN Analogs (***2**–**5***)

**Scheme 2:**
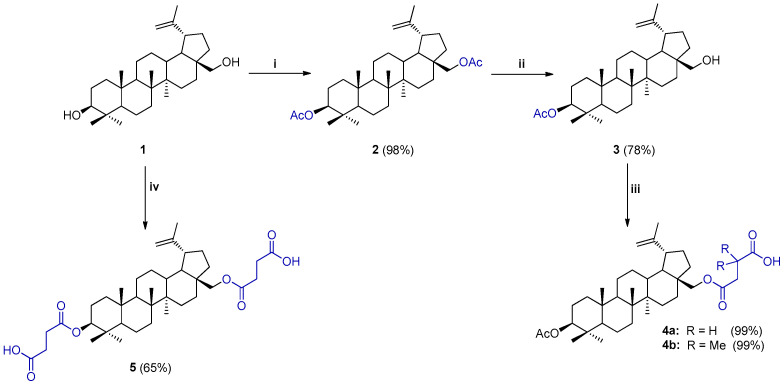
The synthesis of the *BN* analogs (**2**–**5**). Reagents and conditions: (**i**) Ac_2_O, DMAP, Py, r.t., 24 h; (**ii**) (*i*-PrO)_3_Al, *i*-PrOH, 80 °C, 2 h; (**iii**) SA or DMSA, DMAP, Py, reflux, 18–20 h; (**iv**) SA, Py, reflux, 9 h.

Starting materials 3,28-*O,O’-*diacetylbetulin **2** and 3-*O-*acetylbetulin **3** were synthesized according to the protocol described by Thibeault et al. (Scheme 2) [45]. In the first step, betulin was esterified with an excess of acetic anhydride in pyridine (Py) in the presence of 4-(dimethylamino)pyridine (DMAP) to give ester **2** in 98% yield. Then, the C-28 position of the *BN* backbone was selectively deprotected by treating crystalline ester **2** with aluminum isopropoxide ((*i*-PrO)_3_Al) in *i*-PrOH. After 2 h at 80 °C, followed by column chromatography purification, analog **3** was obtained in 78% yield.

The next step involved the introduction of a linker terminated with a carboxyl group (O(CO)CH_2_CR_2_COOH, R = H or Me) at position C-28 (*mono*-substituted *BN* analog **4**) or C-3 and C-28 (disubstituted *BN* analog **5**) of *BN*. The carboxyacyl moiety seemed to be an ideal linker component as it allowed for further structural modifications. In addition, numerous literature reports have suggested that the combination of the triterpene skeleton with this type of moiety improved the biological properties including anti-HIV, antibacterial, or antifungal activity [46,47]. In accordance with the published procedures [48,49] with some modifications, analog **3** was reacted with succinic anhydride (SA) or 2,2-dimethylsuccinic anhydride (DMSA) in dry pyridine in the presence of DMAP at reflux for 18–20 h. The synthesis of 3,28-*O*,*O*’-*bis*(3-carboxypropanoyl)betulin **5** was performed in a similar manner, but the reagents were used in a molar ratio of BN:SA (1:10) at reflux for 9 h. The crude products were purified by chloroform extraction and washed with the water and HCl solution. The *BN* analogs were obtained in excellent yields (**4a**: 99%, **4b**: 99%, **5**: 65%, Scheme 2).

### 2.2. Synthesis of 28-TPP^⊕^-Conjugates Derivatives of BN

**Scheme 3:**
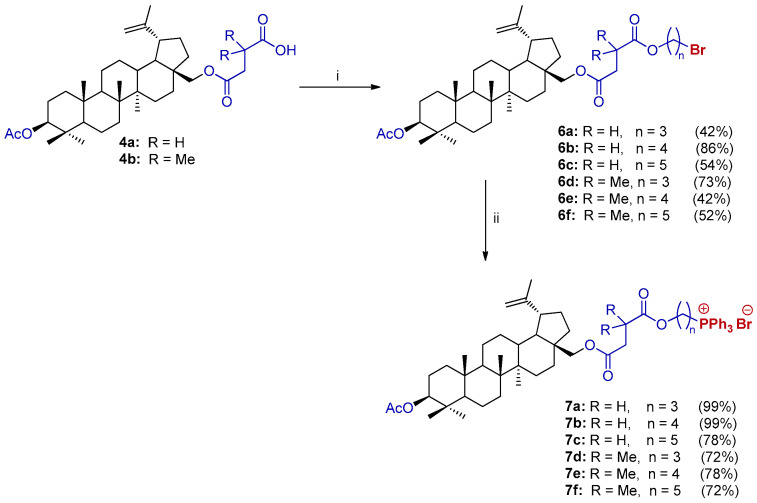
The synthesis of the 28-TPP^⊕^ BN derivative **7**. Reagents and conditions: (**i**) Br(CH_2_)_n_Br, DMF/MeCN (10/1, *v*/*v*), K_2_CO_3_, 50 °C, 18–20 h; (**ii**) Ph_3_P, argon, 120 °C, 6–12 h.

The synthesis method of new molecular *BN* hybrids (28-TPP^⊕^ BN) with one TPP moiety attached via the linker C^28^-O(CO)CH_2_CR_2_COO(CH_2_)_n_ developed by our group consists of a few steps, as shown in Scheme 3. The desired analogs were synthesized via alkylation of the carboxyl group of (carboxyacyl)betulin **4** with dibromoalkanes in a molar ratio of **4**:Br(CH_2_)_n_Br (1:3) in a DMF/MeCN system in the presence of K_2_CO_3_ at 50 °C for 18–20 h. 1,3-Dibromopropane, 1,4-dibromobutane, and 1,5-dibromopentane were employed to examine the influence of the chain length on bio-activity. 3-*O*-Acetyl-28-O’-(3’-(bromoalkoxycarbonyl)propanoyl)betulin (**6a**–**6c**) and 3-*O*-acetyl-28-*O*’-(3’,3’-dimethyl-3’-(bromoalkoxycarbonyl)propanoyl)betulin (**6d**–**6f**) were isolated by extraction with ethyl acetate and subsequent purification by column chromatography to produce the products in satisfactory yields (42–86%).

The final step of the 28-TPP^⊕^ BN synthesis was the substitution of the bromide anion of analog **6** with the TPP group by heating a homogenous mixture of 28-(bromoalkoxycarbonyl)propanoyl)betulin **6** and triphenylphosphine without a solvent under an Ar atmosphere. Optimization of the procedure consisted of examining the proportions of reagents, the temperature and reaction time, where at a molar ratio of analog **6**/triphenylphosphine (1:2) at 120 °C for 6–12 h, the highest yields were obtained. Additionally, column chromatography was not necessary for all analogs, but all required extraction with diethyl ether and then crystallization from the diethyl ether/ethyl acetate (1:4, *v*/*v*), resulting in a high yield (72–99%, Scheme 3).

### 2.3. Synthesis of 3,28-bisTPP^⊕^-Conjugates Derivatives of BN

**Scheme 4:**
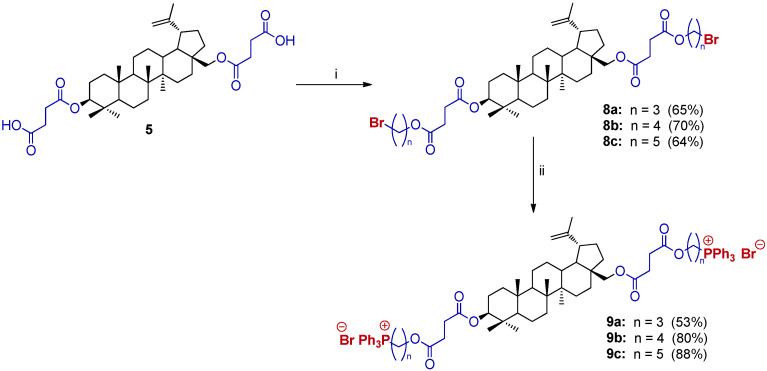
The synthesis of 3,28-*bis*TPP^⊕^ BN **9**. Reagents and conditions: (**i**) Br(CH_2_)_n_Br, DMF/MeCN (10/1, *v*/*v*), K_2_CO_3_, 50 °C, 18–20 h; (**ii**) triphenylphosphine, Ar, 120 °C, 12–24 h.

We also investigated the influence of two TPP^⊕^ groups in the molecular hybrids of *BN* toward their pharmacokinetic properties. The synthetic route of 3,28-*bis*TPP^⊕^ BN is depicted in Scheme 4.

In the first step, 3,28-*O*,*O*’-*bis*(3-carboxypropanoyl)betulin **5** was reacted with 1,3-dibromopropane, 1,4-dibromobutane or 1,5-dibromopentane at a molar ratio of **5**/Br(CH_2_)_n_Br (1:6) in a DMF/MeCN system with K_2_CO_3_ at 50 °C for 20 h. The analog **8** was obtained in satisfactory yields (64–70%) according the procedure described above. Then, the homogenous mixture (**8** and triphenylphosphine) was heated at 120 °C without solvent under an Ar atmosphere. The final product **9** was isolated by extraction (diethyl ether, and diethyl ether/ethyl acetate) at elevated temperature in good yields (80–88%, Scheme 4). Only analog **9a** required column chromatography (53% yield).

The structures of all of the synthesized compounds (**2**–**9**) were confirmed by spectroscopic methods (^1^H, ^13^C, ^31^P NMR, FTIR, and HRMS, Supplementary Materials). The ^31^P NMR spectra of analogs **7** and **9** showed signals confirming the presence of TPP^⊕^ in the range of 19.4–24.8 ppm. A characteristic feature in the ^13^C NMR spectra of the organophosphorus compounds was the splitting of specific signals into doublets caused by coupling the phosphorus atom with selected carbon atoms. Chemical shifts and *J*_C-P_ coupling constants of great diagnostic value observed for the TPP^⊕^ group are summarized in Table 1.

### 2.4. Cytotoxicity Studies 

**Figure 5:**
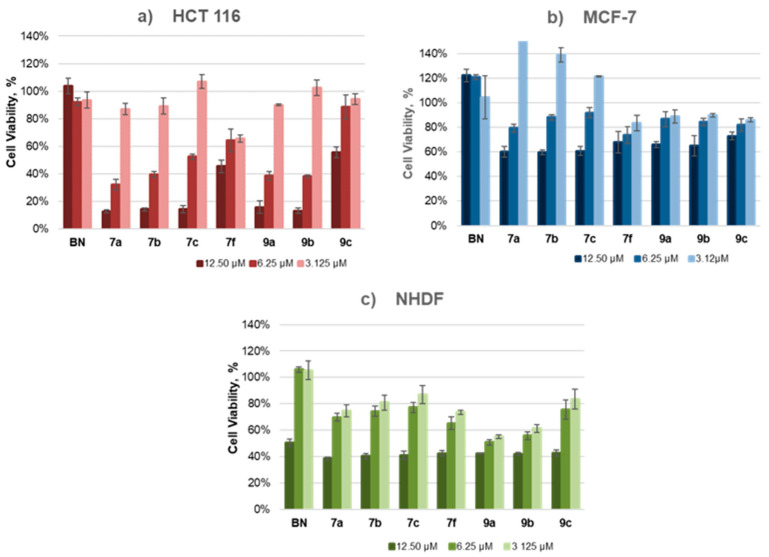
The dependence of cell viability on the concentration of *BN* and 28-TPP^⊕^ BN (**7**) and the 3,28-*bis*TPP^⊕^ BN analog (**9**) after 24 h of incubation.

The obtained new molecular hybrids of *BN* were screened in order to initially investigate their cytotoxicity as well as examine the relationships between the structure and biological effect. The common element of all of the tested compounds was the presence of a lipophilic moiety (CH_2_)_n_TPP^⊕^ (n = 3, 4, 5). The research was conducted on two groups of compounds: 28-TPP^⊕^ BN and 3,28-*bis*TPP^⊕^ BN. Their cytotoxicity was investigated on two cancer cell lines: HCT 116 (colorectal carcinoma cell line) and MCF-7 (human breast adenocarcinoma cell line). The proliferation of tumor cells treated with the tested compounds (**7** and **9**) at 12.5–3.125 µM concentrations were determined after 24 h of incubation. Additionally, all compounds were tested against the NHDF cell line (Normal Human Dermal Fibroblast cells) to assess their safety. The effect of these compounds was compared with that of *BN* doses, as shown in Figure 5. Half-maximal inhibitory concentrations (IC_50_) of triphenylphosphonium analogs of *BN* were determined using a CCK-8 assay and are summarized in Table 2.

As expected, triphenylphosphonium derivatives of *BN* showed greater cytotoxicity than the parent *BN* toward all of the cell lines tested. The level of inhibition of cell viability depended on the concentration of the tested substances and cell type. The tested analogs of BN had the greatest effect on the viability of the HCT 116 cells (Figure 5a) and the lowest on the viability of the MCF-7 cells (Figure 5b).

When comparing the biological effect of the *mono*-TPP^⊕^ BN derivatives (**7a**–**7c**, linker without an additional Me group), it seemed that the length of the linker did not influence their activity in the in vitro tests. The 28-TPP^⊕^ BN conjugates (**7a**–**7c**) with a variable length, and an alkyl linker (n = 3, 4, 5) similarly inhibited the viability in both tumor cells (HCT 116: IC_50_ = 5.56–6.48, MCF-7: IC_50_ = 13.71–15.52). The exception was analog **9c**, with two TPP^⊕^ cations and a pentyl chain, which, compared to compounds with a shorter linker (propyl or butyl chain), showed lower cytotoxicity against the HCT 116 cells (IC_50_: **9a** < **9b** < **9c**; Table 2). Importantly, compounds **7a**–**7c** were almost twice less toxic toward the healthy cells (NHDF), with IC_50_ values ranging from 9.68 to 11.29 μM, which demonstrated their selectivity.

Unfortunately, in the course of further studies, it was revealed that the introduction of an additional Me group into the linker **7f** reduced this bio-activity compared to compound **7c** against the HCT 116 and MCF-7 tumor cells whereas no significant effect of the Me groups attached to the linker was observed on the bio-activity of compound **7f** in normal NHDF cells.

We observed that the presence of both one and two lipophilic cations improved the solubility of the tested analogs compared to *BN*, which slightly increased their cytotoxicity, especially against the colorectal carcinoma cell line (HCT 116). Among the triphenylphosphonium derivatives of *BN* tested, analossg **7a** (IC_50_ of 5.56 µM) and **7b** (IC_50_ of 5.77 µM) demonstrated the highest cytotoxicity against this cell line at low micromolar concentrations. This supported the hypothesis that the conjugation of the *BN* native backbone with the TPP^⊕^ moiety allowed for its transport through the hydrophobic barriers of the mitochondrial membrane, making it a promising strategy to improve the bioavailability of natural substances.

### 2.5. Antibacterial Studies

Investigations were carried out using different concentrations of solutions from 25 to 250 µM. A 25 µM concentration of the tested derivatives was not enough to inhibit the growth of both Gram-positive *S. aureus ATCC 25923* bacteria. When the concentration of analogs **7d**–**7f** and **9a**–**9c** increased to 200 µM, the growth of *S. aureus ATCC 25923* and *S. epidermidis ATCC 12228* was inhibited. Furthermore, the optical density of all compounds remained unchanged after 18 h of bacteria culture. In the case of analogs **7a**–**7c**, the optical density values were between 0.9 and 2.2 when they were cultured with *S. aureus ATCC 25923*. The bacterial growth was slower compared to the control sample (TSB-culture medium). However, the studied TPP^⊕^-BN derivatives did not inhibit the growth of *S. epidermidis ATCC 12228* bacteria. However, at a 250 µM concentration, analogs **7a** and **7b** greatly inhibited the growth of *S. aureus ATCC 25923* and *S. epidermidis ATCC 12228*. In the case of analog **7c**, the optical density increased up to 0.7 after 18 h of the sample culture with both kinds of Gram-positive bacteria. In contrast, the reference sample’s (TSB) optical density increased to 7.5 for *S. aureus ATCC 25923*, and 4.9 for *S. epidermidis ATCC 12228* after 18 h of bacteria culture. All of the investigated compounds did not inhibit the growth of Gram-negative *Escherichia coli ATCC 25922* bacteria. All of the analog values of the measured optical density were similar to that of the reference sample (5.0–5.1, Table 3).

The obtained results showed that 28-TPP^⊕^ BN (**7d**–**7f**) and 3,28-*bis*TPP^⊕^ BN (**9a**–**9c**) could be employed as agents for the inhibition of Gram-positive bacteria (*S. aureus ATCC 25923* and *S. epidermidis ATCC 12228*) growth at a concentration of 200 µM in an aqueous solution.

## 3. Materials and Methods

### 3.1. General Information

NMR spectra (^1^H and ^13^C) were recorded on a Varian spectrometer at operating frequencies of 600 or 400 MHz and 150 or 100 MHz, respectively, using TMS as the resonance shift standard. CDCl_3_ was used as the solvent, which was purchased from ACROS Organics (Geel, Belgium). The ^31^P NMR spectra were acquired using a Varian 400 spectrometer at 161.9 MHz, where the resonance shift of H_3_PO_4_ was determined as 0 ppm. All chemical shifts (δ) were reported in ppm and coupling constants (*J*) in Hz. The following abbreviations were used to explain the observed multiplicities: s—singlet; d—doublet; dd—double doublet; ddd—doublet of double doublet; t—triplet, dd~t—overlapping double doublet that resembles a triplet (with similar values of coupling constants); m—multiplet; br—broad. IR-spectra were measured on a Nicolet 6700 FT-IR spectrophotometer, Thermo Scientific (Waltham, MA, USA) (attenuated total reflectance method; ATR). High resolution mass spectrometry analyses were performed using a Waters Xevo G2 Q-TOF mass spectrometer (Waters Corporation, Milford, MA, USA) equipped with an ESI source operating in positive-ion mode. The accurate mass and composition for the molecular ion adducts were calculated using MassLynx software (Waters) incorporated in the instrument.

Reactions were monitored by TLC analysis on precoated plates of silica gel 60 F_254_ (Merck Millipore, Burlington, MA, USA). The TLC plates were inspected under UV light (λ = 254 nm) or charring after spraying with 10% solution of sulfuric acid in ethanol. Crude products were purified using column chromatography performed on silica gel 60 (70–230 mesh, Fluka).

3,28-*O,O’*-Diacetylbetulin **2**; 3-*O*-acetylbetulin **3** [45], 3-*O*-acetyl-28-*O*’-(3’-carboxypropanoyl)betulin **4a**; 3-*O*-acetyl-28-*O*’-(3’,3’-dimethyl-3’-carboxypropanoyl)betulin **4b [48]**, and 3,28-*O,O*′-bis(3′-carboxypropanoyl)betulin **5 [49]** were prepared according to the respective published procedures.

All chemicals used in the study were purchased from Sigma-Aldrich (St. Louis, MO, USA), Fluka, Avantor (Radnor Township, PA, USA) and ACROS Organics, and used without further purification.

### 3.2. Chemistry

#### 3.2.1. General Procedure for the Synthesis of 3-O-Acetyl-28-O’-(carboxyacyl)betulin (**4**)

3-*O*-Acetylbetulin (**3**, 2.50 mmol, 1.21 g, 1 eq.), SA (7.50 mmol, 0.75 g, 3 eq.) or DMSA (7.50 mmol, 0.96 g, 3 eq.) and DMAP (7.50 mmol, 0.92 g, 3 eq.) were dissolved in dry pyridine (19 mL). The reaction mixture was stirred under reflux for 18–20 h. After cooling to rt, 10% hydrochloric acid solution (20 mL) and water (35 mL) were added. The product was extracted with CHCl_3_ (4 × 70 mL). The combined organic extracts were washed with water (70 mL), 5% hydrochloric acid solution (140 mL), brine (70 mL), water (70 mL), dried over MgSO_4_, and filtered. The solvent was evaporated under reduced pressure producing analog **4a**, which was used in the next step without further purification. Analog **4b** was purified using column chromatography (DCM/MeOH, gradient: 100:1 to 50:1).

*3-O-Acetyl-28-O’-(3’-carboxypropanoyl)betulin (**4a**)* was obtained as a resin (1.45 g, 99% yield); *R_f_ =* 0.78 (DCM/MeOH, 10:1, *v*/*v*). ^1^H NMR (600 MHz, CDCl_3_): δ_H_ 0.75, 0.76, 0.88, 0.94, 1.31 (all s, 3H each, H-23–H-27), 1.60 (s, 3H *J_1_* = 5.9 Hz, H-30), 0.62–2.06 (m, 24H, CH, CH_2_ *BN* scaffold), 1.96 (s, 3H, CH_3_CO), 2.34 (td, 1H, *J_2_* = 11.0 Hz, H-19), 2.54–2.63 (m, 4H, O(CO)CH_2_CH_2_), 3.80 (d, 1H, *J* = 10.8 Hz, H-28b), 4.22 (d, 1H, *J* = 13.2 Hz, H-28a), 4.38 (dd, 1H, *J_1_* = 5.4 Hz, *J_2_* = 10.8 Hz, H-3), 4.50 (s, 1H, H-29b), 4.60 (s, 1H, H-29a) ppm; ^13^C NMR (150 MHz, CDCl_3_): δ_C_ 14.7, 16.0, 16.2, 16.5, 18.2, 19.1, 20.8, 21.3, 23.7, 25.1, 27.0, 27.9, 28.8, 29.1, 29.5, 29.7, 34.1, 34.5, 37.1, 37.6, 37.8, 38.4, 40.9, 42.7, 46.4, 47.7, 48.8, 50.3, 55.4, 63.2, 80.9, 109.9, 150.1, 171.1, 172.4, 176.9 ppm; IR (ATR) ν: 2938, 1729, 1714, 1244, 1158 cm^−1^.

*3-O-Acetyl-28-O’-(3’,3’-dimethyl-3’-carboxypropanoyl)betulin (**4b**)* was obtained as a resin (1.52 g, 99% yield); *R_f_* = 0.64 (DCM/MeOH, 10:1, *v*/*v*). ^1^H NMR (600 MHz, CDCl_3_): δ_H_ 0.83, 0.84, 0.96, 1.02, 1.31 (all s, 3H each, H-23–H-27), 1.39 (s, 6H, CMe_2_), 1.68 (s, 3H, H-30), 0.70–2.00 (m, 24H, CH, CH_2_ *BN* scaffold), 2.04 (s, 3H, CH_3_CO), 2.41 (td, 1H, *J_1_* = 5.8 Hz, *J_2_* = 11.1 Hz, H-19), 2.64 (s, 2H, O(CO)CH_2_), 3.87 (d, 1H, *J* = 10.9 Hz, H-28b), 4.26 (d, 1H, *J* = 9.2 Hz, H-28a), 4.47 (dd, 1H, *J_1_* = 5.6 Hz, *J_2_* = 10.7 Hz, H-3), 4.58 (s, 1H, H-29b), 4.68 (s, 1H, H-29a) ppm; ^13^C NMR (150 MHz, CDCl_3_): δ_C_ 14.7, 16.0, 16.1, 16.4, 18.1, 19.1, 20.7, 21.2, 23.6, 25.1, 25.2, 25.3, 26.9, 27.9, 29.5, 29.7, 34.0, 34.5, 37.0, 37.5, 37.7, 38.3, 40.4, 40.8, 42.6, 44.3, 46.2, 47.6, 48.7, 50.2, 55.3, 63.0, 81.9, 109.8, 150.0, 171.06, 171.4, 183.0 ppm; IR (ATR) ν: 2940, 1728, 1703, 1242, 1193 cm^−1^.

#### 3.2.2. Synthesis of 3,28-O,O′-Bis(3′-carboxypropanoyl)betulin (**5**)

*BN* (2.50 mmol, 1.11 g, 1 eq.) and SA (25.00 mmol, 2.50 g, 10 eq.) were dissolved in dry pyridine (26 mL) and stirred under reflux for 9 h. After cooling to r.t., 10% hydrochloric acid solution (26 mL) and water (48 mL) were added. The product was extracted with CHCl_3_ (6 × 120 mL). The combined organic layers were concentrated to a 200 mL volume, washed with water (200 mL), 5% hydrochloric acid solution (2 × 90 mL), brine (200 mL), H_2_O (200 mL), dried over MgSO_4_, and filtered. Then, the solvent was evaporated under reduced pressure.

*3,28-O,O′-Bis(3′-carboxypropanoyl)betulin (**5**)* was obtained as a resin (1.04 g, 65% yield); *R_f_ =* 0.23 (DCM:MeOH, 100:1). ^1^H NMR (600 MHz, CDCl_3_): δ_H_ 0.83, 0.84, 0.85, 0.98, 1.03 (all s, 3H each, H-23–H-27), 1.69 (s, 3H, H-30), 0.70–2.00 (m, 24H, CH, CH_2_ *BN* scaffold), 2.43 (td, 1H, *J_1_* = 5.8 Hz, *J_2_* = 11.1 Hz, H-19), 2.60–2.70 (m, 8H, 2 × O(CO)CH_2_CH_2_), 3.88 (d, 1H, *J* = 10.9 Hz, H-28b), 4.31 (d, 1H, *J* = 11.0 Hz, H-28a), 4.50 (dd, 1H, *J_1_* = 5.6 Hz, *J_2_* = 10.8 Hz, H-3), 4.59 (dd, 1H, *J_1_* = 1.4 Hz, *J_2_* 2.3 Hz, H-29b), 4.68 (d, 1H, *J* = 2.0 Hz, H-29a) ppm; ^13^C NMR (150 MHz, CDCl_3_): δ_C_ 14.8, 16.0, 16.1, 16.5, 18.2, 19.1, 20.8, 23.6, 25.2, 27.0, 27.9, 29.0, 29.1, 29.3, 29.6, 29.7, 34.1, 34.4, 37.1, 37.6, 37.8, 38.3, 40.9, 42.7, 46.5, 47.7, 48.8, 50.3, 55.4, 63.2, 81.6, 109.9, 150.1, 171.7, 172.3, 177.9, 178.0 ppm; IR (ATR) ν: 2944, 1709, 1160, cm^−1^.

#### 3.2.3. General Procedure for the Synthesis of Bromides of BN (**6**)

To a solution of *BN* derivative (**4,** 0.25 mmol, 1 eq.) and K_2_CO_3_ (0.25 mmol, 34.6 mg, 1 eq.) in DMF (1 mL/100 mg **4**) and MeCN (0.1 mL/100 mg **4**), the appropriate dibromoalkane (Br(CH_2_)_n_Br, n = 3, 4, 5; 0.75 mmol, 3 eq.) was added. The reaction was carried out at 50 °C for 18–21 h. After the reaction was completed, the obtained mixture was diluted with cold water and extracted with ethyl acetate (5 × 19 mL). The combined organic layers were washed with brine (2 × 65 mL), dried over MgSO_4_, and the solvent was evaporated under reduced pressure. Then, crude product **6** was washed with methanol (2 × 0.5 mL) and purified using column chromatography (DCM/MeOH, gradient: 100:1 to 50:1).

*3-O-Acetyl-28-O’-(3’-(3"-bromopropyloxycarbonyl)propanoyl)betulin (**6a**)* was obtained as a resin (74.1 mg, 42% yield); R*_f_* = 0.18 (DCM:MeOH, 100:1). HRMS (ESI^+^) *m*/*z*: calcd for C_39_H_61_BrO_6_Na ([M+Na]^+^) 727.3549; found 727.3546; ^1^H NMR (600 MHz, CDCl_3_): δ_H_ 0.83, 0.84, 0.85, 0.97, 1.03 (all s, 3H each, H-23–H-27), 1.68 (s, 3H, H-30), 0.70–2.00 (m, 24H, CH, CH_2_ *BN* scaffold), 2.04 (s, 3H, CH_3_CO), 2.16–2.20 (m, 2H, CH_2_ fragment of linker), 2.43 (td, 1H, *J_1_* = 5.8 Hz, *J_2_* = 11.1 Hz, H-19), 2.64–2.66 (m, 4H, O(CO)CH_2_CH_2_), 3.46 (t, 2H, *J* = 6.5 Hz, CH_2_Br), 3.87 (d, 1H, *J* = 11.1 Hz, H-28b), 4.24 (t, 2H, *J* = 6.1 Hz, (CO)OCH_2_), 4.29 (dd, 1H, *J_1_* = 1.9 Hz, *J_2_* = 11.1 Hz, H-28a), 4.46 (dd, 1H, *J_1_* = 5.9 Hz, *J_2_* = 10.5 Hz, H-3), 4.59 (s, br, 1H, H-29b), 4.68 (s, br, 1H, H-29a) ppm; ^13^C NMR (150 MHz, CDCl_3_): δ_C_ 14.7, 16.0, 16.1, 16.4, 18.1, 19.1, 21.3, 20.7, 23.6, 25.1, 27.0, 27.9, 29.1, 29.2, 29.3, 29.5, 29.7, 31.6, 34.1, 34.5, 37.0, 37.5, 37.7, 38.3, 40.8, 42.6, 46.4, 47.7, 48.7, 50.2, 55.3, 62.4, 63.1, 80.8, 109.9, 150.0, 170.9, 172.1, 172.5 ppm; IR (ATR) ν: 2943, 1731, 1244, 1155, 732 cm^−1^.

*3-O-Acetyl-28-O’-(3’-(4"-bromobutyloxycarbonyl)propanoyl)betulin (**6b**)* was obtained as a resin (154.8 mg, 86% yield); R*_f_* = 0.28 (DCM:MeOH, 100:1). HRMS (ESI^+^) *m*/*z*: calcd for C_40_H_64_BrO_6_ ([M+H]^+^) 719.3886, found 719.3885; ^1^H NMR (400 MHz, CDCl_3_): δ_H_ 0.83, 0.84, 0.85, 0.97, 1.03 (all s, 3H each, H-23–H-27), 1.68 (s, 3H, H-30), 0.75–2.00 (m, 28H, CH, CH_2_ *BN* scaffold and (CH_2_)_2_ fragment of linker), 2.04 (s, 3H, CH_3_CO), 2.43 (td, 1H, *J_1_* = 5.8 Hz, *J_2_* = 11.1 Hz, H-19), 2.61–2.68 (m, 4H, O(CO)CH_2_CH_2_), 3.44 (t, 2H, *J* = 6.0 Hz, CH_2_Br), 3.88 (d, 1H, *J* = 11.1 Hz, H-28b), 4.13 (t, 2H, *J* = 6.0 Hz, (CO)OCH_2_), 4.29 (d, 1H, *J* = 11.1 Hz, H-28a), 4.45–4.49 (m, 1H, H-3), 4.59 (s, br, 1H, H-29b), 4.68 (s, br, H-29a) ppm; ^13^C NMR (100 MHz, CDCl_3_): δ_C_ 14.7, 16.0, 16.1, 16.4, 18.1, 19.1, 20.7, 21.3, 23.6, 25.1, 27.0, 27.2, 27.9, 29.1, 29.2, 29.3, 29.5, 29.7, 33.0, 34.1, 34.5, 37.0, 37.5, 37.7, 38.3, 40.8, 42.6, 46.4, 47.7, 48.7, 50.2, 55.3, 63.0, 63.7, 80.9, 109.8, 150.0, 170.9, 172.2, 172.5 ppm; IR (ATR) ν: 2946, 1732, 1246, 1156, 734 cm^−1^.

*3-O-Acetyl-28-O’-(3’-(5"-bromopentyloxycarbonyl)propanoyl)betulin (**6c**)* was obtained as a resin (99.1 mg, 54% yield); R*_f_* = 0.31 (DCM:MeOH, 100:1). HRMS (ESI^+^) *m*/*z*: calcd for C_41_H_65_BrO_6_Na ([M+Na]^+^) 755.3862, found 755.3870; ^1^H NMR (600 MHz, CDCl_3_): δ_H_ 0.83, 0.84, 0.85, 0.97, 1.03 (all s, 3H each, H-23–H-27), 1.68 (s, 3H, H-30), 0.70–2.00 (m, 30H, CH, CH_2_ *BN* scaffold and (CH_2_)_3_ fragment of linker), 2.04 (s, 3H, CH_3_CO), 2.43 (td, 1H, *J_1_* 5.8 Hz, *J_2_* = 11.1 Hz, H-19), 2.62–2.66 (m, 4H, O(CO)CH_2_CH_2_), 3.42 (t, 2H, *J* = 6.1 Hz, CH_2_Br), 3.87 (d, 1H, *J* = 10.8 Hz, H-28b), 4.10 (t, 2H, *J* = 6.6 Hz, (CO)OCH_2_), 4.29 (d, 1H, 11.1 Hz, H-28a), 4.46 (dd, 1H, *J_1_* = 5.6 Hz, *J_2_* = 10.8 Hz, H-3), 4.59 (s, br, 1H, H-29b), 4.68 (d, 1H, *J* 2.3 Hz, H-29a) ppm; ^13^C NMR (150 MHz, CDCl_3_): δ_C_ 14.7, 16.0, 16.1, 16.4, 18.1, 19.1, 20.7, 21.2, 23.6, 24.5, 25.1, 27.0, 27.7, 27.9, 29.1, 29.2, 29.5, 29.7, 32.2, 33.3, 34.1, 34.5, 37.0, 37.5, 37.7, 38.3, 40.8, 42.6, 46.4, 47.7, 48.7, 50.2, 55.3, 63.0, 64.3, 80.8, 109.8, 150.0, 170.9, 172.2, 172.5 ppm; IR (ATR) ν: 2942, 1730, 1244, 1155, 731 cm^−1^.

*3-O-Acetyl-28-O’-(3’,3’-dimethyl-3’-(3"-bromopropyloxycarbonyl)propanoyl)betulin (**6d**)* was obtained as a resin (133.9 mg, 73% yield); R*_f_* = 0.28 (DCM:MeOH, 100:1); HRMS (ESI^+^) *m*/*z*: calcd for C_41_H_66_BrO_6_ ([M+H]^+^) 733.4043, found 733.4045; ^1^H NMR (400 MHz, CDCl_3_): δ_H_ 0.76, 0.77, 0.89, 0.95, 1.32 (all s, 3H each, H-23–H-27), 1.21 (s, 6H, CMe_2_), 1.61 (s, 3H, H-30), 0.65–1.93 (m, 24H, CH, CH_2_ *BN* scaffold), 1.97 (s, 3H, CH_3_CO), 2.12 (d, 2H, *J* = 6.3 Hz, CH_2_ fragment of linker), 2.35 (td, 1H, *J_1_* = 5.7 Hz, *J_2_* = 11.1 Hz, H-19), 2.56 (s, 2H, O(CO)CH_2_), 3.39 (t, 2H, *J* = 6.6 Hz, CH_2_Br), 3.78 (d, 1H, *J* = 11.0 Hz, H-28b), 4.14–4.21 (m, 3H, H-28a and (CO)OCH_2_), 4.40 (dd, 1H, *J_1_* = 5.6 Hz, *J_2_* 10.8 Hz, H-3), 4.52 (s, br, 1H, H-29b), 4.61 (s, br, 1H, H-29a) ppm; ^13^C NMR (100 MHz, CDCl_3_): δ_C_ 14.7, 16.0, 16.1, 16.5, 18.1, 19.1, 20.8, 21.3, 23.7, 25.1, 25.5, 25.6, 27.0, 27.9, 29.5, 29.7, 31.7, 34.1, 34.5, 37.0, 37.6, 37.8, 38.4, 40.6, 40.9, 42.7, 44.5, 46.3, 47.7, 48.8, 50.3, 55.4, 62.5, 62.9, 80.9, 109.9, 150.0, 171.0, 172.0, 176.4 ppm; IR (ATR) ν: 2931, 1732, 1245, 1177 cm^−1^.

*3-O-Acetyl-28-O’-(3’,3’-dimethyl-3’-(4"-bromobutyloxycarbonyl)propanoyl)betulin (**6e**)* was obtained as a resin (78.5 mg, 42% yield); *R_f_ =* 0.29 (DCM:MeOH, 100:1); HRMS (ESI^+^) *m*/*z*: calcd for C_42_H_68_BrO_6_ ([M+H]^+^) 747.4199, found 747.4194; ^1^H NMR (400 MHz, CDCl_3_): δ_H_ 0.77, 0.78, 0.89, 0.95, 1.32 (all s, 3H each, H-23–H-27), 1.20 (s, 6H, CMe_2_), 1.61 (s, 3H, H-30), 0.70–1.93 (m, 28H, CH, CH_2_ *BN* scaffold and (CH_2_)_2_ fragment of linker), 1.97 (s, 3H, CH_3_CO), 2.35 (td, 1H, *J_1_* = 5.7 Hz, *J_2_* = 10.9 Hz, H-19), 2.56 (s, 2H, O(CO)CH_2_), 3.37 (t, 2H, *J* = 6.6 Hz, CH_2_Br), 3.78 (d, 1H, *J* = 11.0 Hz, H-28b), 4.05 (t, 2H, *J* = 6.3 Hz, (CO)OCH_2_), 4.17 (d, 1H, *J* = 11.1 Hz H-28a), 4.39–4.42 (m, 1H, H-3), 4.52 (s, br, 1H, H-29b), 4.61 (s, br, H-29a) ppm; ^13^C NMR (100 MHz, CDCl_3_): δ_C_ 14.7, 16.0, 16.1, 16.5, 18.1, 19.1, 20.8, 21.3, 23.7, 25.1, 25.46, 25.48, 27.0, 27.2, 27.9, 29.3, 29.5, 29.7, 33.1, 34.1, 34.5, 37.0, 37.6, 37.8, 38.4, 40.9, 40.6, 42.7, 44.5, 46.3, 47.7, 48.8, 50.3, 55.4, 62.8, 63.7, 80.9, 109.9, 150.0, 171.0, 172.0, 176.6 ppm; IR (ATR) ν: 2942, 1731, 1246, 1178 cm^−1^.

*3-O-Acetyl-28-O’-(3’,3’-dimethyl-3’-(5"-bromopentyloxycarbonyl)propanoyl)betulin (**6f**)* was obtained as a resin (99.0 mg, 52% yield); *R_f_ =* 0.23 (DCM:MeOH, 100:1); HRMS (ESI^+^) *m*/*z*: calcd for C_43_H_70_BrO_6_ ([M+H]^+^) 761.4356, found 761.4353; ^1^H NMR (600 MHz, CDCl_3_): δ_H_ 0.76, 0.77, 0.89, 0.95, 1.32 (all s, 3H each, H-23–H-27), 1.20 (s, 6H, CMe_2_), 1.61 (s, 3H, H-30), 0.65–1.92 (m, 30H, CH, CH_2_ *BN* scaffold and (CH_2_)_3_ fragment of linker), 1.97 (s, 3H, CH_3_CO), 2.35 (td, 1H, *J_1_* = 5.8 Hz, *J_2_* = 11.1 Hz, H-19), 2.56 (s, 2H, O(CO)CH_2_), 3.35 (t, 2H, *J* = 6.7 Hz, CH_2_Br), 3.78 (dd, 1H, *J_1_* = 1.3 Hz, *J_2_* = 11.1 Hz, H-28b), 4.02 (t, 2H, *J* = 6.5 Hz, (CO)OCH_2_), 4.17 (dd, 1H, *J_1_* = 2.2 Hz, *J_2_* = 11.0 Hz, H-28a), 4.39 (dd, 1H, *J_1_* = 5.6 Hz, *J_2_* = 10.8 Hz, H-3), 4.51 (s, br, 1H, H-29b), 4.61 (d, 1H, *J* = 2.3 Hz, H-29a) ppm; ^13^C NMR (150 MHz, CDCl_3_): δ_C_ 14.7, 16.0, 16.1, 16.5, 18.1, 19.1, 20.8, 21.3, 23.7, 24.6, 25.1, 25.5, 27.0, 27.7, 27.9, 29.5, 29.7, 32.2, 33.4, 34.1, 34.5, 37.0, 37.6, 37.8, 38.4, 40.6, 40.9, 42.7, 44.5, 46.3, 47.7, 48.8, 50.3, 55.4, 62.8, 64.3, 80.8, 109.9, 150.1, 171.0, 171.6, 176.6 ppm; IR (ATR) ν: 2941, 1730, 1245, 1105 cm^−1^.

#### 3.2.4. General Procedure for the Synthesis of Triphenylphosphonium Derivatives of BN (**7**, 28-TPP^⊕^ BN)

The bromide derivative of *BN* (**6**, 0.1 mmol, 1 eq.) and triphenylphosphine (0.2 mmol, 52.5 mg, 2 eq.) were dissolved in dry DCM (1.0–1.5 mL) and stirred at room temperature for 10–15 min until homogenization was reached. The solvent was evaporated under reduced pressure and the residue was heated in an oil bath at 120 °C under an Ar atmosphere for 6–12 h. The obtained mixture was washed with diethyl ether (**7a**–**7c**: 3 × 4 mL; **7d**–**7f**: 5 × 3 mL) at 50 °C. Then, the crude product was crystalized from ethyl acetate/diethyl ether (1:4, *v*/*v*) and dried under reduced pressure at 50 °C for 4 h.

*3-O-Acetyl-28-O’-(3’-(3"-triphenylphosphoniopropyloxycarbonyl)propanoyl)betulin bromide (**7a**)* was obtained as a resin (95.8 mg, 99% yield); HRMS (ESI^+^) *m*/*z*: calcd for C_57_H_76_O_6_P^+^ ([M]^+^) 887.5380, found 887.5383; ^1^H NMR (600 MHz, CDCl_3_): δ_H_ 0.83, 0.84, 0.85, 0.95, 0.96 (all s, 3H each, H-23–H-27), 1.67 (s, 3H, H-30), 0.70–1.95 (m, 26H, CH, CH_2_ *BN* scaffold and CH_2_ fragment of linker), 2.04 (s, 3H, CH_3_CO), 2.38 (td, 1H, *J_1_* = 5.7 Hz, *J_2_* = 0.8 Hz, H-19), 2.60-2.66 (m, 4H, O(CO)CH_2_CH_2_), 3.84 (d, 1H, *J* = 11.0 Hz, H-28b), 4.09–3.99 (m, 2H, CH_2_P), 4.22 (d, 1H, *J* = 11.0 Hz, H-28a), 4.39–4.45 (m, 2H, (CO)OCH_2_), 4.46 (dd, 1H, *J_1_* = 5.1 Hz, *J_2_* = 11.1 Hz, H-3), 4.59 (s, br, H-29b), 4.67 (s, br, H-29a), 7.71-7.90 (m, 15H, PPh_3_), ppm; ^13^C NMR (100 MHz, CDCl_3_): δ_C_ 14.7, 16.0, 16.1, 16.5, 18.1, 19.1, 19.5, 19.8 (d, *J_C,P_* 52.3 Hz), 20.8, 21.3, 22.3, 22.4, 23.6, 25.1, 27.0, 27.9, 29.1, 29.2, 29.5, 29.6, 34.0, 34.5, 37.0, 37.6, 37.8, 38.3, 40.8, 42.6, 46.4, 47.8, 48.7, 50.2, 55.3, 62.9, 63.4 (d, *J_C,P_* = 17.4 Hz), 80.8, 109.9, 118.1 (d, *J_C,P_* = 85.7 Hz), 130.5 (d, *J_C,P_* = 12.1 Hz), 133.8 (d, *J_C,P_* = 9.8 Hz), 135.0 (d, *J_C,P_* = 3.0 Hz), 150.0, 171.0, 172.0, 172.8 ppm; ^31^P NMR (162 MHz, CDCl_3_): δ_P_ 24.75 ppm; IR (ATR) ν: 2942, 1729, 1246, 1156, 691 cm^−1^.

*3-O-Acetyl-28-O’-(3’-(4"-triphenylphosphoniobutyloxycarbonyl)propanoyl)betulin bromide (**7b**)* was obtained as a resin (97.2 mg, 99% yield); HRMS (ESI^+^) *m*/*z*: calcd for C_58_H_78_O_6_P^+^ ([M]^+^) 901.5536, found 901.5550; ^1^H NMR (600 MHz, CDCl_3_): δ_H_ 0.83, 0.84, 0.85, 0.96, 1.00 (all s, 3H each, H-23–H-27), 1.68 (s, 3H, H-30), 0.72–1.97 (m, 26H, CH, CH_2_ *BN* scaffold and CH_2_ fragment of linker), 2.04 (s, 3H, CH_3_CO), 2.12 (q, 2H, *J* = 7.1 Hz, CH_2_ fragment of linker), 2.40 (td, 1H, *J_1_* = 5.8 Hz, *J_2_* = 11.0 Hz, H-19), 2.48–2.59 (m, 4H, O(CO)CH_2_CH_2_), 3.85 (d, 1H, *J* = 11.0 Hz, H-28b), 3.97–4.06 (m, 2H, CH_2_P), 4.14 (t, 2H, *J* = 5.8 Hz, (CO)OCH_2_), 4.24 (d, 1H, *J* = 11.1 Hz, H-28a), 4.47 (dd, 1H, *J_1_* = 5.3 Hz, *J_2_* = 11.0 Hz, H-3), 4.59 (s, br, 1H, H-29b) 4.67 (s, br, H-29a), 7.66–7.92 (m, 15H, PPh_3_) ppm; ^13^C NMR (100 MHz, CDCl_3_): δ_C_ 14.7, 16.0, 16.1, 16.5, 18.1, 19.1, 19.26, 19.33, 20.7, 20.8, 21.3, 22.2 (d, *J_C,P_* = 50.6 Hz), 23.7, 25.1, 27.0, 27.9, 29.1, 29.2, 29.5, 29.7, 34.1, 34.5, 37.0, 37.6, 37.8, 38.3, 40.9, 42.7, 46.4, 47.7, 48.7, 50.2, 55.3, 63.0, 63.6, 80.9, 109.9, 118.3 (d, *J_C,P_* = 84.9 Hz), 130.4 (d, *J_C,P_* = 12.1 Hz), 133.8 (d, *J_C,P_* = 9.9 Hz), 134.9 (d, *J_C,P_* = 3.0 Hz), 150.0, 171.0, 172.2, 172.6 ppm; ^31^P NMR (162 MHz, CDCl_3_): δ_P_ 24.61 ppm; IR (ATR) ν: 2945, 1731, 1246, 1156, 691 cm^−1^.

*3-O-Acetyl-28-O’-(3’-(5"-triphenylphosphoniopentyloxycarbonyl)propanoyl)betulin bromide (**7c**)* was obtained as a resin (77.7 mg, 78% yield); HRMS (ESI^+^): calcd for C_59_H_80_O_6_P^+^ ([M]^+^) *m*/*z*: 915.5693, found 915.5715; ^1^H NMR (600 MHz, CDCl_3_): δ_H_ 0.83, 0.84, 0.85, 0.96, 1.01 (all s, 3H each, H-23–H-27), 1.67 (s, 3H, H-30), 0.70–1.97 (m, 30H, CH, CH_2_ *BN* scaffold and (CH_2_)_3_ fragment of linker), 2.04 (s, 3H, CH_3_CO), 2.41 (td, 1H, *J_1_* = 5.8 Hz, *J_2_* = 11.1 Hz, H-19), 2.55–2.65 (m, 4H, O(CO)CH_2_CH_2_), 3.86 (d, 1H, *J* = 11.0 Hz, H-28b), 3.91–3.98 (m, 2H, CH_2_P), 4.03 (t, 2H, *J* = 6.4 Hz, (CO)OCH_2_), 4.25 (d, 1H, *J* = 11.1 Hz, H-28a), 4.47 (dd, 1H, *J_1_* = 5.4 Hz, *J_2_* = 11.0 Hz, H-3), 4.58 (s, br, H-29b), 4.67 (s, br, H-29a), 7.67–7.91 (m, 15H, PPh_3_) ppm; ^13^C NMR (100 MHz, CDCl_3_): δ_C_ 14.7, 16.0, 16.1, 16.5, 18.1, 19.1, 20.7, 21.3, 22.2, 22.3, 22.7 (d, *J_C,P_* = 49.4 Hz), 23.7, 25.1, 26.5, 26.7, 27.0, 27.9, 28.1, 29.1, 29.2, 29.5, 29.7, 34.1, 34.5, 37.0, 37.5, 37.8, 38.3, 40.8, 42.6, 46.4, 47.7, 48.7, 50.2, 55.3, 62.9, 65.8, 80.9, 109.8, 118.4 (d, *J_C,P_* = 85.0 Hz), 130.5 (d, *J_C,P_* = 12.1 Hz), 133.7 (d, *J_C,P_* = 9.8 Hz), 134.9 (d, *J_C,P_* = 3.0 Hz), 150.1, 171.0, 172.3, 172.7 ppm; ^31^P NMR (161.9 MHz, CDCl_3_): δ_P_ 24.37 ppm; IR (ATR) ν: 2946, 1731, 1246, 1157, 692 cm^−1^.

*3-O-Acetyl-28-O’-(3’,3’-dimethyl-3’-(3"-triphenylphosphoniopropyloxycarbonyl)propanoyl)betulin bromide (**7d**)* was obtained as a resin (71.7 mg, 72% yield); HRMS (ESI^+^) *m*/*z*: calcd for C_59_H_80_O_6_P^+^ ([M]^+^) 915.5693, found 915.5717; ^1^H NMR (600 MHz, CDCl_3_): δ_H_ 0.80, 0.83, 0.84, 0.87, 0.94 (all s, 3H each, H-23–H-27), 1.21 (s, 3H, CMe), 1.22 (s, 3H, CMe),1.66 (s, 3H, H-30), 0.68–2.08 (m, 26H, CH, CH_2_ *BN* scaffold and CH_2_ fragment of linker), 2.04 (s, 3H, CH_3_CO), 2.29 (td, 1H, *J_1_* = 5.8 Hz, *J_2_* = 10.8 Hz, H-19), 2.63–2.66 (m, 2H, 2 × O(CO)CH_2_), 3.76 (d, 1H, *J* = 11.0 Hz, H-28a), 4.01–4.10 (m, 3H, CH_2_P and H-28b), 4.45–4.48 (m, 3H, H-3 and (CO)OCH_2_), 4.58 (s, br, 1H, H-29b), 4.63 (s, br, 1H, H-29a), 7.69–7.91 (m, 15H, PPh_3_) ppm; ^13^C NMR (150 MHz, CDCl_3_): δ_C_ 14.7, 15.9, 16.1, 16.5, 18.1, 19.0, 19.4 (d, *J_C.P_* = 51.8 Hz), 20.8, 21.3, 22.4, 23.6, 25.1, 25.4, 25.5, 26.9, 27.9, 29.5, 29.7, 34.0, 34.5, 37.0, 37.6, 37.8, 38.3, 40.6, 40.8, 42.6, 44.3, 46.3, 47.8, 48.6, 50.2, 55.3, 62.6, 63.9 (d, *J_C,P_* = 18.4 Hz), 80.8, 109.9, 118.3 (d, *J_C.P_* = 86.3 Hz), 130.4 (d, *J_C,P_* = 12.6 Hz), 133.8 (d, *J_C.P_* = 10.4 Hz), 134.9 (d, *J_C,P_* = 0.5 Hz), 150.0, 171.0, 172.0, 176.7 ppm; ^31^P NMR (161.9 MHz, CDCl_3_): δ_P_ 19.38 ppm; IR (ATR) ν: 2946, 1727, 1248, 1177, 725, 690 cm^−1^.

*3-O-Acetyl-28-O’-(3’,3’-dimethyl-3’-(4"-triphenylphosphoniobutyloxycarbonyl)propanoyl)betulin bromide (**7e**)* was obtained as a resin (78.8 mg, 78% yield); HRMS (ESI^+^) *m*/*z*: calcd for C_60_H_82_O_6_P^+^ ([M]^+^) 929.5849, found 929.5861; ^1^H NMR (600 MHz, CDCl_3_): δ_H_ 0.75, 0.76, 0.77, 0.88, 0.89 (all s, 3H each, H-23–H-27), 1.051 (s, 3H, CMe), 1.054 (s, 3H, CMe), 1.60 (s, 3H, H-30), 0.68–1.90 (m, 26H, CH, CH_2_ *BN* scaffold, and CH_2_ fragment of linker), 1.97 (s, 3H, CH_3_CO), 2.08 (q 2H, *J* 6.7 Hz CH_2_ fragment of linker), 2.30 (td, 1H, *J_1_* = 5.7 Hz, *J_2_* = 10.8 Hz, H-19), 2.45 (s, 2H, O(CO)CH_2_), 3.72 (d, 1H, *J* = 11.0 Hz, H-28b), 3.87–3.92 (m, 2H, CH_2_P), 4.07–4.10 (m, 3H, H-28a and (CO)OCH_2_), 4.39 (dd, 1H, *J_1_* = 5.3 Hz, *J_2_* = 11.0 Hz, H-3), 4.51 (s, br, 1H, H-29b), 4.58 (d, 1H, *J* = 2.3 Hz, H-29a), 7.61–7.87 (m, 15H, PPh_3_) ppm; ^13^C NMR (150 MHz, CDCl_3_): δ_C_ 14.6, 16.0, 16.1, 16.4, 18.1, 19.0, 19.1, 20.7, 21.2, 22.1 (d, *J_C,P_* = 49.3 Hz), 23.6, 25.1, 25.3, 26.9, 27.9, 29.1, 29.2, 29.5, 29.6, 34.0, 34.4, 36.9, 37.5, 37.7, 38.3, 40.5, 40.8, 42.6, 44.2, 46.2, 47.6, 48.6, 50.2, 55.3, 62.7, 63.2, 80.8, 109.8, 118.3 (d, *J_C,P_* = 88.1 Hz), 130.4 (d, *J_C,P_* = 12.6 Hz), 133.7 (d, *J,_P_* = 10.4 Hz), 134.9 (d, *J_C,P_* = 0.5 Hz), 149.9, 171.0, 171.5, 176.5 ppm; ^31^P NMR (162 MHz, CDCl_3_): δ_P_ 24.52 ppm; IR (ATR) ν: 2948, 1725, 1248, 1179, 723, 690 cm^−1^.

*3-O-Acetyl-28-O’-(3’,3’-dimethyl-3’-(5"-triphenylphosphoniopentyloxycarbonyl)propanoyl)betulin bromide (**7f**)* was obtained as a resin (73.7 mg, 72% yield); HRMS (ESI^+^) *m*/*z*: calcd for C_61_H_84_O_6_P^+^ ([M]^+^) 943.6006, found 943.6042; ^1^H NMR (600 MHz, CDCl_3_): δ_H_ 0.82, 0.83, 0.84, 0.94, 0.97 (all s, 3H each, H-23–H-27), 1.20 (s, 3H, CMe), 1.21 (s, 3H, CMe), 1.66 (s, 3H, H-30), 0.67–1.96 (m, 30H, CH, CH_2_ *BN* scaffold and (CH_2_)_3_ fragment of linker), 2.03 (s, 3H, CH_3_CO), 2.38 (td, 1H, *J_1_* = 5.8 Hz, *J_2_* = 10.9 Hz, H-19), 2.57 (s, 2H, O(CO)CH_2_), 3.83 (d, 1H, *J* = 11.0 Hz, H-28b), 3.88–3.95 (m, 2H, CH_2_P), 4.01 (t, 2H, *J* = 6.7 Hz, (CO)OCH_2_), 4.17 (d, 1H, *J* = 11.0 Hz, H-28a), 4.46 (dd, 1H, *J_1_* = 5.5 Hz, *J_2_* = 10.5 Hz, H-3), 4.57 (s, br, 1H, H-29b), 4.64 (s, br, 1H, H-29a), 7.65–7.91 (m, 15H, PPh_3_) ppm; ^13^C NMR (100 MHz, CDCl_3_): δ_C_ 14.7, 16.0, 16.1, 16.5, 18.1, 19.1, 20.8, 21.3, 22.21, 22.25, 22.7 (d, *J_C,P_* = 50.1 Hz), 23.6, 25.1, 25.4, 25.5, 26.4, 26.6, 26.9, 27.9, 28.0, 29.5, 29.7, 34.1, 34.5, 37.0, 37.5, 37.8, 38.3, 40.5, 40.8, 42.6, 44.4, 46.2, 47.7, 48.7, 50.2, 55.3, 62.7, 63.9, 80.9, 109.8, 118.4 (d, *J_C,P_* = 85.8 Hz), 130.4 (d, *J_C,P_* = 12.4 Hz), 133.7 (d, *J_C,P_* = 9.9 Hz), 134.9 (d, *J_C,P_* = 3.0 Hz), 150.0, 171.0, 171.7, 176.6 ppm; ^31^P NMR (161.9 MHz, CDCl_3_): δ_P_ 24.35 ppm; IR (ATR) ν: 2948, 1727, 1247, 1181, 725, 690 cm^−1^.

#### 3.2.5. General Procedure for the Synthesis of 3,28-Bis(bromoalkoxycarbonyl)propanoyl)betulin (**8**)

*3,28-O,O′-Bis(3′-carboxypropanoyl)betulin* (**5**, 0.25 mmol, 160.6 mg, 1 eq.), DMF (2 mL/100 mg **5**) and MeCN (0.2 mL/100 mg **5**), the appropriate dibromoalkane (Br(CH_2_)_n_Br, n = 3, 4, 5; 1.5 mmol, 6 eq.) and K_2_CO_3_ (0.50 mmol, 69.1 mg, 2 eq.) were stirred at 50 °C for 18–20 h. The obtained mixture was diluted with cold water (10 × volume) and extracted with ethyl acetate (6 × 19 mL). The combined organic layers were washed with brine (2 × 90 mL), dried over MgSO_4_, and the solvent was evaporated under reduced pressure. Then, crude product **8** was washed with methanol (2 × 1.0 mL) and was further purified by column chromatography (DCM/MeOH, gradient: 100:1 to 50:1).

*3,28-O’,O′-Bis(3’-(3"-bromopropyloxycarbonyl)propanoyl)betulin (**8a**)* was obtained as a resin (143.8 mg, 65% yield); *R_f_ =* 0.23 (DCM:MeOH, 100:1); HRMS (ESI^+^) *m*/*z*: calcd for C_44_H_69_Br_2_O_8_ ([M+H]^+^) 833.3359, found 833.3360; ^1^H NMR (600 MHz, CDCl_3_): δ_H_ 0.83, 0.84, 0.85, 0.97, 1.02 (all s, 3H each, H-23–H-27), 1.68 (s, 3H, H-30), 0.74–2.03 (m, 24H, CH, CH_2_ *BN* scaffold), 2.15–2.21 (m, 4H, 2 × CH_2_ fragment of linker), 2.43 (td, 1H, *J_1_* = 5.8 Hz, *J_2_* = 11.1 Hz, H-19), 2.61–2.68 (m, 8H, 2 × O(CO)CH_2_CH_2_), 3.45–3.47 (m, 4H, 2 × CH_2_Br), 3.87 (d, 1H, *J* = 10.3 Hz, H-28b), 4.22–4.26 (m, 4H, 2 × (CO)OCH_2_), 4.29 (dd, 1H, *J_1_* = 1.9 Hz, *J_2_* = 11.0 Hz, H-28a), 4.46–4.51 (m, 1H, H-3), 4.59 (s, br, 1H, H-29b), 4.68 (d, 1H, *J* = 2.1 Hz, H-29a) ppm; ^13^C NMR (150 MHz, CDCl_3_): δ_C_ 14.7, 16.0, 16.1, 16.5, 18.1, 19.1, 20.8, 23.6, 25.1, 27.0, 27.9, 29.10, 29.16, 29.2, 29.3, 29.5, 29.6, 29.7, 31.6, 31.7, 34.1, 34.5, 37.0, 37.6, 37.8, 38.3, 40.9, 42.7, 46.4, 47.7, 48.8, 50.3, 55.4, 62.39, 62.43, 63.1, 81.4, 109.9, 150.1, 171.9, 172.1, 172.2, 172.5 ppm; IR (ATR) ν: 2943, 1732, 1157 cm^−1^.

*3,28-O,O′-Bis(3’-(4"-bromobutyloxycarbonyl)propanoyl)betulin (**8b**)* was obtained as a resin (159.8 mg, 70% yield); *R_f_ =* 0.26 (DCM:MeOH, 100:1); HRMS (ESI^+^) *m*/*z*: calcd for C_46_H_72_Br_2_O_8_Na([M+Na]^+^) 933.3492, found 933.3525; ^1^H NMR (600 MHz, CDCl_3_): δ_H_ 0.83, 0.84, 0.85, 0.97, 1.02 (all s, 3H each, H-23–H-27), 1.68 (s, 3H, H-30), 0.70–2.06 (m, 32H, CH, CH_2_ *BN* scaffold and 2 × (CH_2_)_2_ fragment of linker), 2.43 (td, 1H, *J_1_* = 5.7 Hz, *J_2_* = 11.1 Hz, H-19), 2.60–2.68 (m, 8H, 2 × O(CO)CH_2_CH_2_), 3.43–3.45 (m, 4H, 2 × CH_2_Br), 3.87 (d, 1H, *J* = 11.0 Hz, H-28b), 4.11–4.13 (m, 4H, 2 × (CO)OCH_2_), 4.29 (d, 1H, *J* = 11.0 Hz, H-28a), 4.48 (dd, 1H, *J_1_* = 5.8 Hz, *J_2_* = 10.5 Hz, H-3), 4.58 (s, br, 1H, H-29b), 4.68 (s, br, 1H, H-29a) ppm; ^13^C NMR (150 MHz, CDCl_3_): δ_C_ 14.6, 15.9, 16.0, 16.4, 18.0, 19.0, 20.7, 23.5, 25.0, 25.1, 26.9, 27.1, 27.8, 29.1, 29.0, 29.41, 29.44, 29.6, 32.9, 34.0, 34.4, 36.9, 37.4, 37.7, 38.2, 40.8, 42.6, 46.3, 47.6, 48.7, 50.1, 55.3, 62.9, 63.5, 63.6, 81.2, 109.8, 149.9, 171.8, 172.09, 172.14, 172.4 ppm; IR (ATR) ν: 2944, 1730, 1157 cm^−1^.

*3,28-O,O′-Bis(3’-(5"-bromopentyloxycarbonyl)propanoyl)betulin (**8c**)* was obtained as a resin (150.5 mg, 64% yield); *R_f_ =* 0.32 (DCM:MeOH, 100:1); HRMS (ESI^+^) *m*/*z*: calcd for C_48_H_76_Br_2_O_8_Na ([M+Na]^+^) 961.3805, found 961.3886; ^1^H NMR (600 MHz, CDCl_3_): δ_H_ 0.83, 0.84, 0.85, 0.97, 1.02 (all s, 3H each, H-23–H-27), 1.68 (s, 3H, H-30), 0.72–2.00 (m, 36H, CH, CH_2_ *BN* scaffold and 2 × (CH_2_)_3_ fragment of linker), 2.43 (td, 1H, *J_1_* = 5.8 Hz, *J_2_* = 11.1 Hz, H-19), 2.60–2.68 (m, 8H, 2 × O(CO)CH_2_CH_2_), 3.40–3.42 (m, 4H, 2 × CH_2_Br), 3.87 (d, 1H, *J* = 12.2 Hz, H-28b), 4.08–4.11 (m, 4H, 2 × (CO)OCH_2_), 4.29 (dd, 1H, *J_1_* = 2.0 Hz, *J_2_* = 11.2 Hz, H-28a), 4.49 (dd, 1H, *J_1_* = 5.5 Hz, *J_2_* = 10.9 Hz, H-3), 4.59 (s, br, 1H, H-29b), 4.68 (d, 1H, *J* = 2.2 Hz, H-29a) ppm; ^13^C NMR (150 MHz, CDCl_3_): δ_C_ 14.7, 16.0, 16.1, 16.5, 18.1, 19.1, 20.8, 23.6, 24.6, 25.1, 27.0, 27.7, 27.9, 28.1, 28.2, 29.2, 29.5, 29.3, 29.7, 32.3, 33.4, 34.1, 34.5, 37.0, 37.6, 37.8, 38.3, 40.9, 42.7, 46.4, 47.7, 48.8, 50.2, 55.4, 63.0, 64.3, 64.4, 81.3, 109.9, 150.1, 171.9, 172.25, 172.32, 172.6 ppm; IR (ATR) ν: 2943, 1731, 1157 cm^−1^.

#### 3.2.6. General Procedure for the Synthesis of Bis(triphenylphoshonium) Derivatives of BN (**9**, 3,28-bisTPP^⊕^ BN)

3,28-*O*,*O*′-*Bis*(3’-(3"-bromoalkoxycarbonyl)propanoyl)betulin (**8a**–**8c**) (0.1 mmol, 1 eq.) and triphenylphosphine (0.3 mmol, 78.7 mg, 3 eq.) were dissolved in dry DCM (1–2 mL) and stirred at room temperature for 10–15 min until homogenization was reached. The solvent was evaporated under reduced pressure, and the residue was heated in an oil bath at 120 °C under an Ar atmosphere. The obtained mixture was washed with diethyl ether (5 × 4 mL) at 50 °C. Then, the crude product was crystalized from ethyl acetate/diethyl ether (1:4, *v*/*v*) and dried under reduced pressure at 50 °C for 6 h or purified using column chromatography (DCM:MeOH, 10:1, *v*/*v*).

*3,28-O,O′-Bis(3’-(3"-triphenylphosphoniopropyloxycarbonyl)propanoyl)betulin bromide (**9a**)* was obtained as a resin (74.7 mg, 53% yield); *R_f_ =* 0.13 (DCM:MeOH, 10:1); HRMS (ESI^+^) *m*/*z*: calcd for C_40_H_49_O_4_P^2+^ ([M]^2+^) 624.3368, found 624.3379; ^1^H NMR (600 MHz, CDCl_3_): δ_H_ 0.70, 0.72, 0.73, 0.88, 0.89 (all s, 3H each, H-23–H-27), 1.60 (s, 3H, H-30), 0.60–1.90 (m, 24H, CH, CH_2_ *BN* scaffold), 1.92–2.00 (m, 4H, 2 × CH_2_ fragment of linker), 2.31 (td, 1H, *J_1_* = 5.7 Hz, *J_2_* = 10.9 Hz, H-19), 2.51–2.60 (m, 8H, 2 × O(CO)CH_2_CH_2_), 3.75 (d, 1H, *J* = 10.8 Hz, H-28b), 3.92–3.99 (m, 4H, 2 × CH_2_P), 4.17 (dd, 1H, *J_1_* = 1.9 Hz, *J_2_* = 11.0 Hz, H-28a), 4.31 (dd, 1H, *J_1_* = 5.7 Hz, *J_2_* = 10.4 Hz, H-3), 4.34–4.38 (m, 4H, 2 × (CO)OCH_2_), 4.52 (s, br, 1H, H-29b), 4.60 (d, 1H, *J* = 2.2 Hz, H-29a), 7.63–7.83 (m, 30H, 2 × PPh_3_) ppm; ^13^C NMR (150 MHz, CDCl_3_): δ_C_ 14.7, 16.0, 16.1, 16.5, 18.1, 19.1, 19.7 (d, *J_C,P_* = 51.8 Hz), 20.8, 22.3, 23.6, 25.1, 27.0, 28.0, 29.1, 29.5, 29.4, 29.7, 34.1, 34.5, 37.0, 37.6, 37.8, 38.3, 40.9, 42.7, 46.4, 47.8, 48.7, 50.3, 55.4, 62.9, 63.5 (d, *J_C,P_* = 18.4 Hz), 81.3, 109.9, 118.1 (d, *J_C,P_* = 86.3 Hz), 130.5 (d, *J_C,P_* = 12.8 Hz), 133.8 (d, *J_C,P_* = 9.2 Hz), 135.1 (d, *J_C,P_* = 0.5 Hz), 150.0, 171.99, 172.0, 172.1, 172.7 ppm; ^31^P NMR (162 MHz, CDCl_3_): δ_P_ 24.61, 24.54 ppm; IR (ATR) ν: 2946, 1730, 1438, 1158, 724, 691 cm^−1^.

*3,28-O,O’-Bis(3’-(4"-triphenylphosphoniobutyloxycarbonyl)propanoyl)betulin bromide (**9b**)* was obtained as a resin (115.0 mg, 80% yield); *R_f_ =* 0.19 (DCM:MeOH, 10:1); HRMS (ESI^+^) *m*/*z*: calcd for C_41_H_51_O_4_P^2+^ ([M]^2+^) 638.3525, found 638.3536; ^1^H NMR (600 MHz, CDCl_3_): δ_H_ 0.73, 0.75, 0.89, 0.93, 1.18 (all s, 3H each, H-23–H-27), 1.61 (s, 3H, H-30), 0.60–2.05 (m, 32H, CH, CH_2_ *BN* scaffold and 2 × (CH_2_)_2_ fragment of linker), 2.38 (td, 1H, *J_1_* = 5.6 Hz, *J_2_* = 10.9 Hz, H-19), 2.41–2.52 (m, 8H, 2 × O(CO)CH_2_CH_2_), 3.77 (d, 1H, *J* = 10.1 Hz, H-28b), 3.89–3.97 (m, 4H, 2 × CH_2_P), 4.05–4.08 (m, 4H, 2 × (CO)OCH_2_), 4.22 (d, 1H, *J_1_* = 11.4 Hz, H-28a), 4.35 (m, 1H, H-3), 4.52 (s, br, 1H, H-29b), 4.61 (d, 1H, *J* = 3.1 Hz, H-29a), 7.58–7.87 (m, 30H, 2 × PPh_3_) ppm; ^13^C NMR (100 MHz, CDCl_3_): δ_C_ 14.7, 16.0, 16.1, 16.5, 18.1, 19.1, 19.2, 19.3, 20.8, 22.2, (d, *J_C,P_* = 50.0 Hz), 23.6, 25.1, 25.3, 27.0, 27.9, 28.9, 29.0, 29.1, 29.5, 29.6, 34.0, 34.5, 37.0, 37.5, 37.8, 38.3, 40.8, 42.7, 46.4, 47.7, 48.7, 50.2, 55.4, 63.0, 63.4, 81.3, 109.9, 118.2 (d, *J_C,P_* = 88.8 Hz), 130.4 (d, *J_C,P_* = 12.9 Hz), 133.7 (d, *J_C,P_* = 9.9 Hz), 135.0 (d, *J_C,P_* = 3.0 Hz), 150.0, 171.9, 172.16, 172.18, 172.6 ppm; ^31^P NMR (162 MHz, CDCl_3_): δ_P_ 24.51 ppm; IR (ATR) ν: 2936, 1728, 1438, 1160, 725, 691 cm^−1^.

*3,28-O,O′-Bis(3’-(5"-triphenylphosphoniopentyloxycarbonyl)propanoyl)betulin bromide (**9c**)* was obtained as a resin (129.0 mg, 88% yield); *R_f_ =* 0.11 (DCM:MeOH, 10:1); HRMS (ESI^+^) *m*/*z*: calcd for C_42_H_53_O_4_P^2+^ ([M]^2+^) 652.3681, found 652,3667; ^1^H NMR (400 MHz, CDCl_3_): δ_H_ 0.73, 0.74, 0.87, 0.92, 1.17 (all s, 3H each, H-23–H-27), 1.59 (s, 3H, H-30), 0.60–1.97 (m, 36H, CH, CH_2_ *BN* scaffold and 2 × (CH_2_)_3_ fragment of linker), 2.33 (td, 1H, *J_1_* = 5.7 Hz, *J_2_* = 10.8 Hz, H-19), 2.44–2.57 (m, 8H, 2 × O(CO)CH_2_CH_2_), 3.77 (d, 1H, *J* = 11.4 Hz, H-28b), 3.78–3.88 (m, 4H, 2 × CH_2_P), 3.89–3.98 (m, 4H, 2 × (CO)OCH_2_), 4.18 (d, 1H, *J* = 11.3 Hz, H-28a), 4.37 (dd, 1H, *J* = 8.0 Hz, H-3), 4.50 (s, br, 1H, H-29b), 4.59 (s, br, 1H, H-29a), 7.60–7.82 (m, 30H, 2 × PPh_3_) ppm; ^13^C NMR (100 MHz, CDCl_3_): δ_C_ 14.7, 16.0, 16.1, 18.5, 18.1, 19.1, 20.8, 22.2, 22.3, 22.8 (d, *J_C,P_* = 49.3 Hz), 23.6, 25.1, 26.7, 27.9, 28.1, 29.1, 29.2, 29.5, 29.7, 34.1, 34.5, 37.0, 37.6, 37.8, 38.3, 40.9, 42.7, 46.4, 47.7, 48.8, 50.2, 55.4, 63.0, 64.00, 64.04, 81.2, 109.9, 118.4 (d, *J_C,P_* = 85.0 Hz), 130.5 (d, *J_C,P_* = 12.2 Hz), 133.8 (d, *J_C,P_* = 9.8 Hz), 135.0 (d, *J_C,P_* = 3.0 Hz), 150.1, 172.0, 172.28, 172.30, 172.6 ppm; ^31^P NMR (162 MHz, CDCl_3_): δ_P_ 24.36 ppm.

### 3.3. Biological Evaluation

#### 3.3.1. Cytotoxicity Assay

##### Cell Lines

The human colorectal carcinoma cell line (HCT 116) and the human breast adenocarcinoma cell line (MCF-7) cells were purchased from the American Type Culture Collection (ATCC, Manassas, VA, USA). The Normal Human Dermal Fibroblast (NHDF) cells were purchased from Lonza (Dermal Fibroblasts, Lonza, Poland). All cells were cultured under standard conditions at 37 °C in a humidified atmosphere at 5% CO_2_ in DMEM/F12 medium (PAA) supplemented with 10% of heat-inactivated fetal bovine serum (FBS, EURx, Gdansk, Poland) and antibiotics (penicillin/streptomycin).

##### Cell Viability Assay

Cells were seeded at 7500 (HCT 116) or 10,000 (MCF-7, NHDF) cells/well in 96-well plates. After 24 h, the culture medium was removed and 100 µL of fresh medium containing the test compounds at 0–12.5 µM concentrations was added to the culture wells. The test compounds were dissolved in DMSO to obtain a stock solution with a concentration of 5 mM (betulin) or 10 mM (other test compounds). The stock solution was diluted with the fresh culture medium to the desired concentration. Controls were cells grown in medium without the addition of test compounds. After 24 h of incubation with the test compounds, 10 µL of CCK-8 reagent (Bimake, Houston, TX, USA) was added to each well. After 2 h, the absorbance of the samples was measured at a wavelength of 450 nm using a microplate reader (Epoch, BioTek Instruments, Winooski, VT, USA). The determinations were conducted in at least three biological replications (each biological replication contained 3 technical replications). The cell viability rate was calculated using CalcuSyn software (version 2.0, Biosoft, Cambridge, UK).

#### 3.3.2. Antibacterial Assay

Antibacterial analysis was performed using the *S. aureus ATCC 25923*, *S. epidermidis ATCC 12228*, and *Escherichia coli ATCC 25922* bacteria strains. The test compounds were dissolved in DMSO to obtain a stock solution with a concentration of 5 mM (betulin) or 10 mM (other test compounds). The stock solution was diluted with water to the desired concentration (25–250 µM). Then, 1 mL of the investigated solutions was mixed with 1 mL of culture medium (TSB, Biomaxima, Lublin, Poland) in a sterile glass tube. The initial concentration of bacteria was around 5 × 106 CFU/mL. The concentration of bacteria was measured using an optical densitometer before and after 18 h of bacteria culture in glass tubes at 37 °C (incubator POL-EKO, Wodzislaw Slaski, Poland). The investigations were repeated for three independent samples. The control sample was the culture medium without any supplementation.

## 4. Conclusions

In conclusion, we designed and synthesized nine new molecular hybrids of *BN* by covalent linkage of the alkyltriphenylphosphonium moiety to the parent skeleton via the linker O(CO)CH_2_CR_2_COO. We developed a few-stage methodology that enabled the preparation of both *mono*- and *bis*(TPP^⊕^) derivatives from easily available, cheap, natural active substance (*BN*) by simple transformations in high yields. The advantage of this protocol are the simple synthetic procedures and easy purification of the final products.

As expected, the triphenylphosphonium derivatives of *BN* showed a greater cytotoxicity than natural *BN* toward the cell lines tested (HCT 116 and MCF-7). Importantly, analogs (**7a**–**7c**) with one triphenylphoshonium cation were almost twice less toxic against healthy cells (NHDF), which demonstrated their selectivity. TPP^⊕^-conjugates with *BN* showed antimicrobial properties against the Gram-positive reference *S. aureus ATCC 25923* and *S. epidermidis ATCC 12228* bacteria when their concentration in the water solution was 200 µM.

The obtained results show that the bioavailability of natural *BN* can be improved by combining its backbone via linkers with a mitochondria-targeted TPP^⊕^ moiety. Additionally, our study provides important data about the properties of *BN* conjugates with TPP^⊕^ and encourages further research on the structural modifications of the parent *BN* skeleton.

## Figures and Tables

**Table 1:** The chemical shifts and coupling constants characteristic of TPP^⊕^ moiety in the synthesized triphenylphosphonium analogs of *BN* (**7** and **9**).

	^13^C NMR (CDCl_3_, TMS, δ (ppm)/*J*_C-P_ (Hz)				
		TPP^⊕^			
	*C*H_2_P^⊕^	C*^ipso^*	C*^meta^*	C*^ortho^*	C*^para^*
7a	19.8/52.3	118.1/85.7	130.5/12.1	133.8/9.8	135.0/3.0
7b	22.2/50.6	118.3/84.9	130.4/12.1	133.8/9.9	134.9/3.0
7c	22.7/49.4	118.4/85.0	130.5/12.1	133.7/9.8	134.9/3.0
7d	19.4/51.8	118.3/86.3	130.4/12.6	133.8/10.4	134.9/0.5
7e	22.1/49.3	118.3/88.1	130.4/12.6	133.7/10.4	134.9/0.5
7f	22.7/50.1	118.4/85.8	130.4/12.4	133.7/9.9	134.9/3.0
9a	19.7/51.8	118.1/86.3	130.5/12.8	133.8/9.2	135.1/0.5
9b	22.2/50.0	118.2/88.8	130.4/12.9	133.7/9.9	135.0/3.0
9c	22.8/49.3	118.4/85.0	130.5/12.2	133.8/9.8	135.0/3.0
δ, ppm	19.4–22.8	118.1–118.4	130.4–130.5	133.7–133.8	134.9–135.0
*J*_C-P,_ Hz	49.3–52.3	84.9–88.1	12.1–12.9	9.2–10.4	0.5–3.0

**Table 2:** A summary of the cytotoxic effects of *BN,* 28-TPP^⊕^ BN (**7**), and 3,28-*bis*TPP^⊕^ BN (**9**) on the HCT 116 and MCF-7 cancer cell lines and NHDF.

No.	R	n	Activity IC_50_, µM ^a,b^		
			*HCT 116*	*MCF-7*	*NHDF*
*BN*	**−**	−	neg	neg	neg
7a	H	3	5.56 ± 0.28	13.71± 0.54	9.68 ± 0.27
7b	H	4	5.77 ± 0.27	14.35 ± 0.38	10.71 ± 0.25
7c	H	5	6.48 ± 0.04	15.52 ± 0.92	11.29 ± 0.53
7f	Me	5	12.71 ± 0.89	50.47 ± 3.92	10.03 ± 0.48
9a	H	3	6.32 ± 0.27	31.30 ± 3.02	5.91 ± 0.33
9b	H	4	7.97 ± 0.51	23.60 ± 0.33	8.02 ± 0.35
9c	H	5	18.99 ± 0.51	53.30 ± 5.41	10.60 ± 0.34

^a^ Cytotoxicity was evaluated using the CCK-8 assay; ^b^ Incubation time 24 h; Data are presented as the mean ± standard deviation (n = 3); neg: no activity in the concentration range used.

**Table 3:** The results of the antimicrobial analysis using Gram-positive and Gram-negative bacteria cultured with the investigated compounds at 37 °C for 18 h. The results are presented as the differences between the optical density measurements of the samples before and after culture (*McFarland’s scale* (CFU/mL).

No.	*S. aureus* *ATCC 25923*		*S. epidermidis* *ATCC 12228*		*Escherichia coli* *ATCC 25922*	
	200 μM	250 μM	200 μM	250 μM	200 μM	250 μM
*BN*	5.1	5.6	neg	4.7	neg	neg
7a	0.9	0	neg	0	neg	neg
7b	2.2	0	neg	0	neg	neg
7c	1.0	0.1	neg	0.7	neg	neg
7d	0	0	0	0	neg	neg
7e	0	0	0	0	neg	neg
7f	0	0	0	0	neg	neg
9a	0	0	0	0	neg	neg
9b	0	0	0	0	neg	neg
9c	0	0	0	0	neg	neg
Control	7.5	7.5	4.1	4.9	5.1	5.0

0: no difference between samples after 18 h of bacteria culture (bacteria grow was inhibited); neg: negative results (analog BN did not inhibit bacteria growing).

## Data Availability

Not applicable.

## Supplementary Materials

The following can be downloaded at: https://www.mdpi.com/article/10.3390/molecules27165156/s1. Supporting information includes the ^1^H, ^13^C, ^31^P NMR spectra of betulin and all of the synthesized compounds (**1**–**9**) as well as the gHSQC and FTIR for the selected compounds.

## Sample Availability

Samples of compounds **7** and **9** are available from the authors.
