# Supplementary material for: Design, Synthesis and Preliminary Evaluation of the Cytotoxicity and Antibacterial Activity of Novel Triphenylphosphonium Derivatives of Betulin
Source: Molecules. 2022 Aug 12;27(16):5156. doi: 10.3390/molecules27165156 (PMC9416257; doi:10.3390/molecules27165156)
Supplement: Supplementary file 1 [file molecules-27-05156-s001.zip › molecules-1851072-supplementary.pdf]

Supporting Information

Design, Synthesis and Preliminary Evaluation of the Cytotoxicity and Antibacterial Activity of Novel Triphenylphosphonium Derivatives of Betulin

Mirosława Grymel[1,2,3*], Anna Lalik[3,4], Alicja Kazek-Kęsik[5], Marietta Szewczyk[1], Patrycja Grabiec[1], and Karol Erfurt[2]

1 Department of Organic Chemistry, Bioorganic Chemistry and Biotechnology, Silesian University of Technology, B. Krzywoustego 4, 44-100 Gliwice, Poland
2 Department of Chemical Organic Technology and Petrochemistry, Silesian University of Technology, B. Krzywoustego 4, 44-100 Gliwice, Poland
3 Biotechnology Center, Silesian University of Technology, B. Krzywoustego 8, 44-100 Gliwice, Poland
4 Department of Systems Biology and Engineering, Silesian University of Technology, Akademicka 16, 44-100 Gliwice, Poland
5 Department of Inorganic, Analytical Chemistry and Electrochemistry, Faculty of Chemistry, Silesian University of Technology, Krzywoustego 6, 44-100 Gliwice, Poland

* Correspondence: miroslawa.grymel@polsl.pl; Tel.: +48 032-237-1873 (M.G.)
Received: date; Accepted: date; Published: date

TABLE OF CONTENTS

^{1}H, ^{13}C NMR spectra of betulin and analogues **2-5**……………………..……………………….........…...S2-S10
^{1}H, ^{13}C NMR spectra of bromides of betulin **6a-6f**…………………..……………………….....……….S11-S23
^{1}H, ^{13}C, ^{31}P NMR spectra of triphenylphosphonium derivatives of betulin **7a-7f**…………….........S24-S42
^{1}H, ^{13}C NMR spectra of bromides of betulin **8a-8c** …………………..……………………….....……..S43-S48
^{1}H, ^{13}C, ^{31}P NMR spectra of *bis*triphenylphosphonium derivatives of betulin **9a-9c..**……………...S49-S57
FT-IR spectra for selected compounds……………………..……………………….....……...……..S58-S72

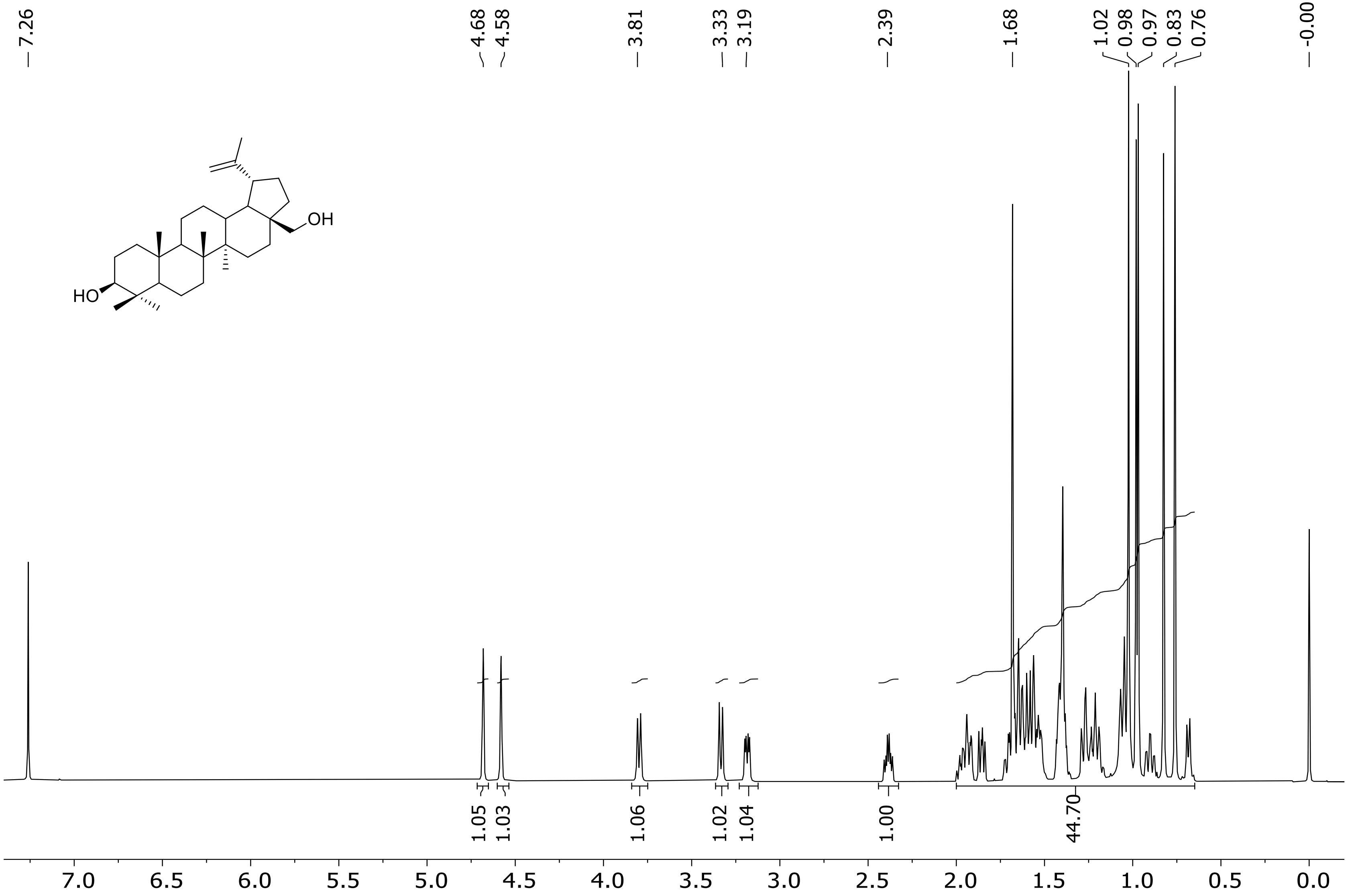

^{1}H NMR spectrum of betulin (**1**); 600 MHz/$CDCl_3$/TMS; δ (ppm).

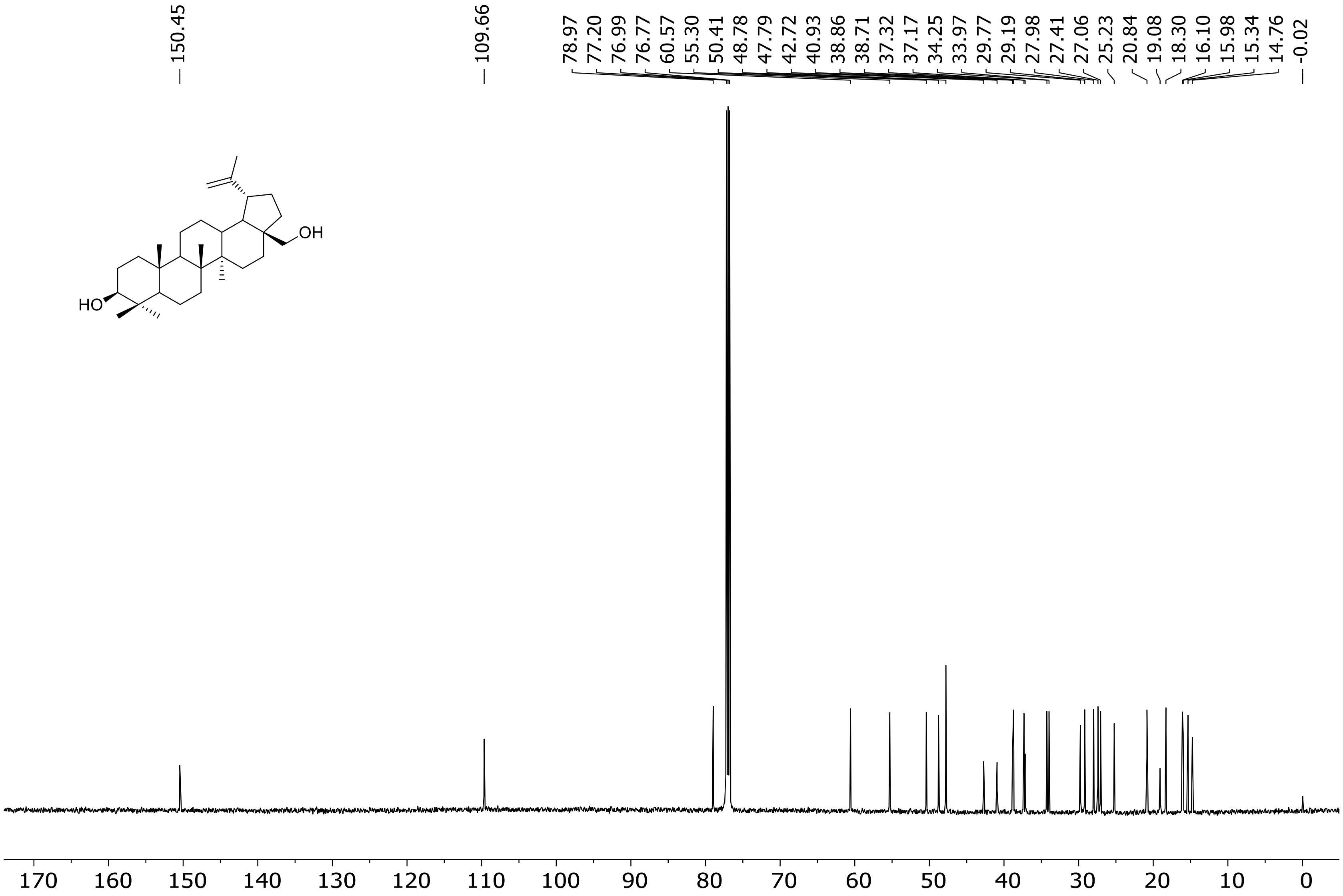

^{13}C NMR spectrum of betulin (**1**); 150 MHz/$CDCl_3$/TMS; δ (ppm).

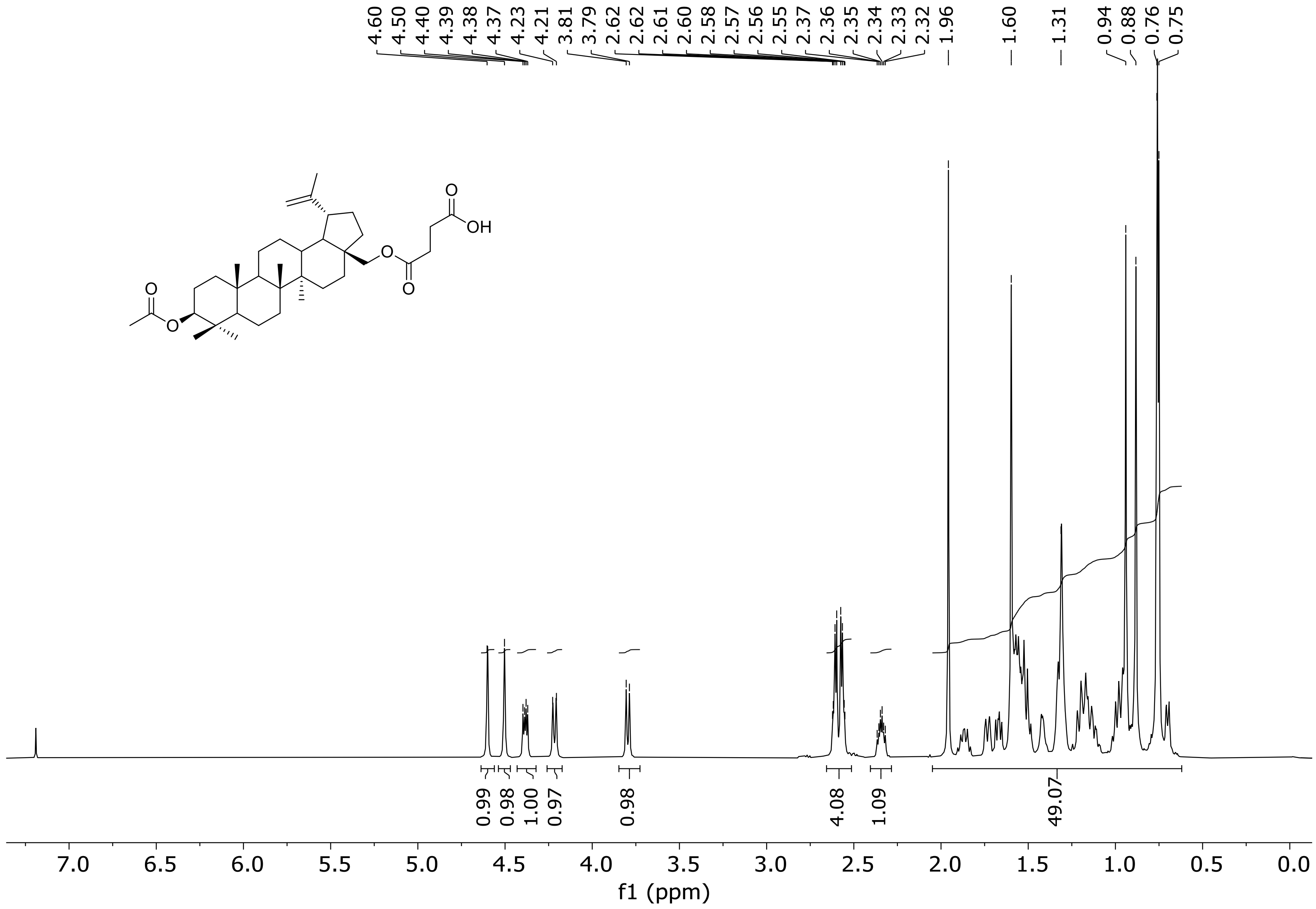

^{1}H NMR spectrum of 3-*O*-acetyl-28-*O*-(3'-carboxypropanoyl)betulin (**4a**); 600 MHz/$CDCl_3$/TMS; δ (ppm).

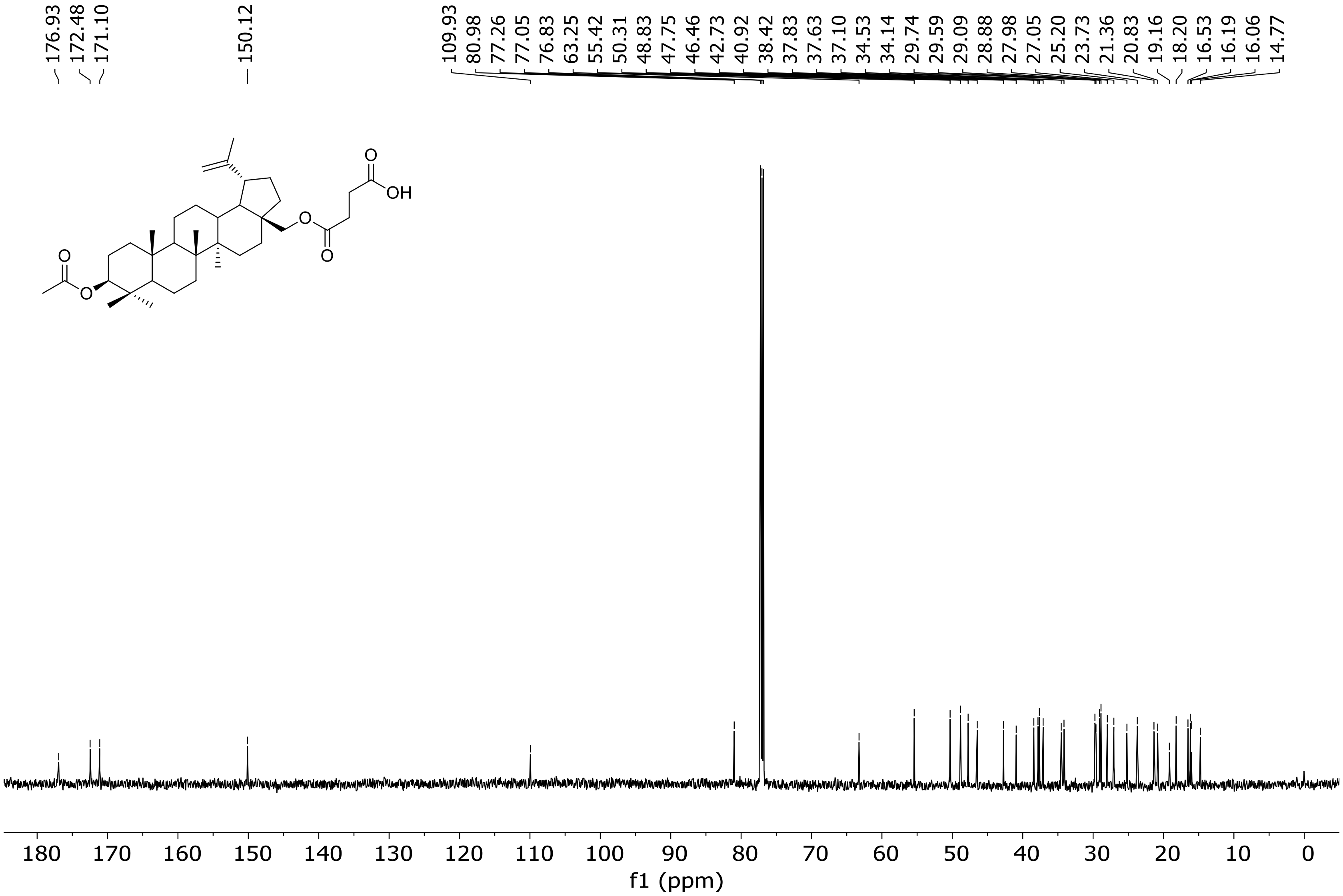

^{13}C NMR spectrum of 3-*O*-acetyl-28- *O*-(3'-carboxypropanoyl)betulin (**4a**); 150 MHz/$CDCl_3$/TMS; δ (ppm).

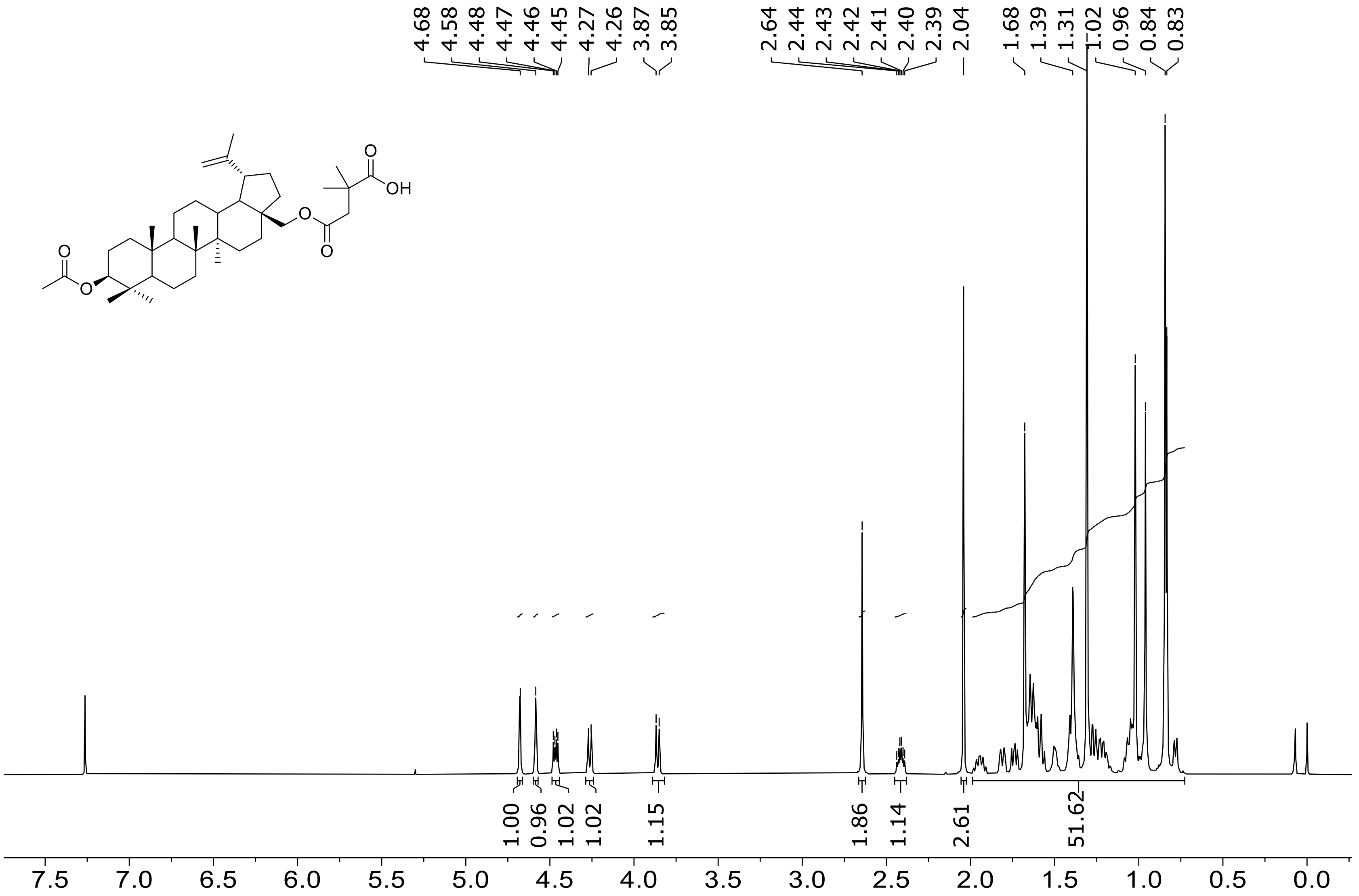

^{1}H NMR spectrum of 3-*O*-acetyl-28-*O*-(3',3'-dimethyl-3'-carboxypropanoyl)betulin (**4b**); 600 MHz/$CDCl_3$/TMS; δ (ppm).

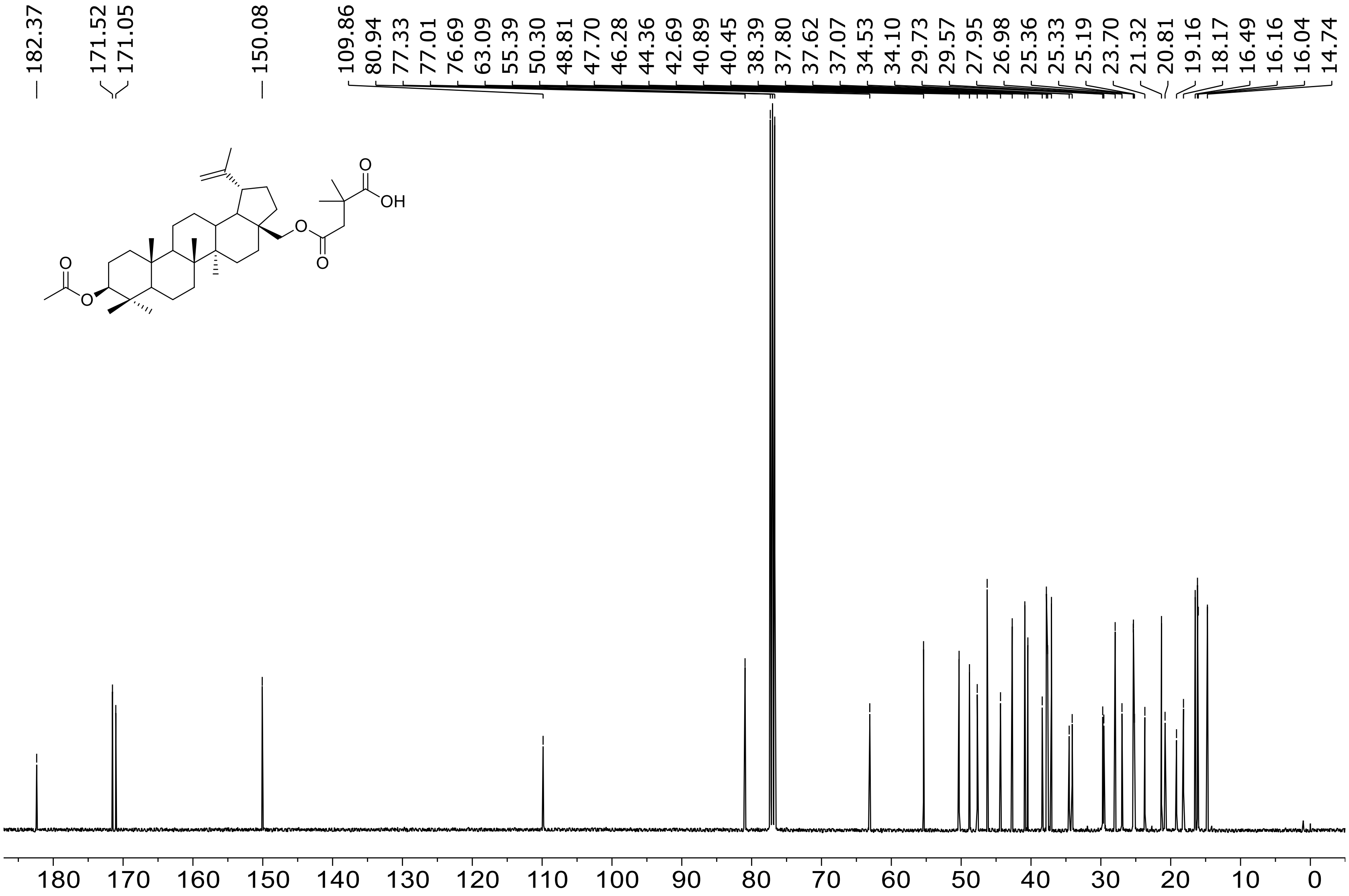

^{13}C NMR spectrum of 3- *O*-acetyl-28- *O*-(3',3'-dimethyl-3'-carboxypropanoyl)betulin (**4b**); 150 MHz/$CDCl_3$/TMS; δ (ppm).

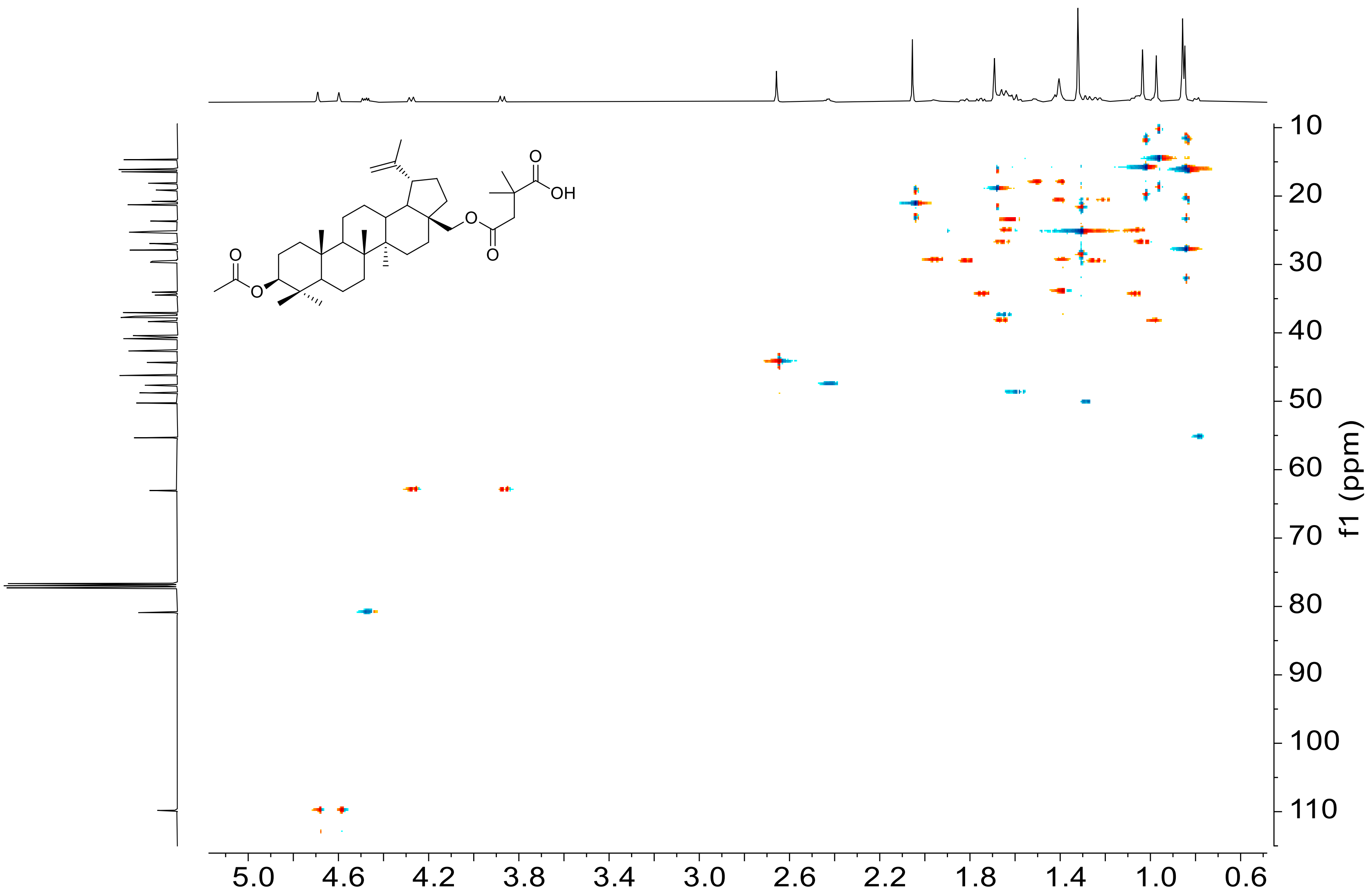

gHSQC spectrum of 3-*O*-acetyl-28-*O*-(3',3'-dimethyl-3'-carboxypropanoyl)betulin (**4b**)

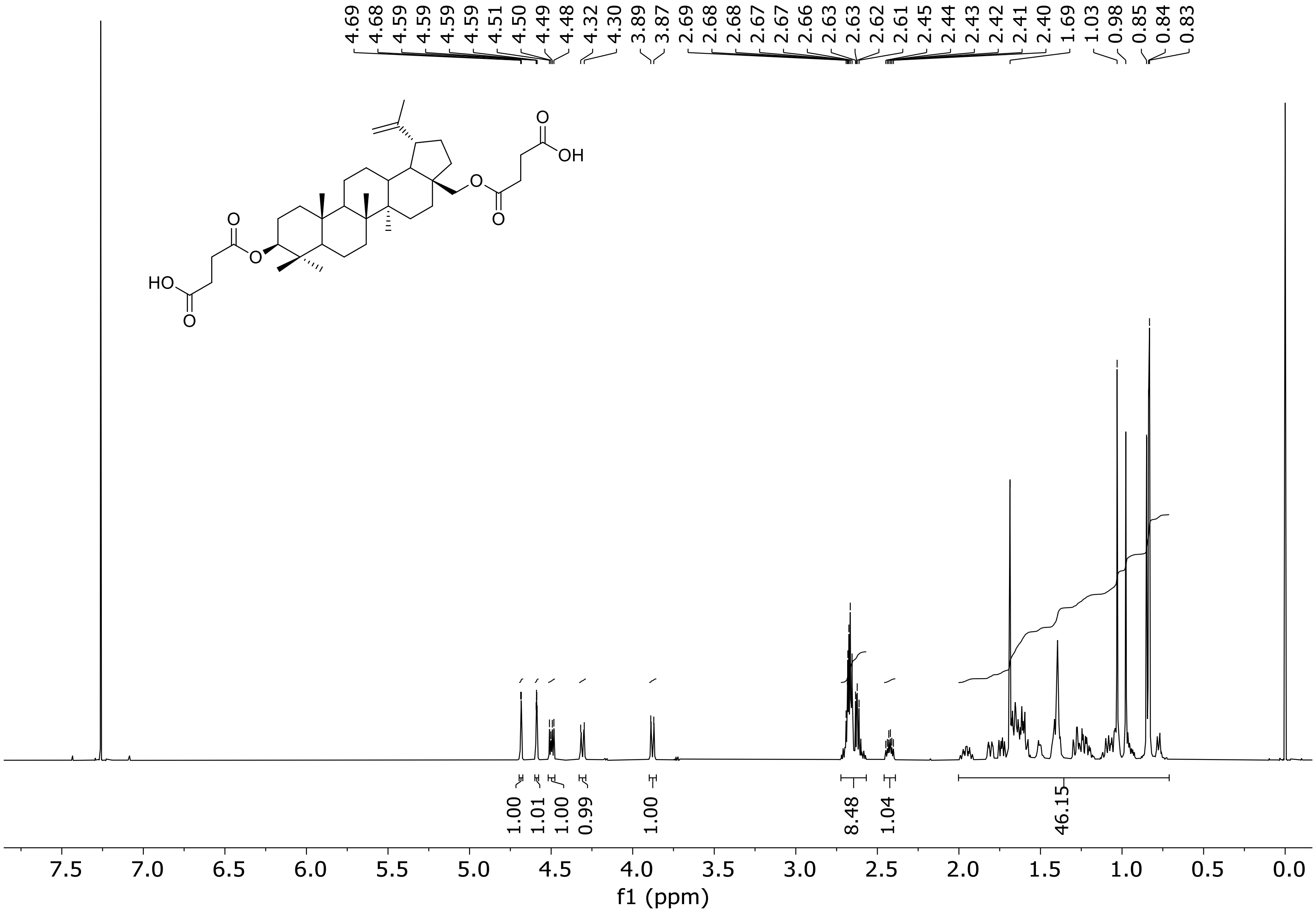

^{1}H NMR spectrum of 3,28-*O*,*O*′-bis(3′-carboxypropanoyl)betulin (**5**); 600 MHz/$CDCl_3$/TMS; δ (ppm).

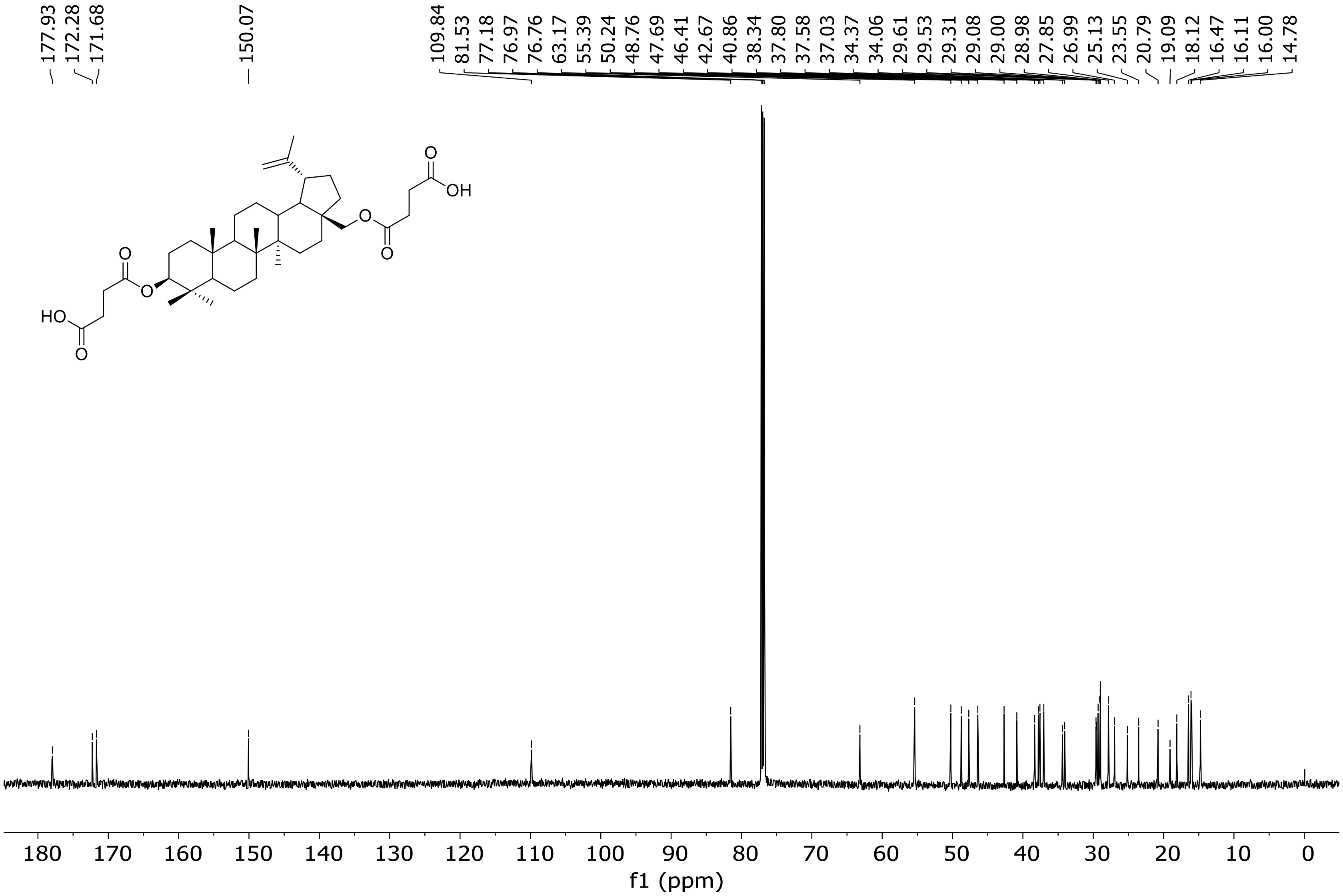

^{13}C NMR spectrum of 3,28-*O,O′*-bis(3′-carboxypropanoyl)betulin (**5**); 150 MHz/$CDCl_3$/TMS; *δ* (ppm).

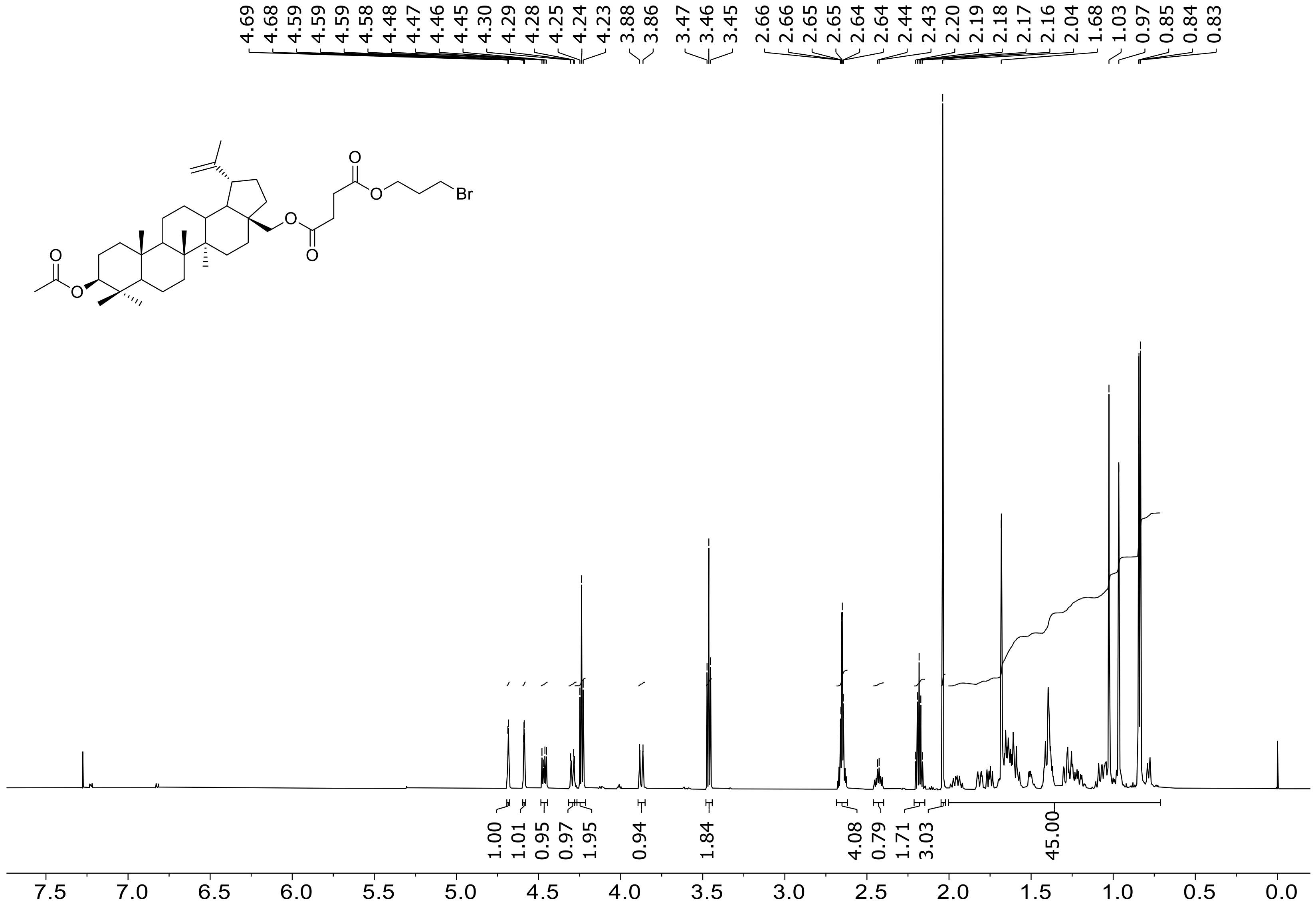

^{1}H NMR spectrum of analogue **6a**; 600 MHz/$CDCl_3$/TMS; δ (ppm).

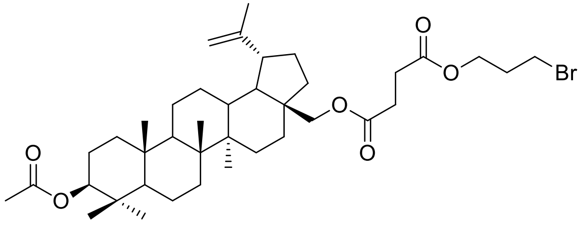

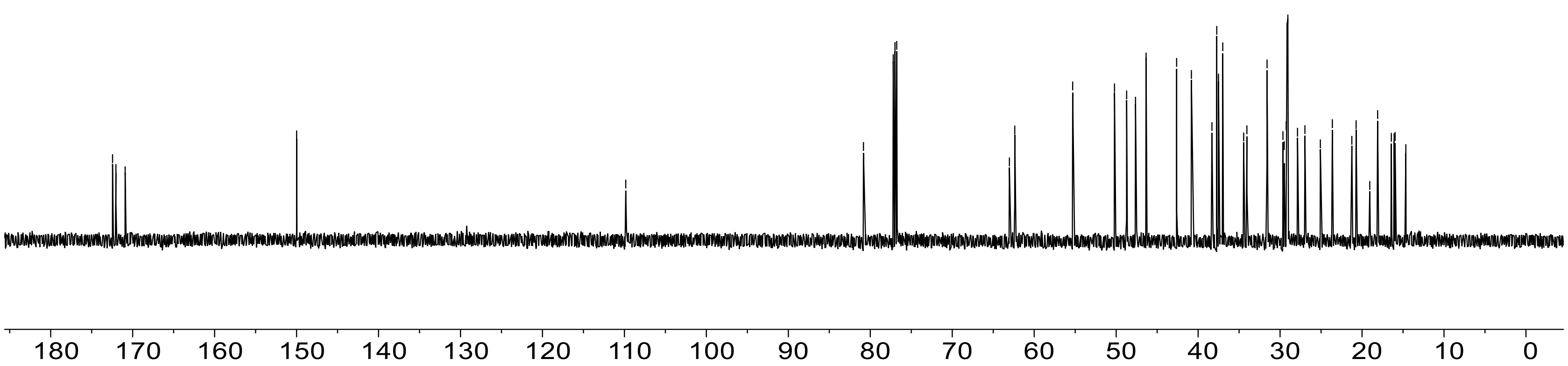

^{13}C NMR spectrum of analogue **6a**; 150 MHz/$CDCl_3$/TMS; δ (ppm).

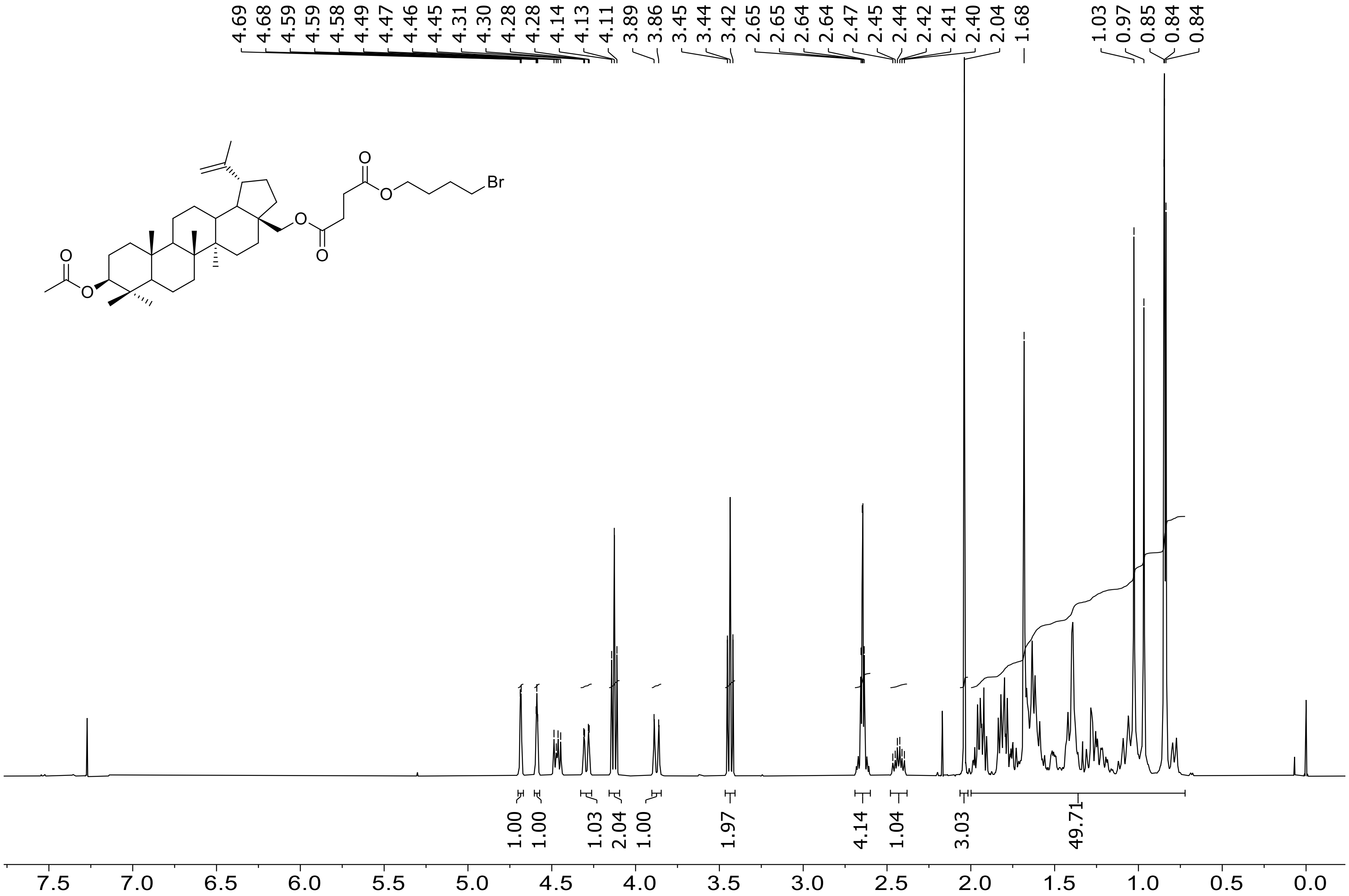

^{1}H NMR spectrum of analogue **6b**; 400 MHz/$CDCl_3$/TMS; δ (ppm).

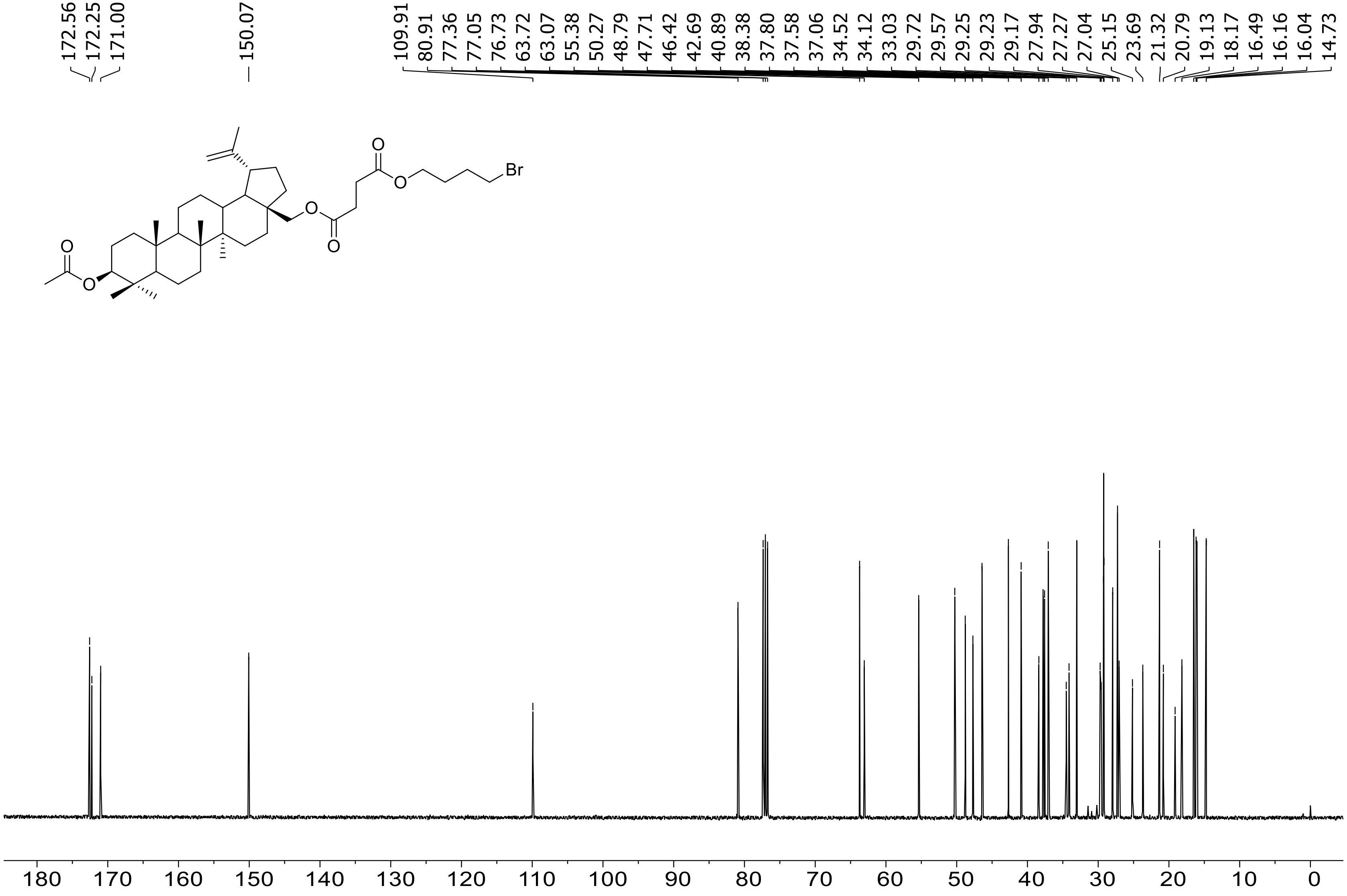

^{13}C NMR spectrum of analogue **6b**; 100 MHz/$CDCl_3$/TMS; *δ* (ppm).

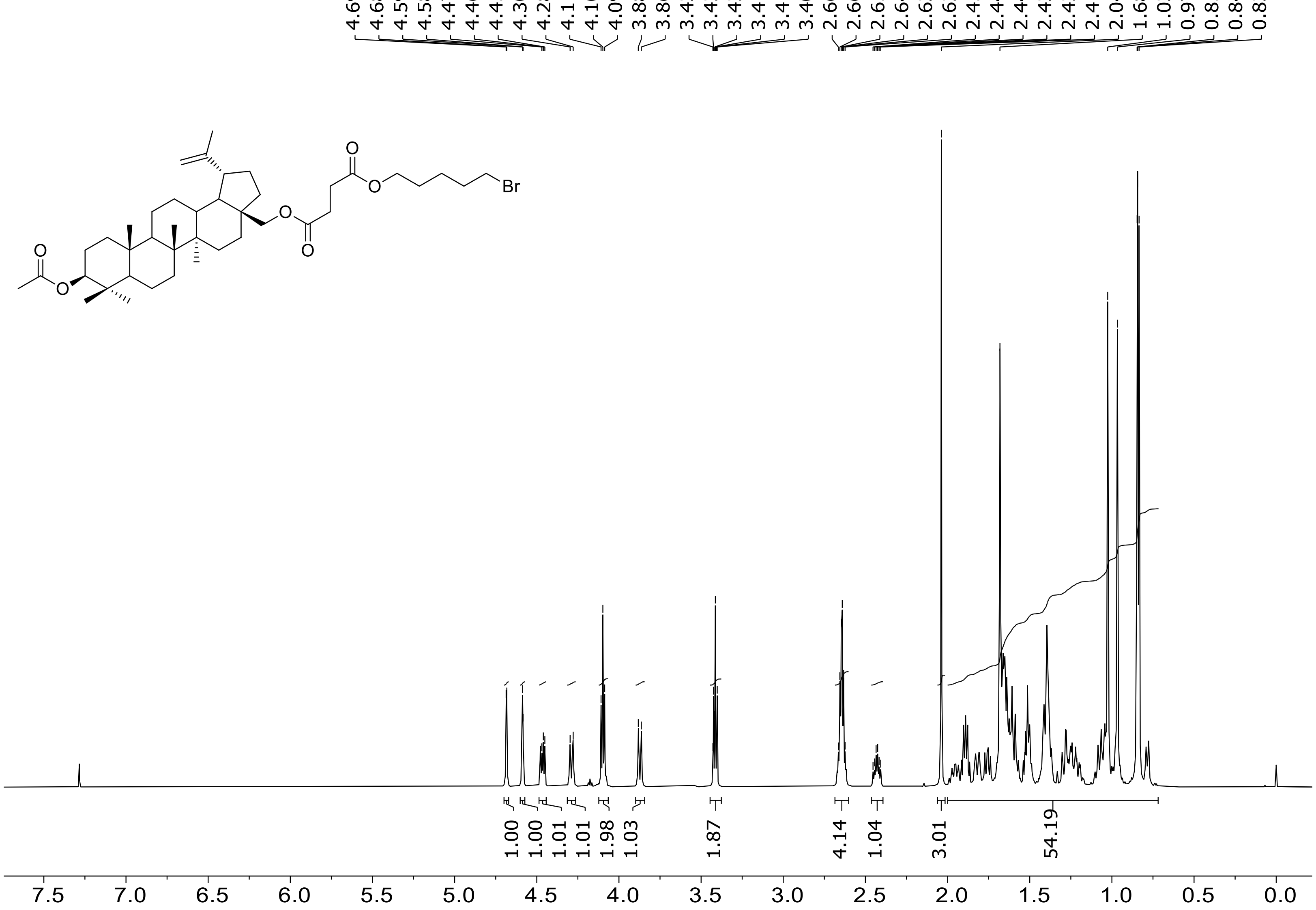

^{1}H NMR spectrum of analogue **6c**; 600 MHz/$CDCl_3$/TMS; δ (ppm).

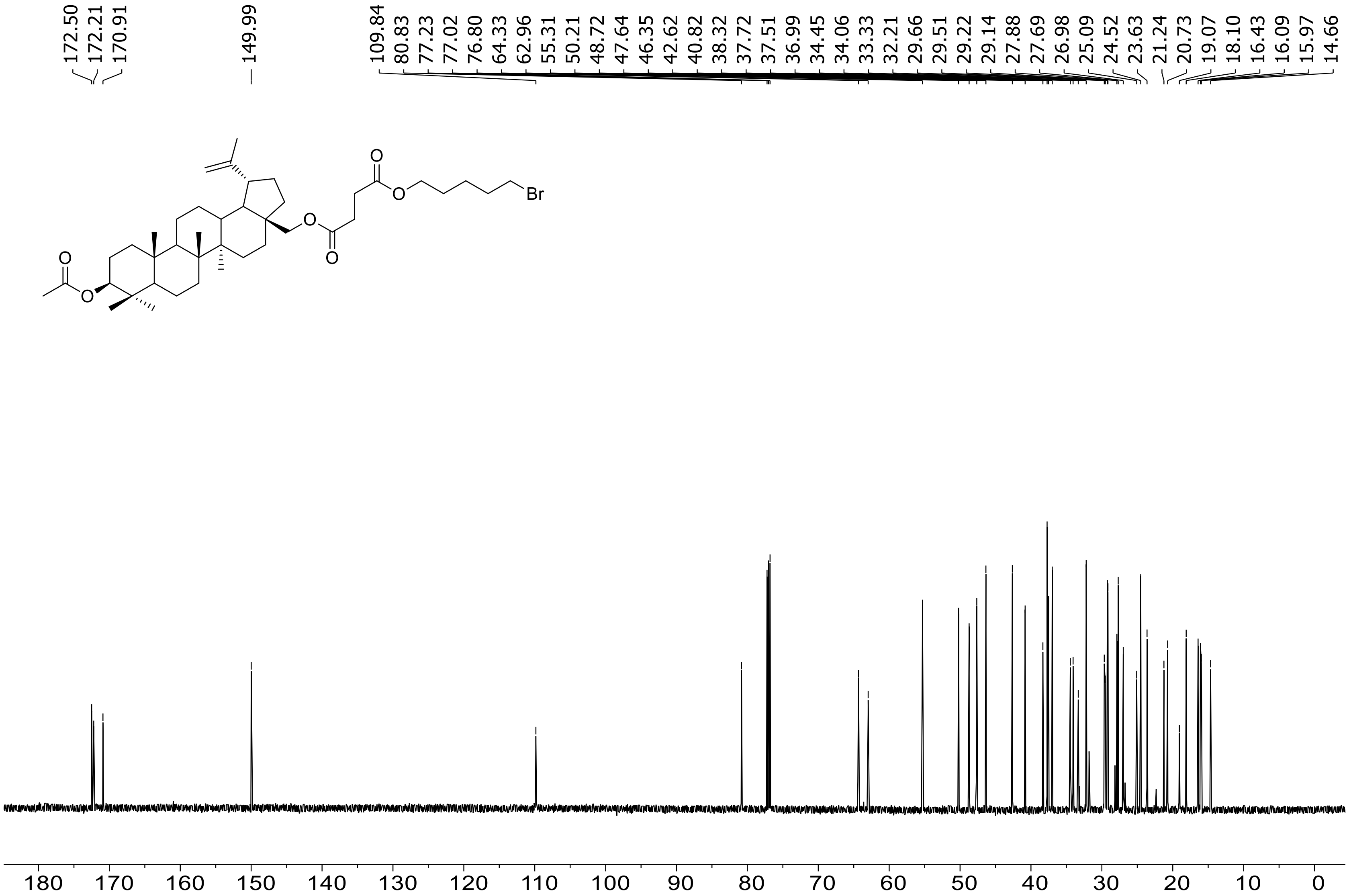

^{13}C NMR spectrum of analogue **6c**; 150 MHz/$CDCl_3$/TMS; δ (ppm).

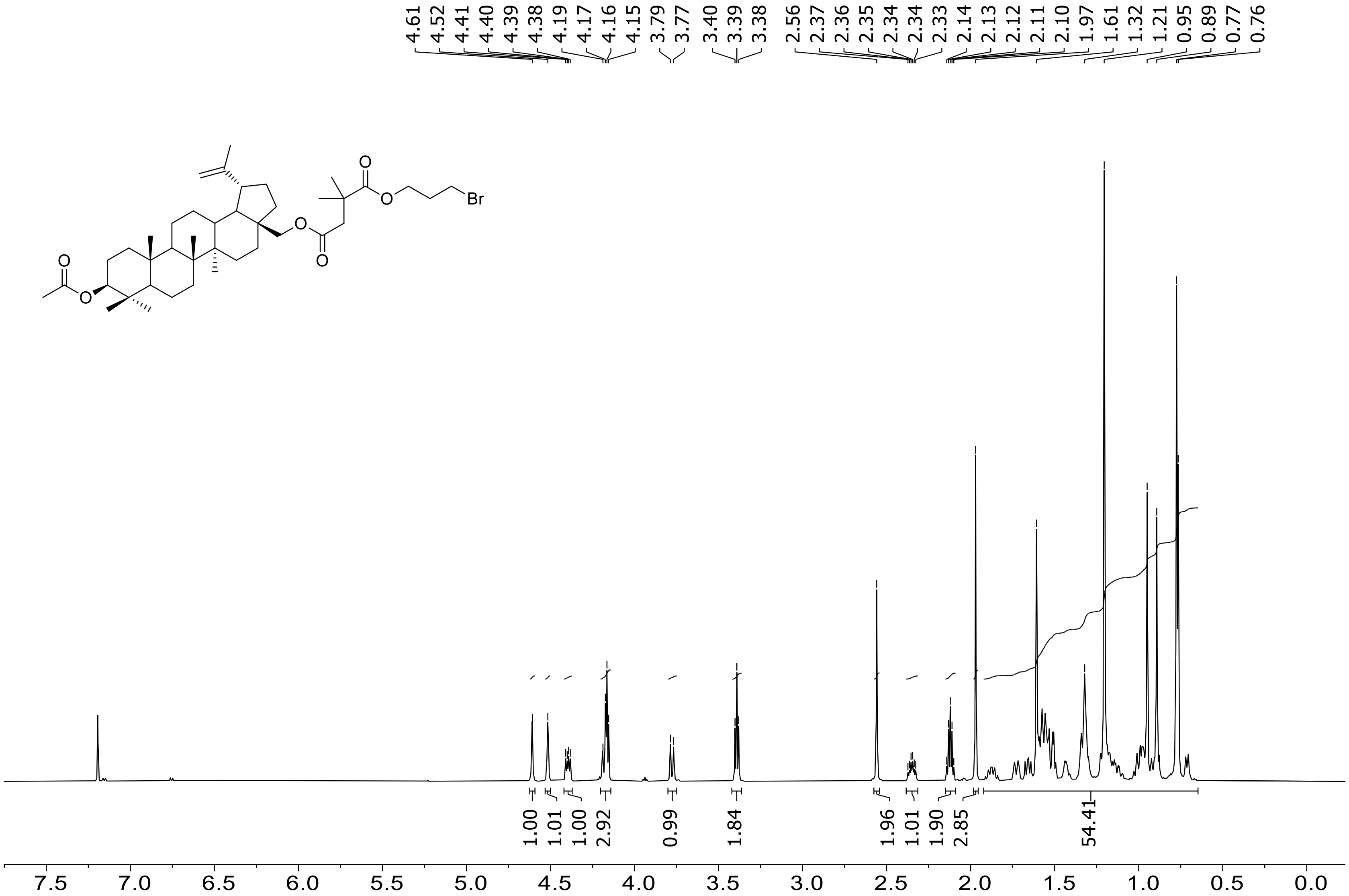

^{1}H NMR spectrum of analogue **6d**; 400 MHz/$CDCl_3$/TMS; δ (ppm).

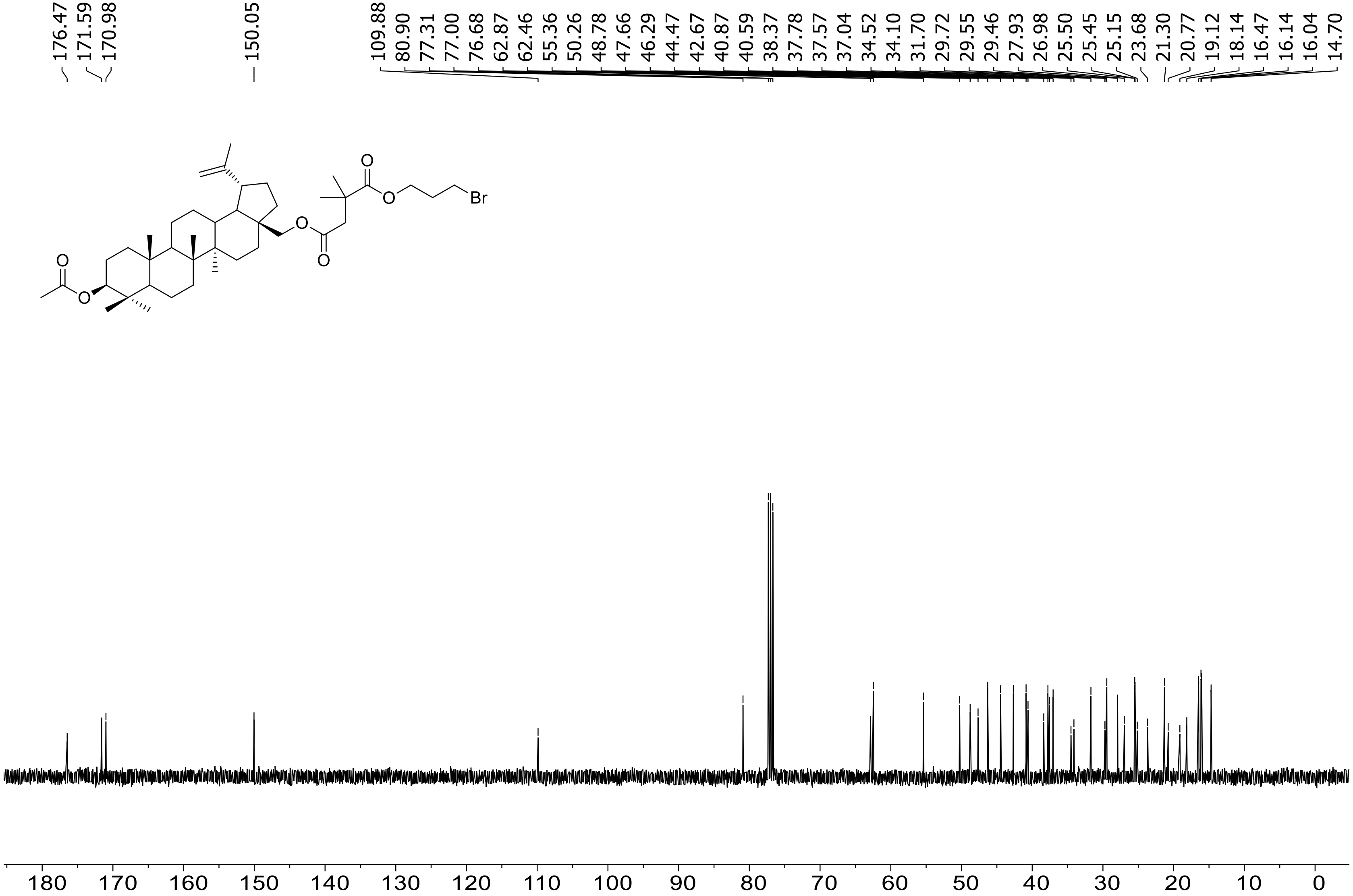

^{13}C NMR spectrum of analogue **6d**; 100 MHz/$CDCl_3$/TMS; δ (ppm).

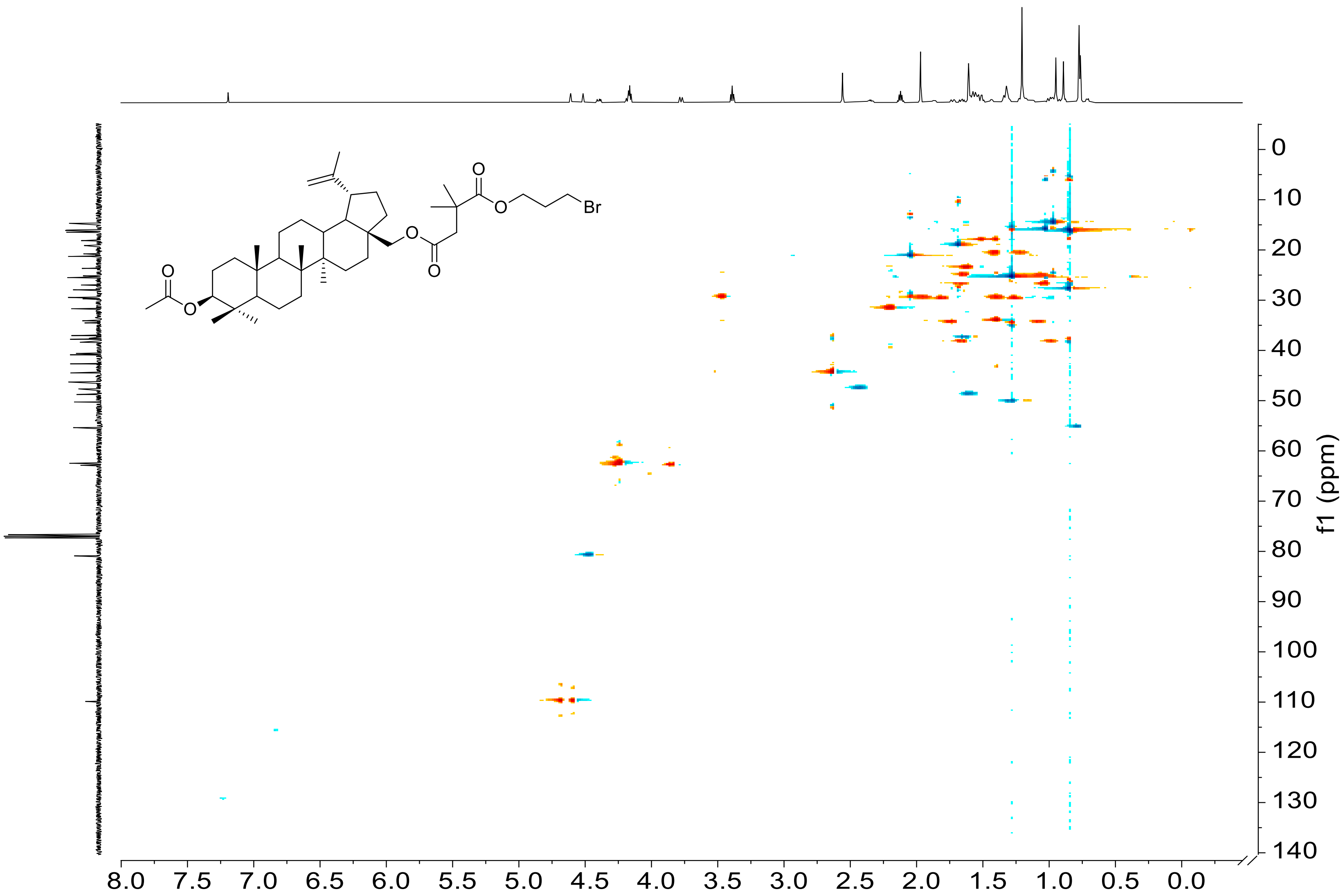

gHSQC spectrum of analogue **6d**

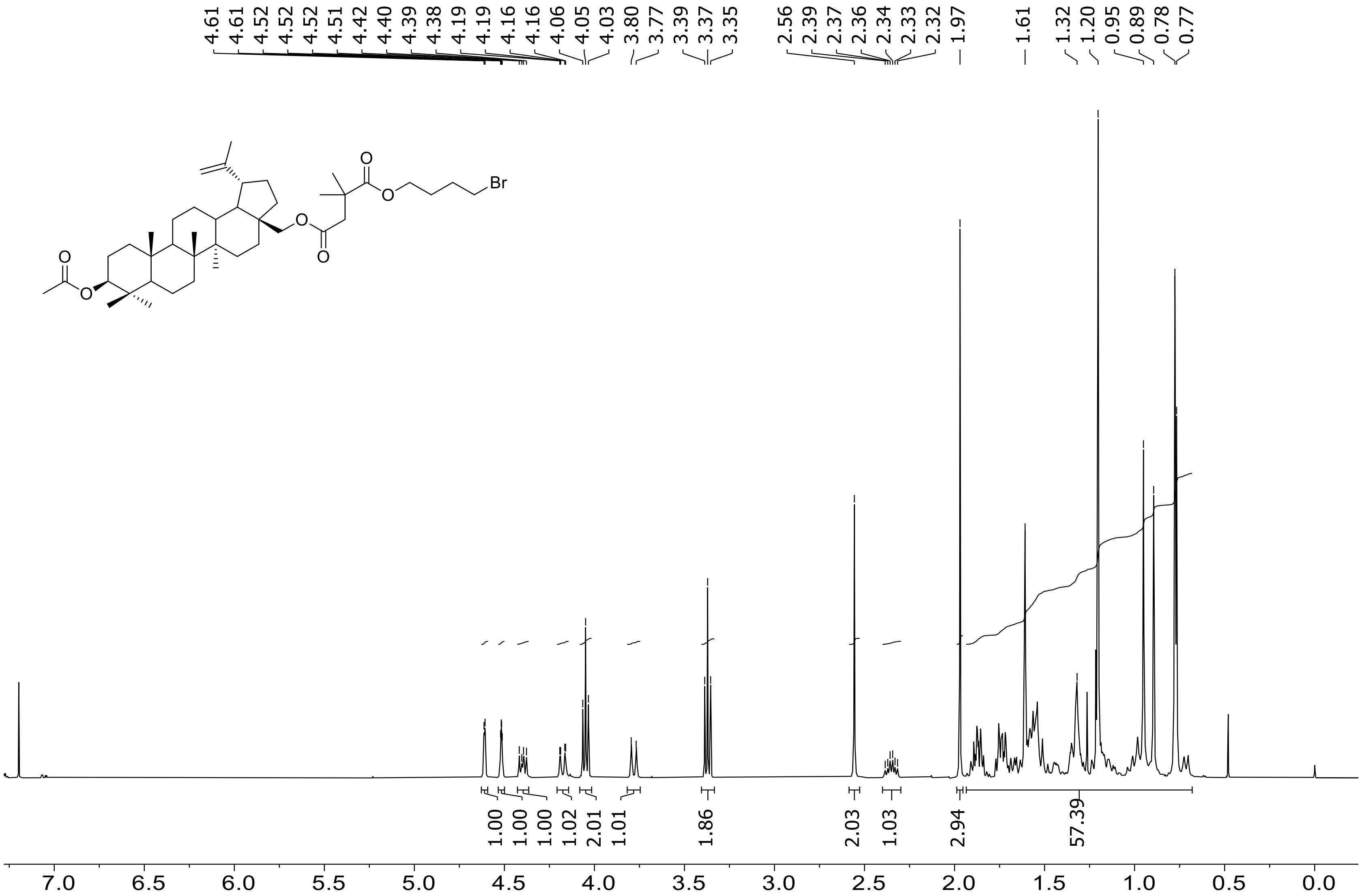

^{1}H NMR spectrum of analogue **6e**; 400 MHz/$CDCl_3$/TMS; δ (ppm).

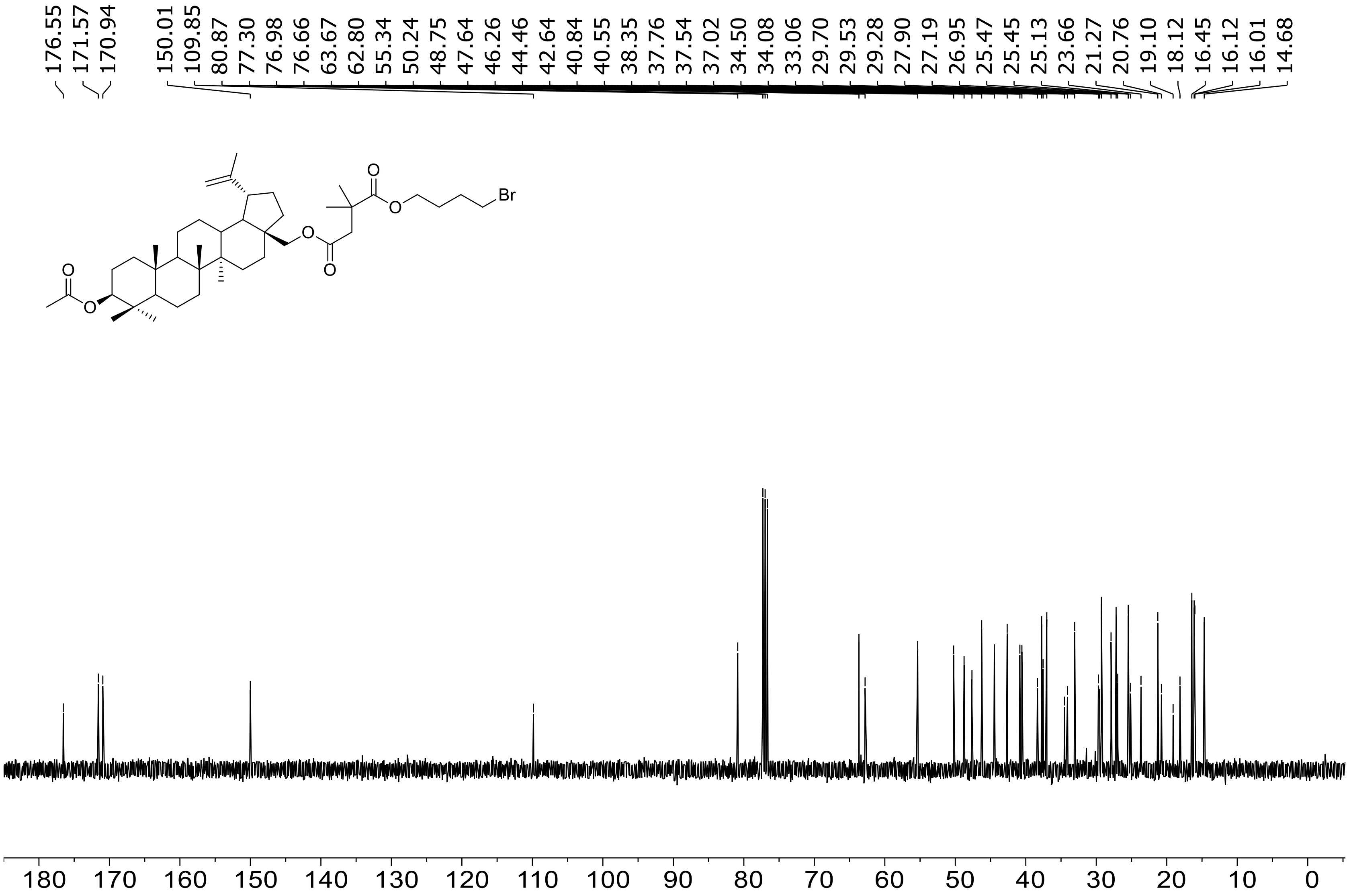

^{13}C NMR spectrum of analogue **6e**; 100 MHz/CDCl$_3$/TMS; δ (ppm).

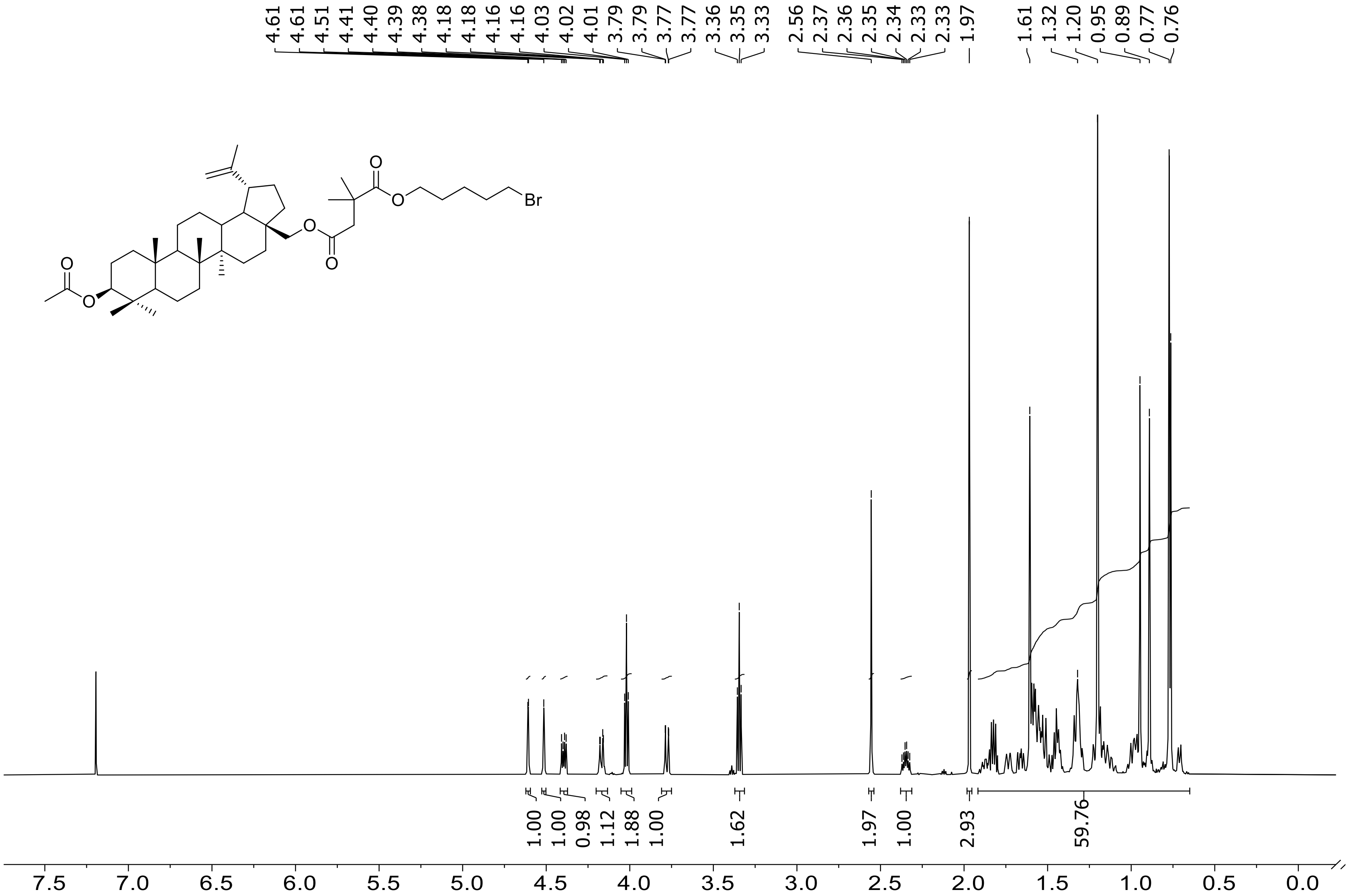

^{1}H NMR spectrum of analogue **6f**; 600 MHz/$CDCl_3$/TMS; δ (ppm).

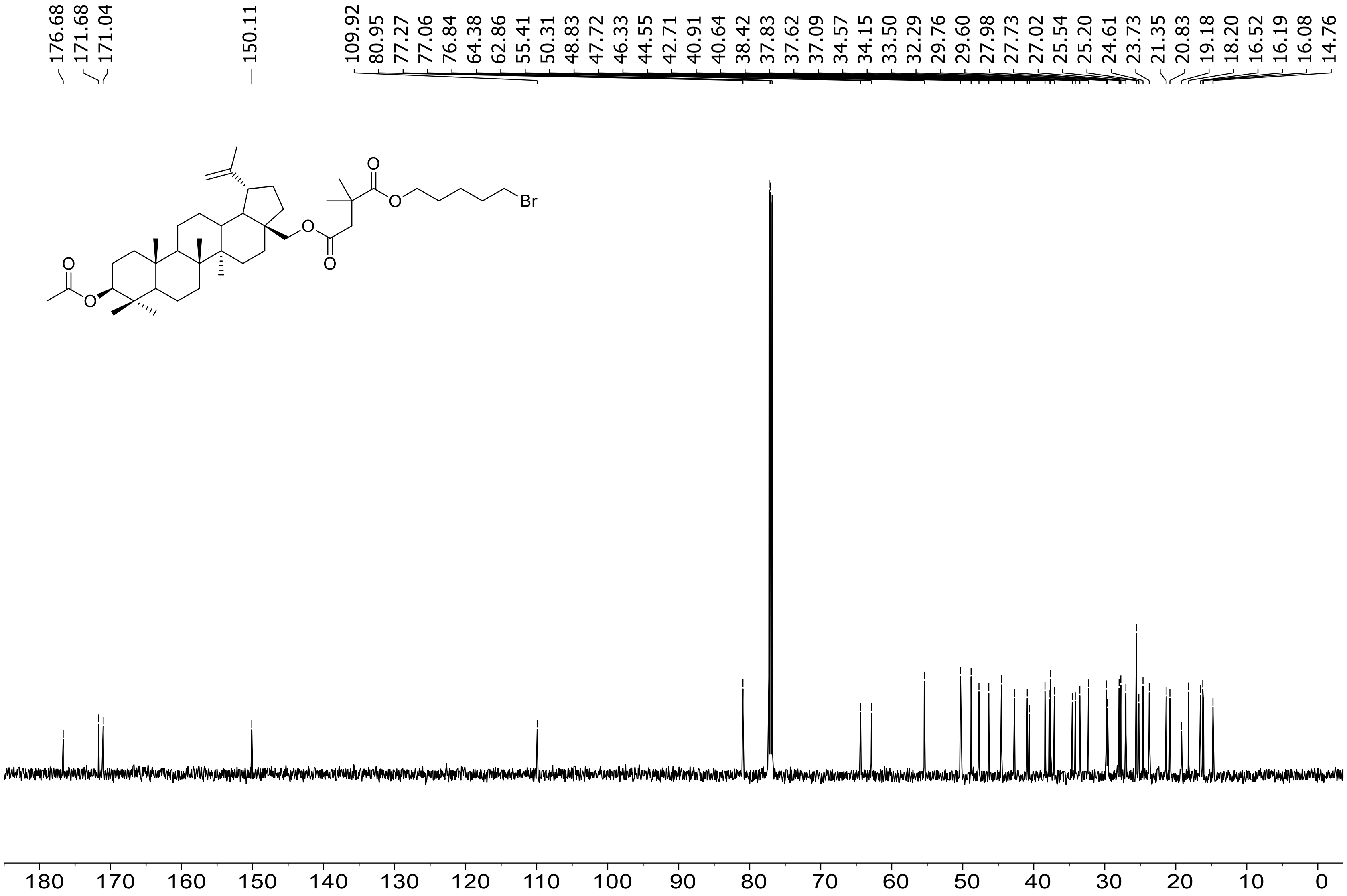

^{13}C NMR spectrum of analogue **6f**; 150 MHz/CDCl$_3$/TMS; δ (ppm).

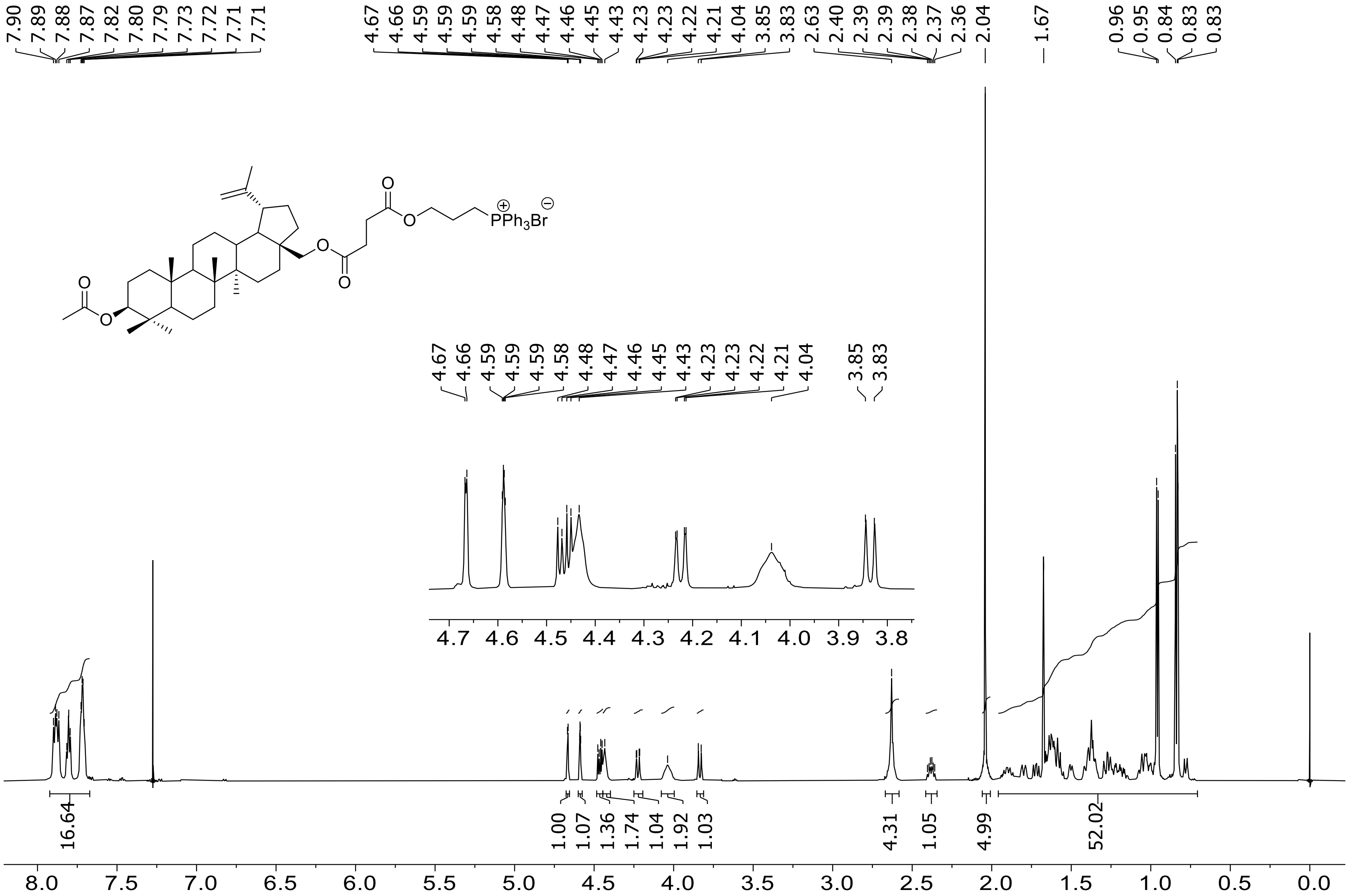

^{1}H NMR spectrum of analogue **7a**; 600 MHz/$CDCl_3$/TMS; δ (ppm).

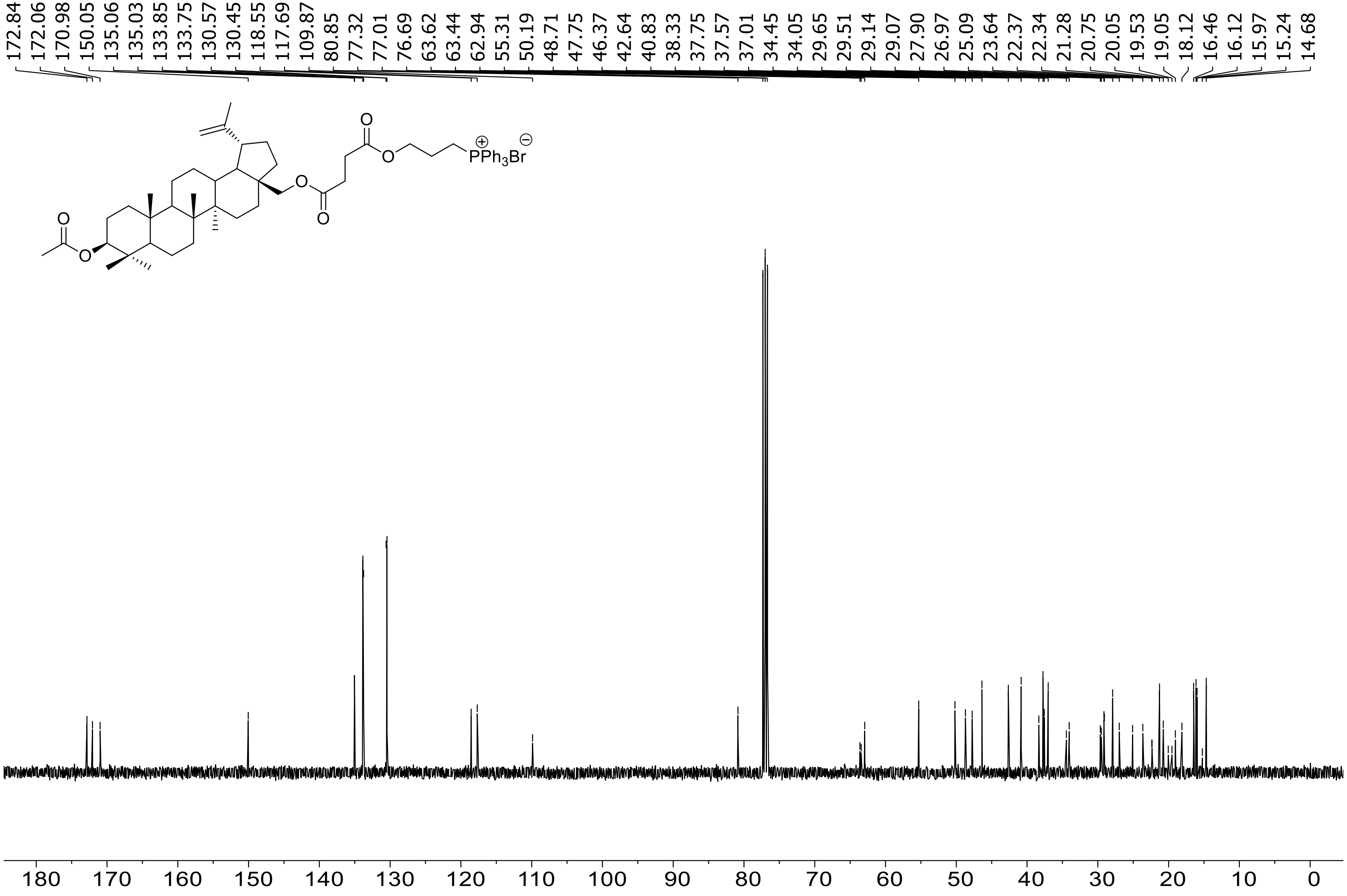

^{13}C NMR spectrum of analogue **7a**; 100 MHz/$CDCl_3$/TMS; δ (ppm).

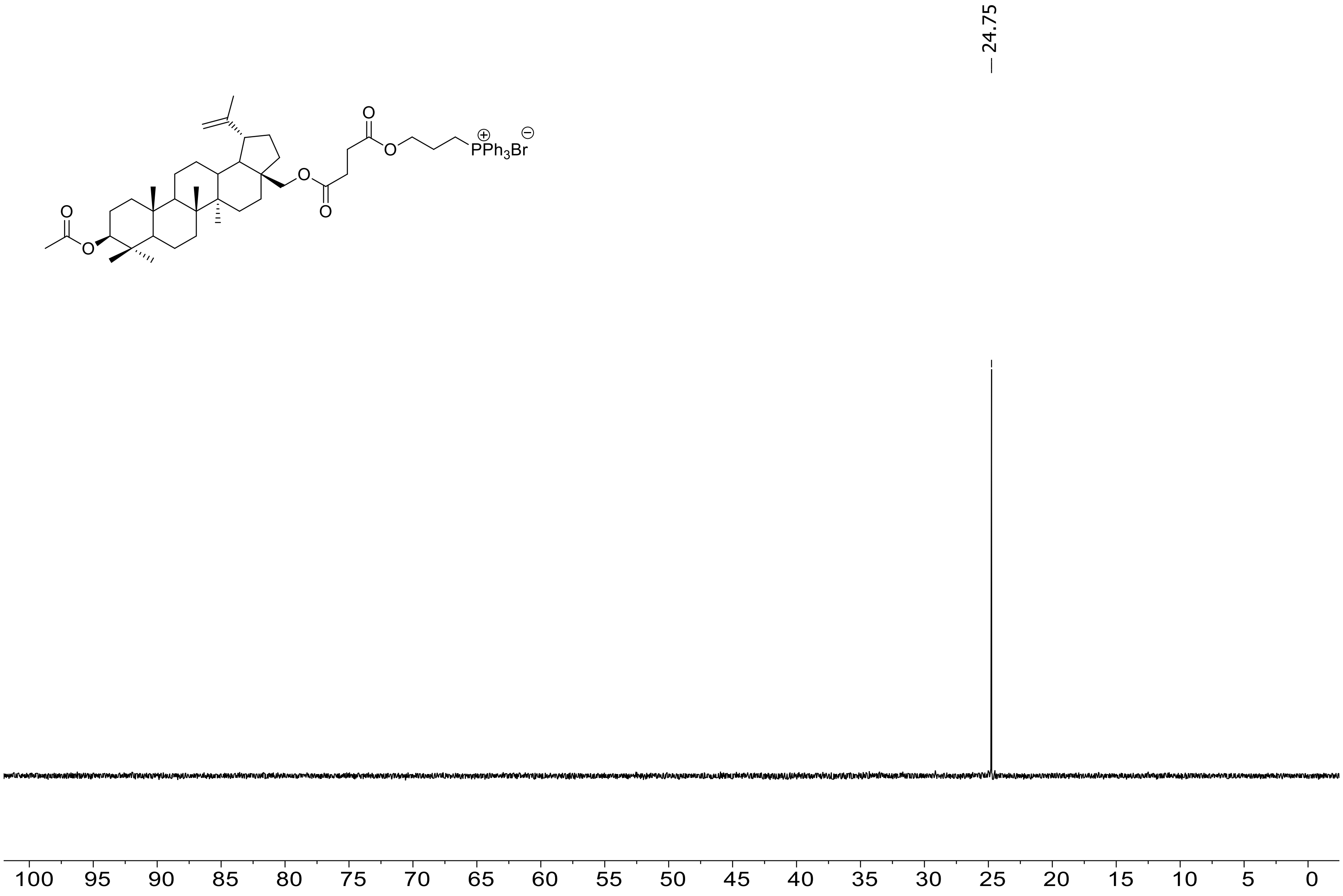

^{31}P NMR spectrum of analogue **7a**; 161.9 MHz/$CDCl_3$/TMS; δ (ppm).

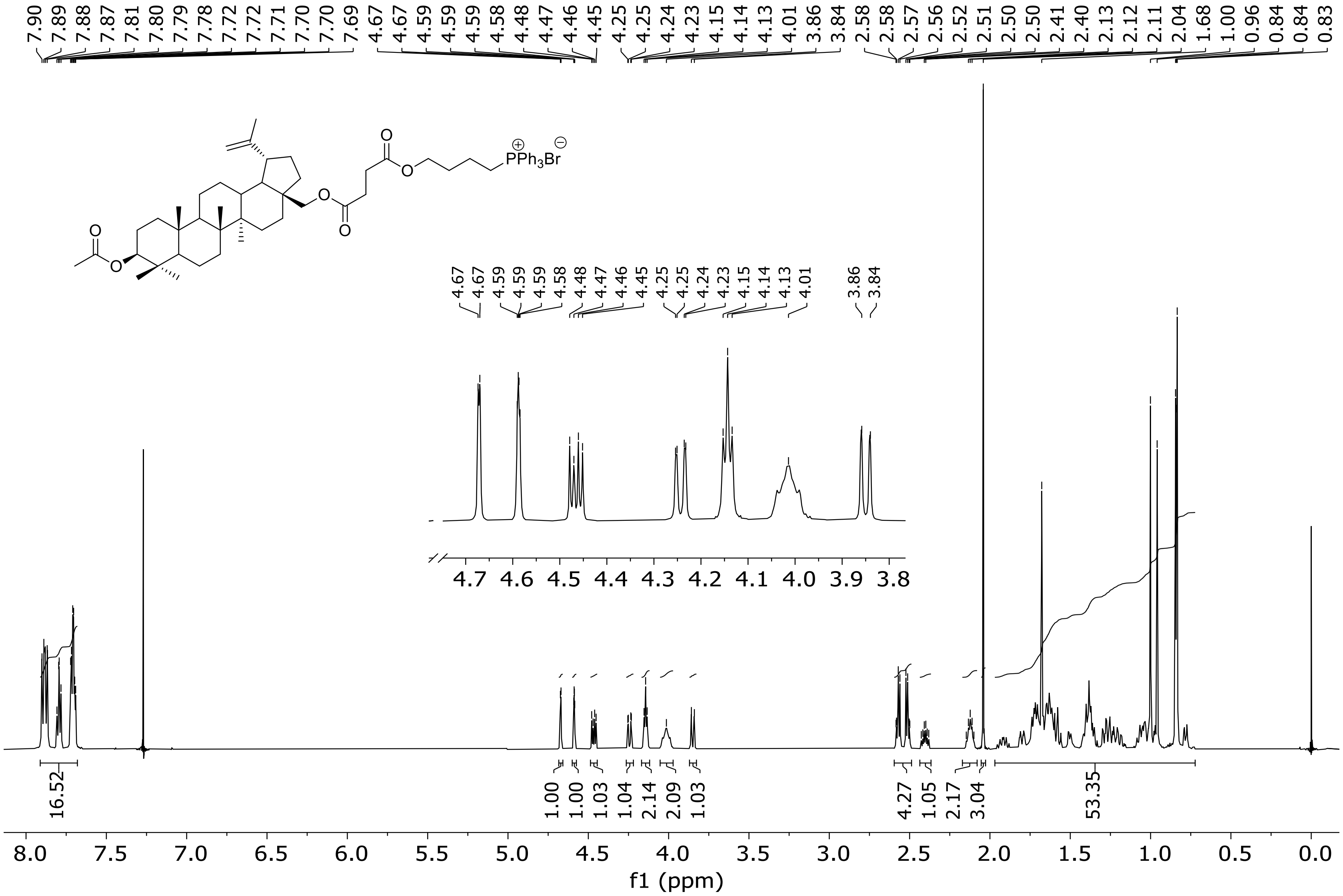

^{1}H NMR spectrum of analogue **7b**; 600 MHz/$CDCl_3$/TMS; δ (ppm).

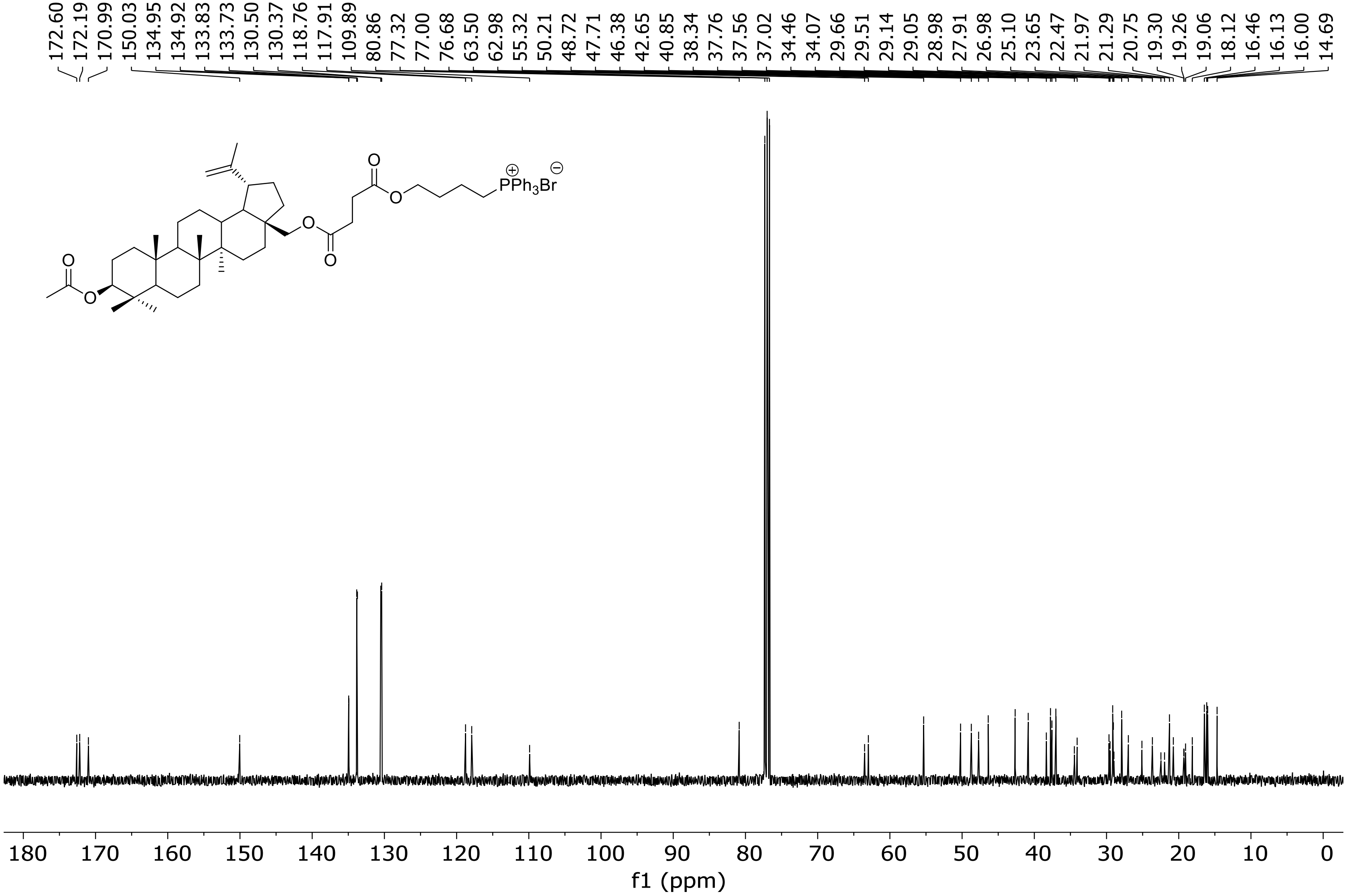

^{13}C NMR spectrum of analogue **7b**; 100 MHz/$CDCl_3$/TMS; δ (ppm).

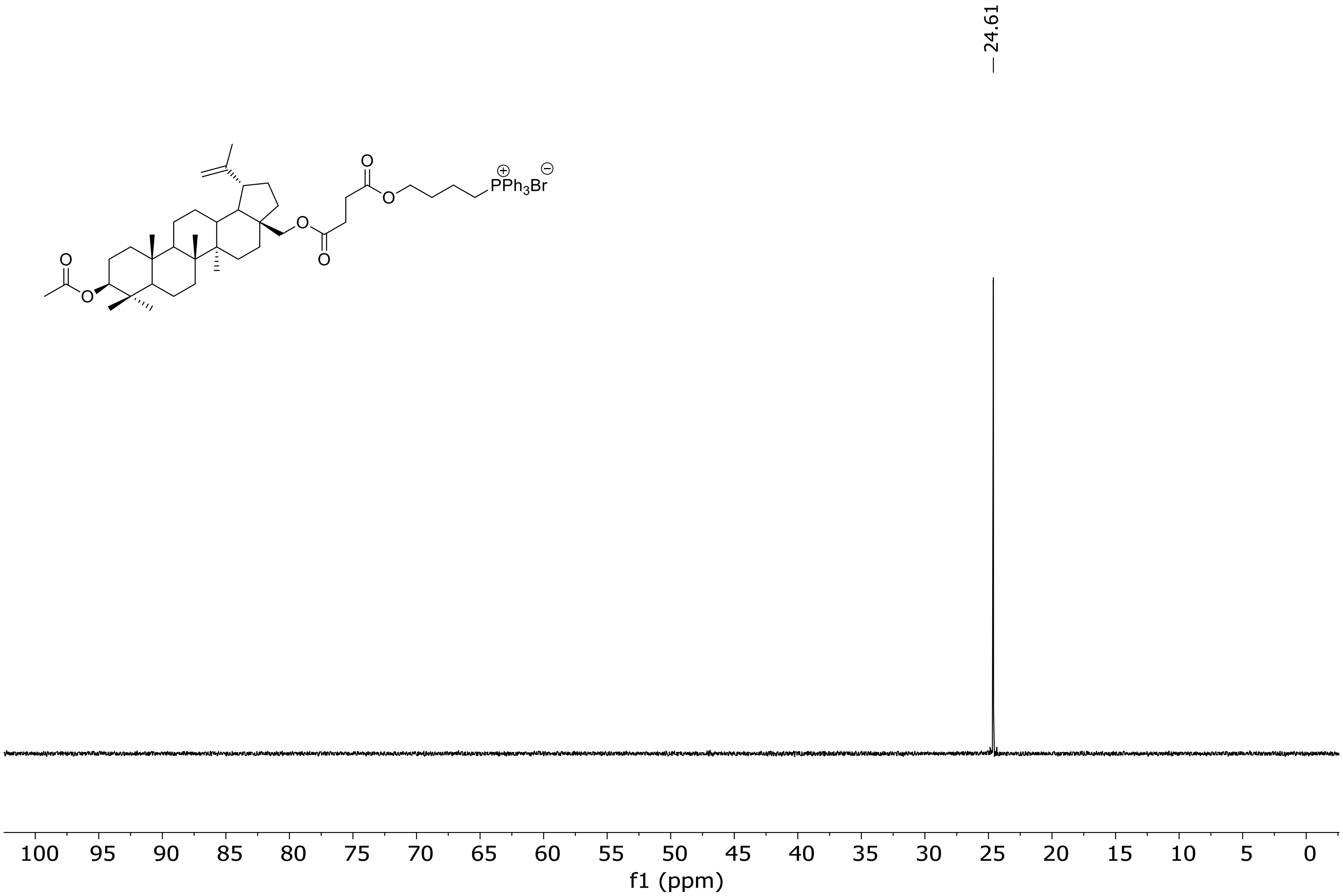

^{31}P NMR spectrum of analogue **7b**; 161.9 MHz/$CDCl_3$/TMS; δ (ppm).

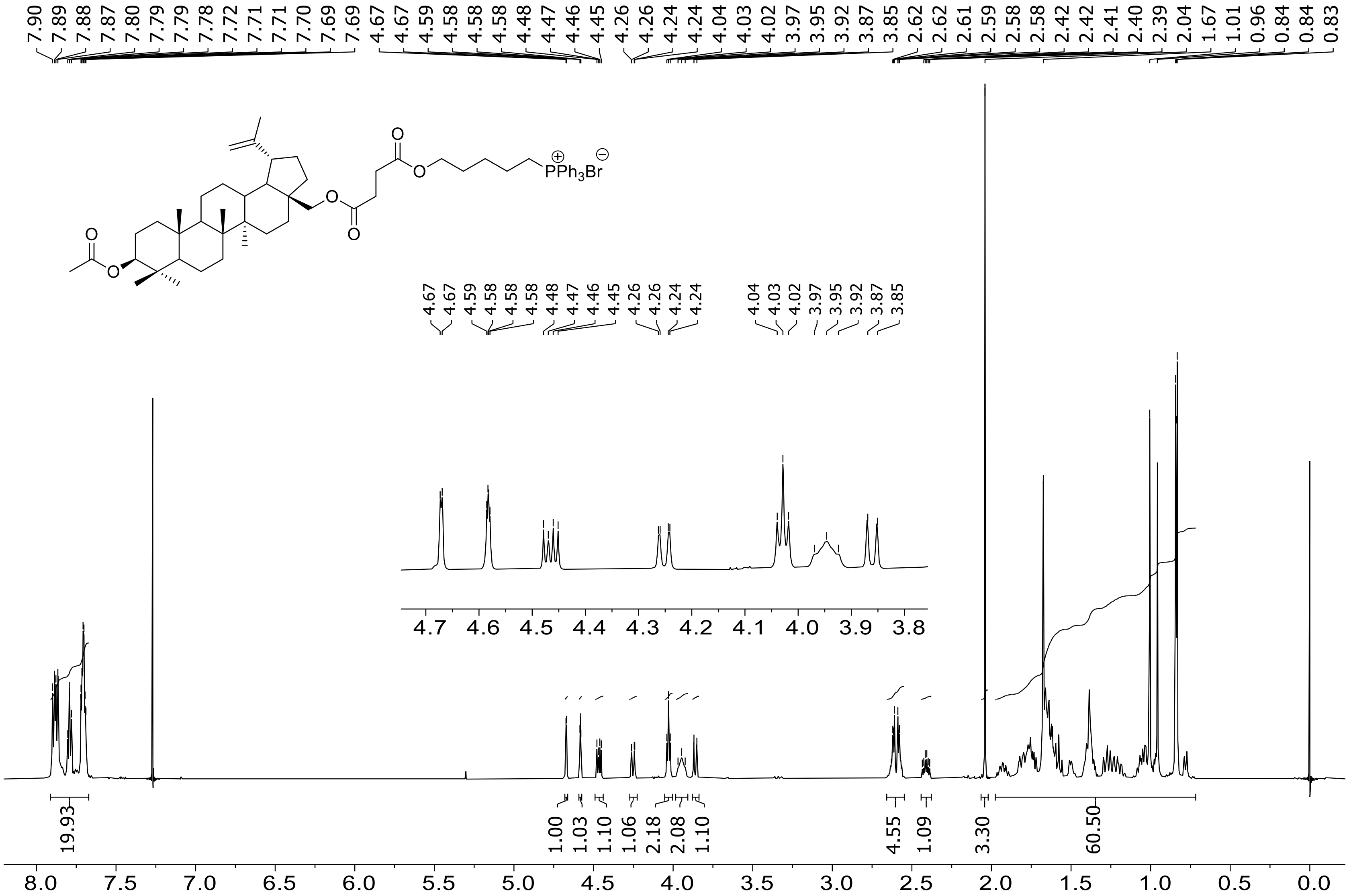

^{1}H NMR spectrum of analogue **7c**; 600 MHz/$CDCl_3$/TMS; δ (ppm).

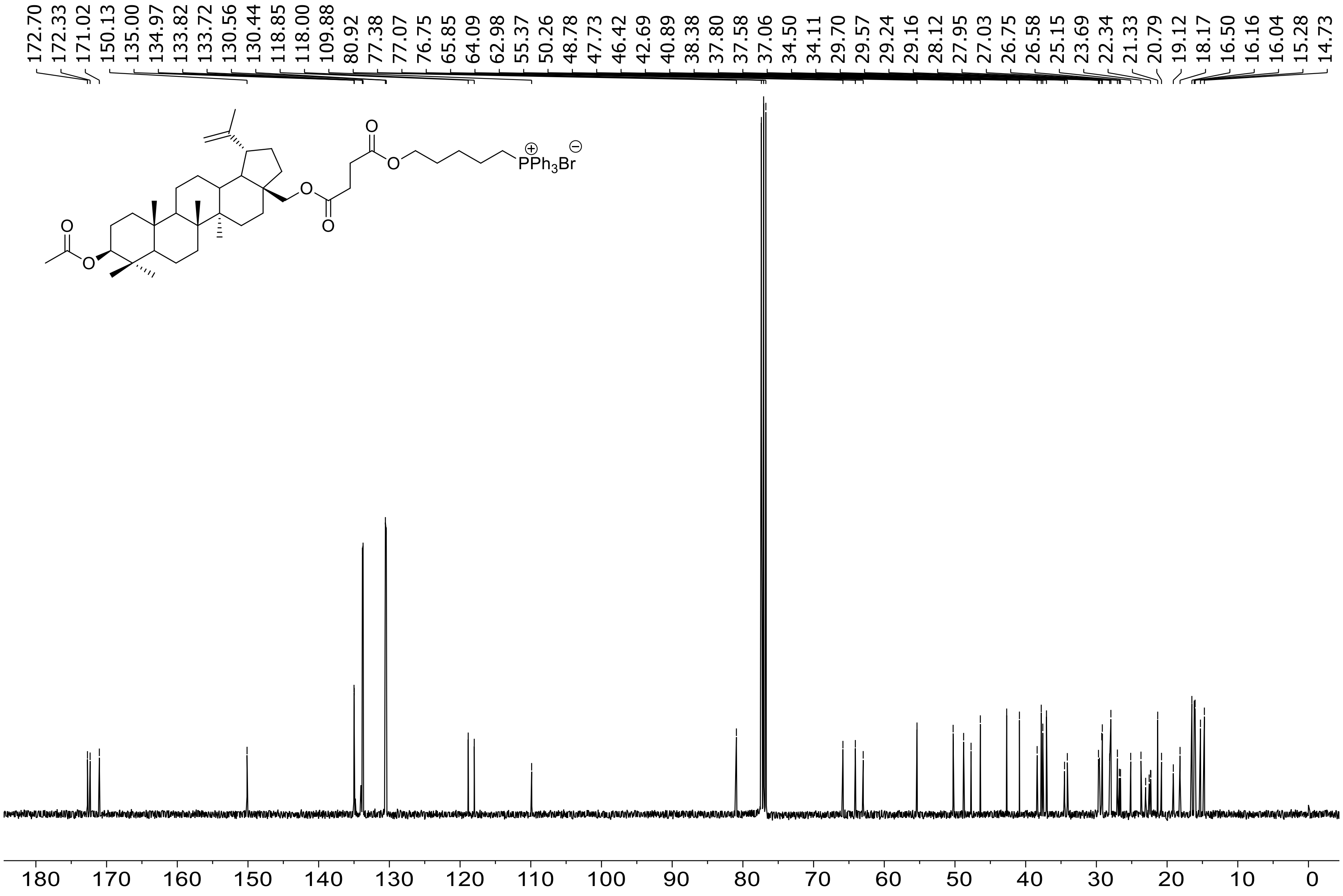

^{13}C NMR spectrum of analogue **7c**; 100 MHz/$CDCl_3$/TMS; δ (ppm).

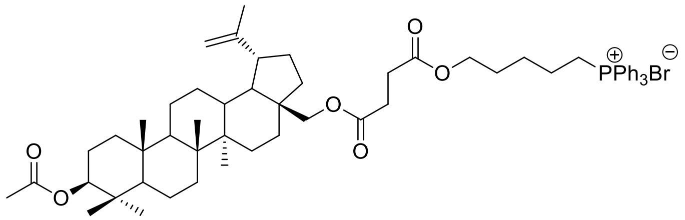

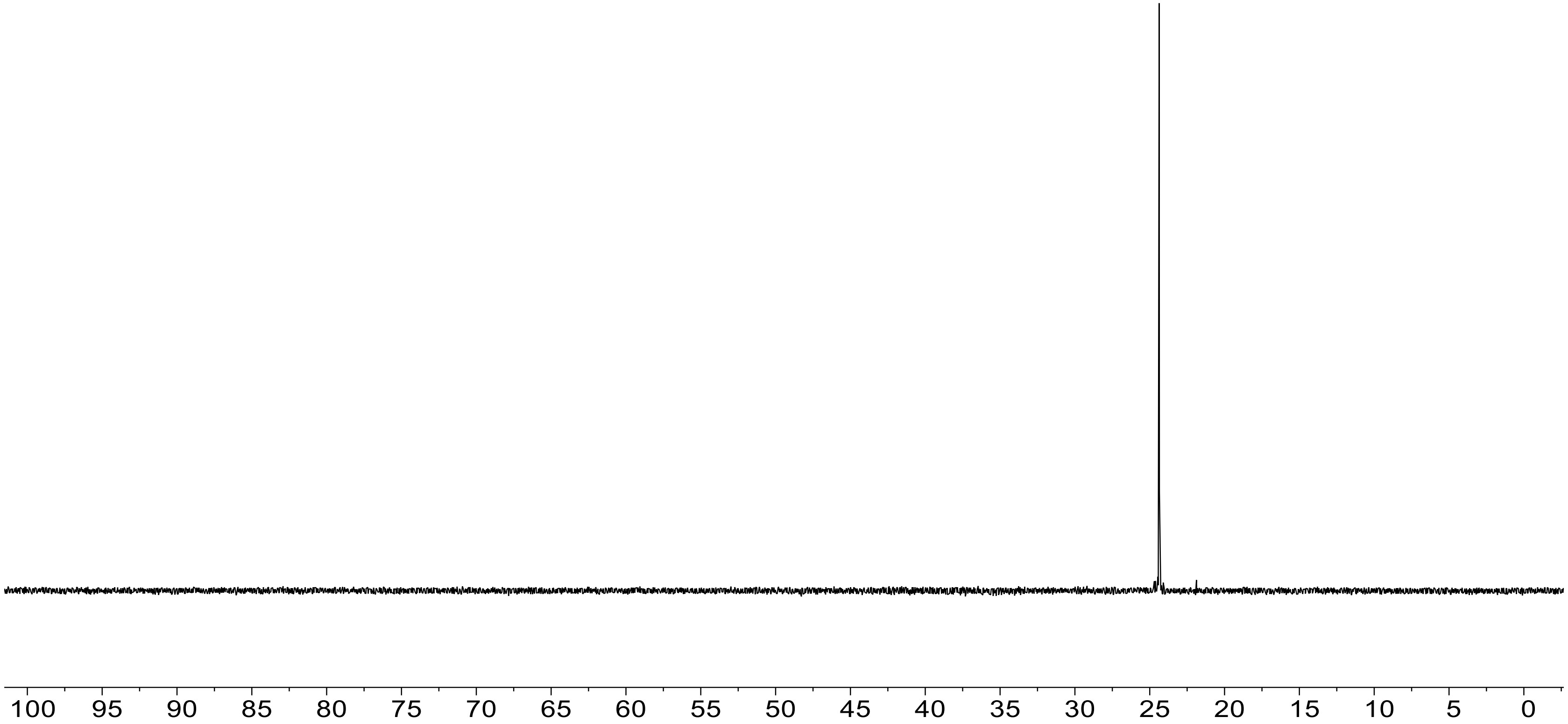

^{31}P NMR spectrum of analogue **7c**; 161.9 MHz/$CDCl_3$/TMS; δ (ppm).

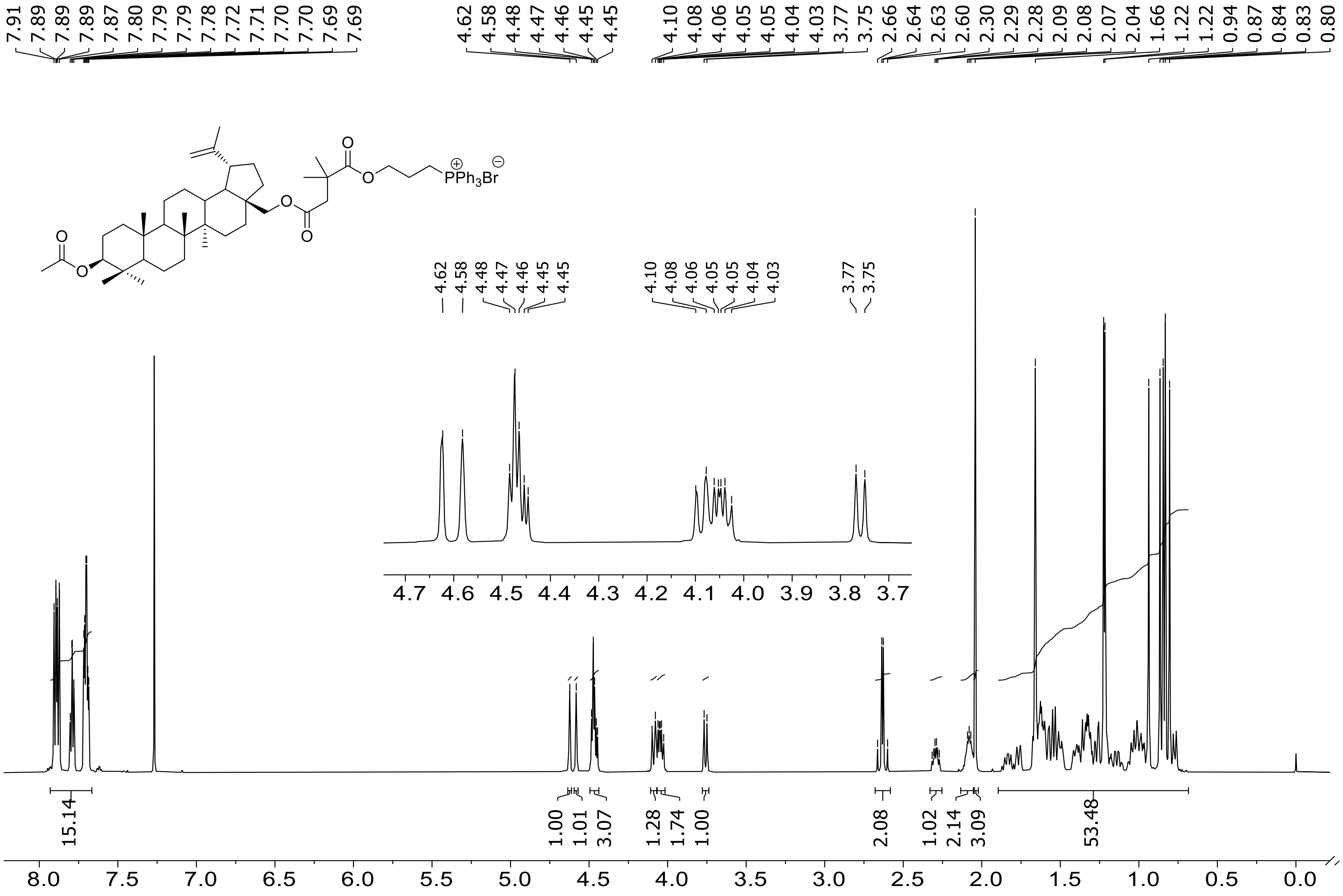

^{1}H NMR spectrum of analogue **7d**; 600 MHz/$CDCl_3$/TMS; δ (ppm).

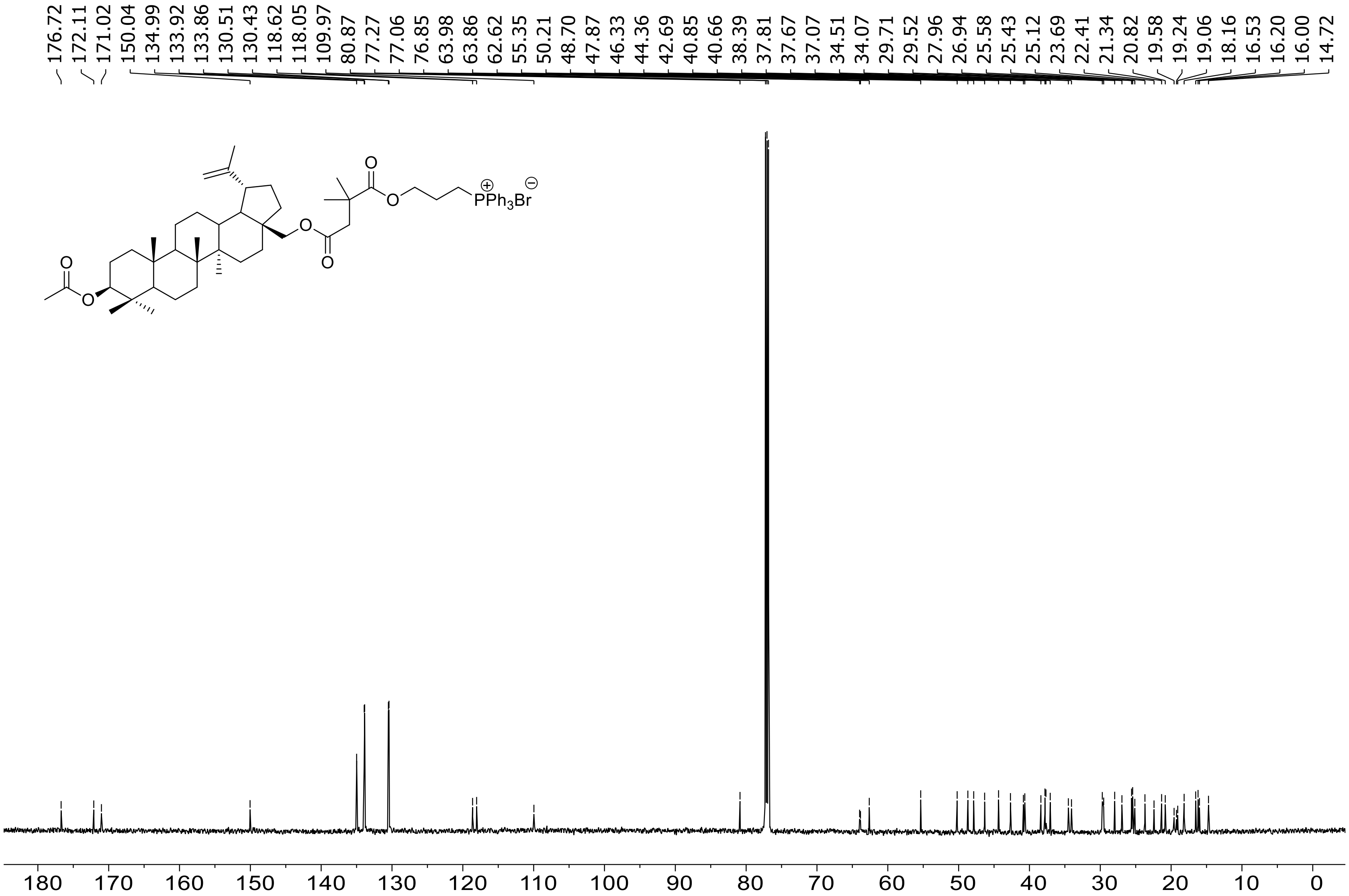

^{13}C NMR spectrum of analogue **7d**; 150 MHz/$CDCl_3$/TMS; *δ* (ppm).

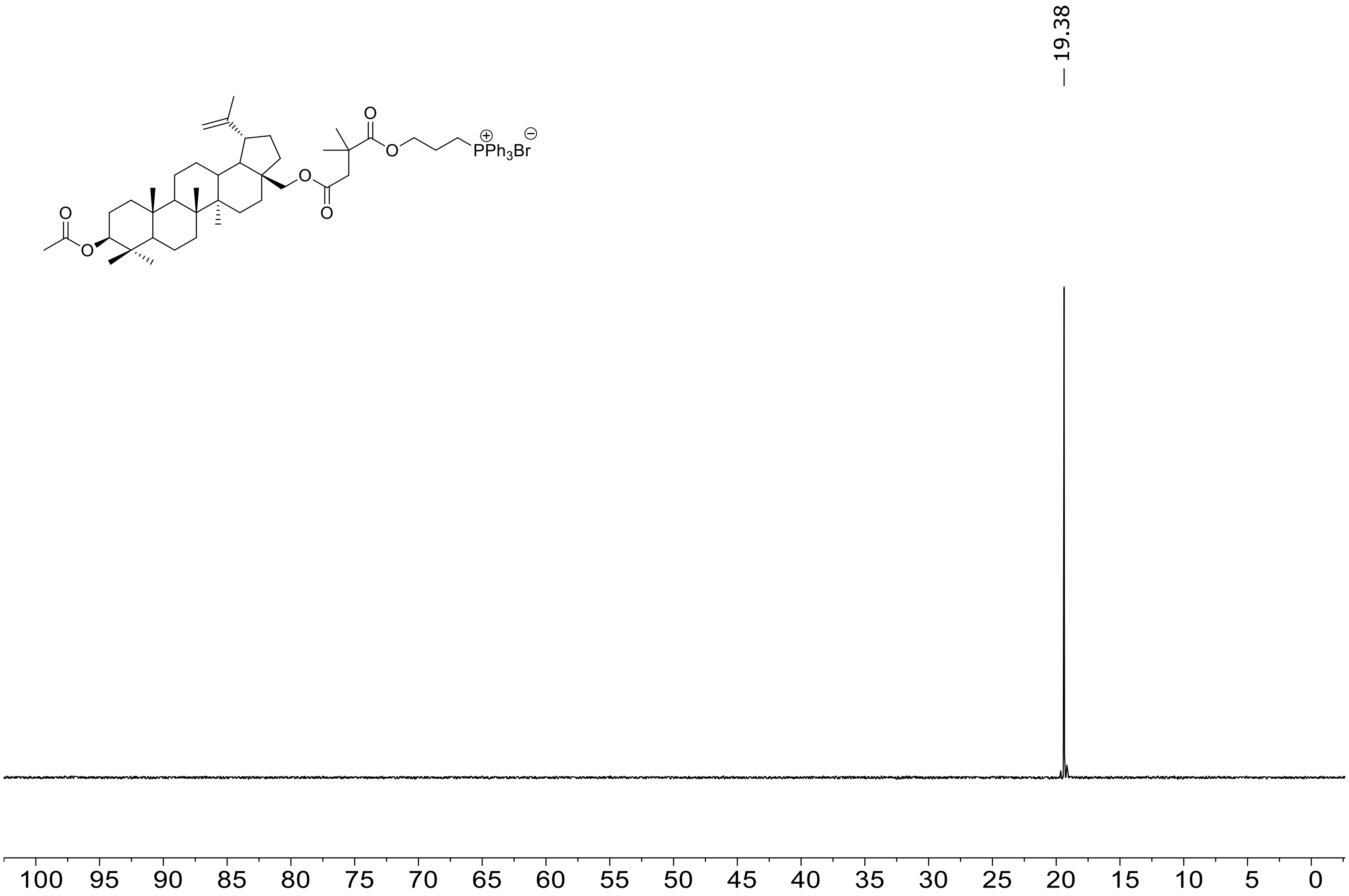

^{31}P NMR spectrum of analogue **7d**; 161.9 MHz/CDCl$_3$/TMS; δ (ppm).

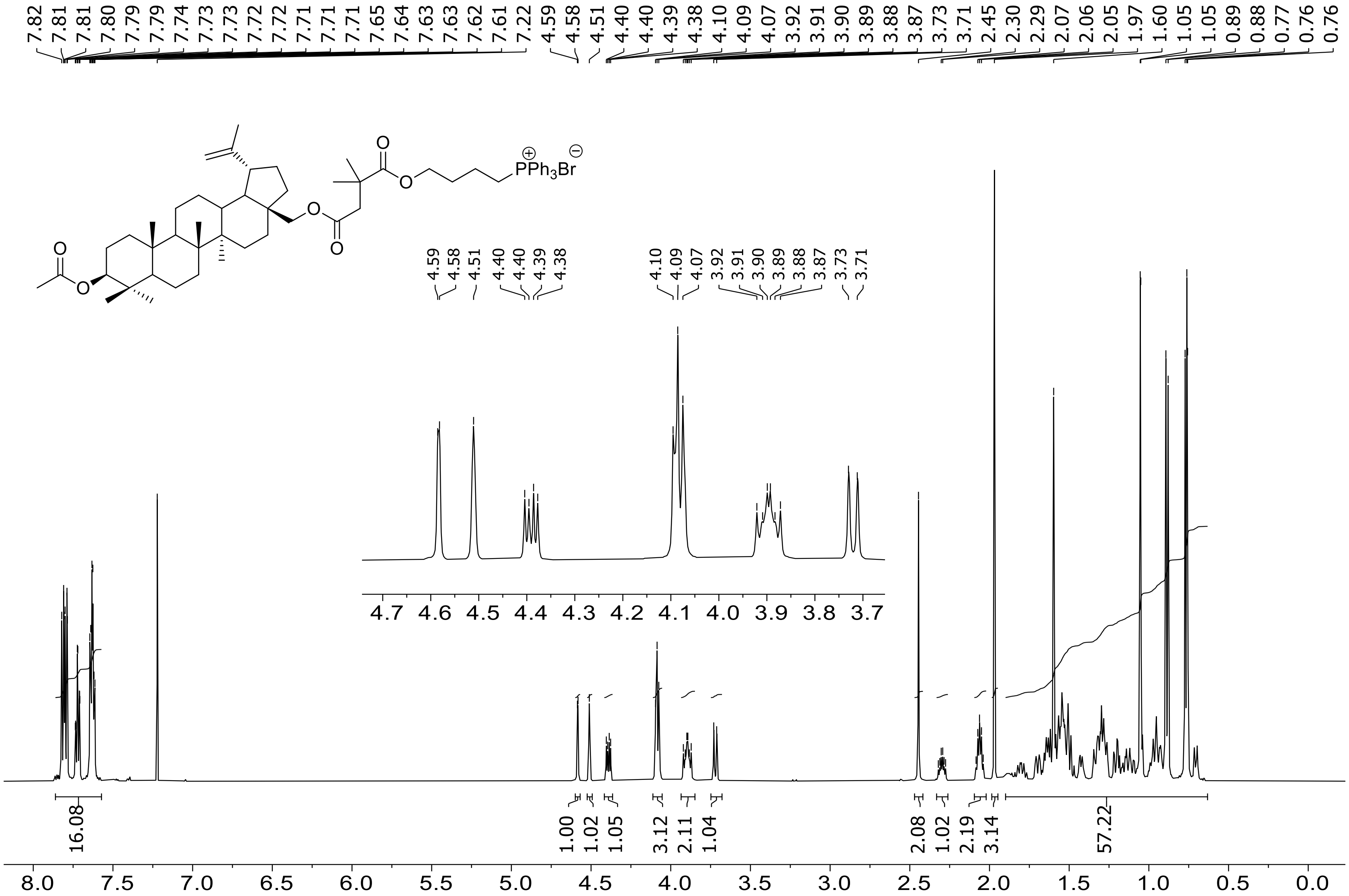

^{1}H NMR spectrum of analogue **7e**; 600 MHz/$CDCl_3$/TMS; δ (ppm).

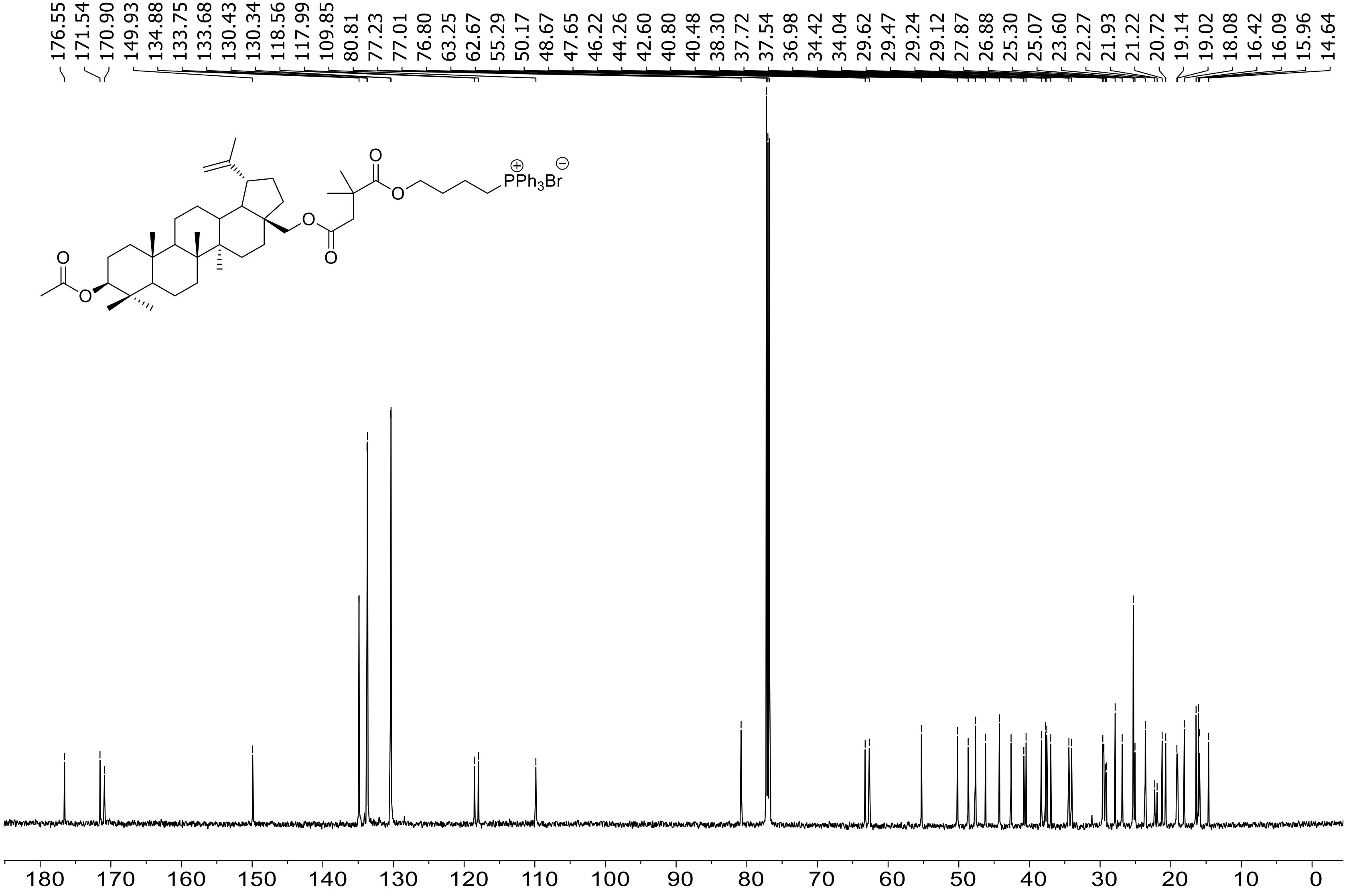

^{13}C NMR spectrum of analogue **7e**; 150 MHz/$CDCl_3$/TMS; δ (ppm).

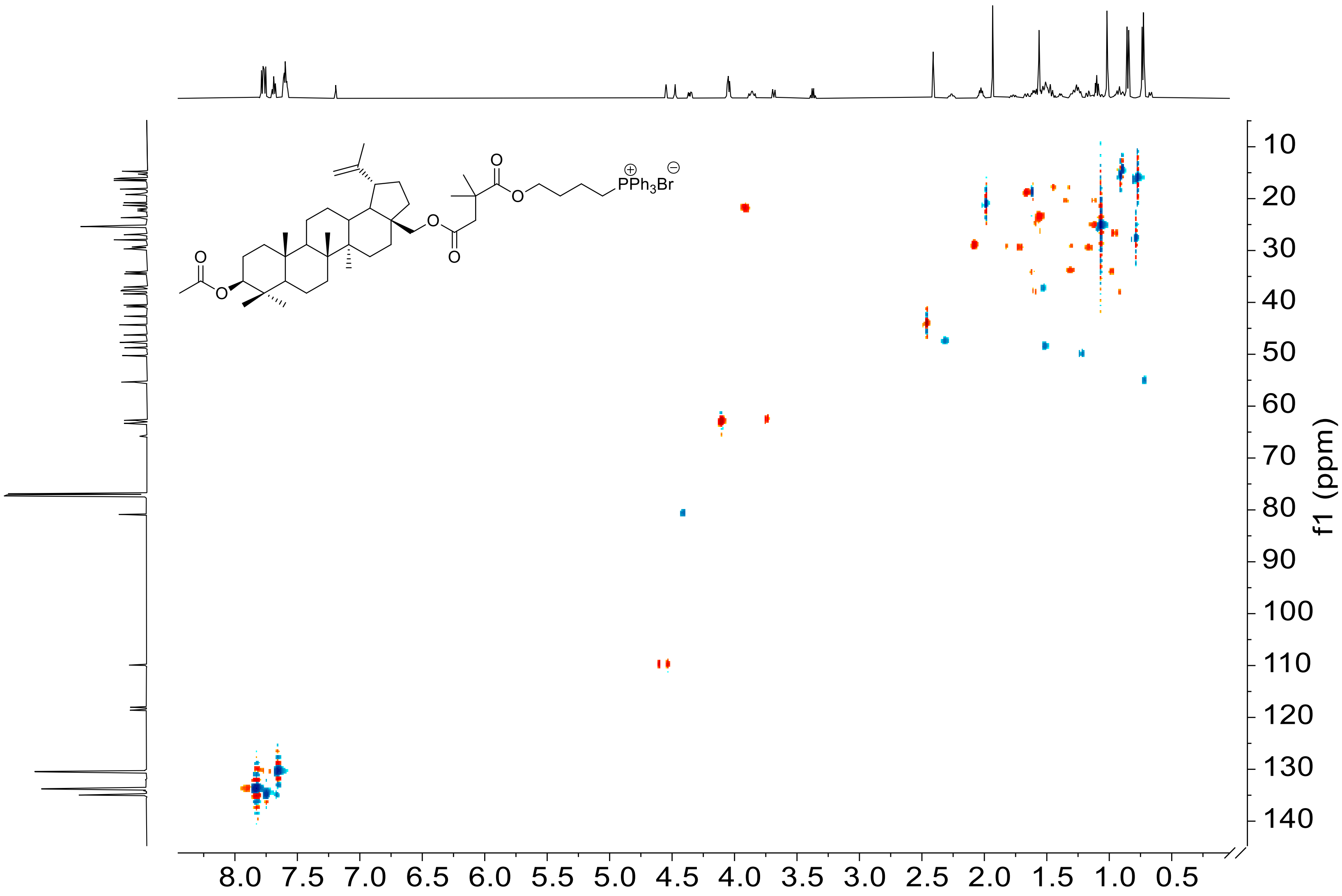

gHSQC spectrum of analogue **7e**

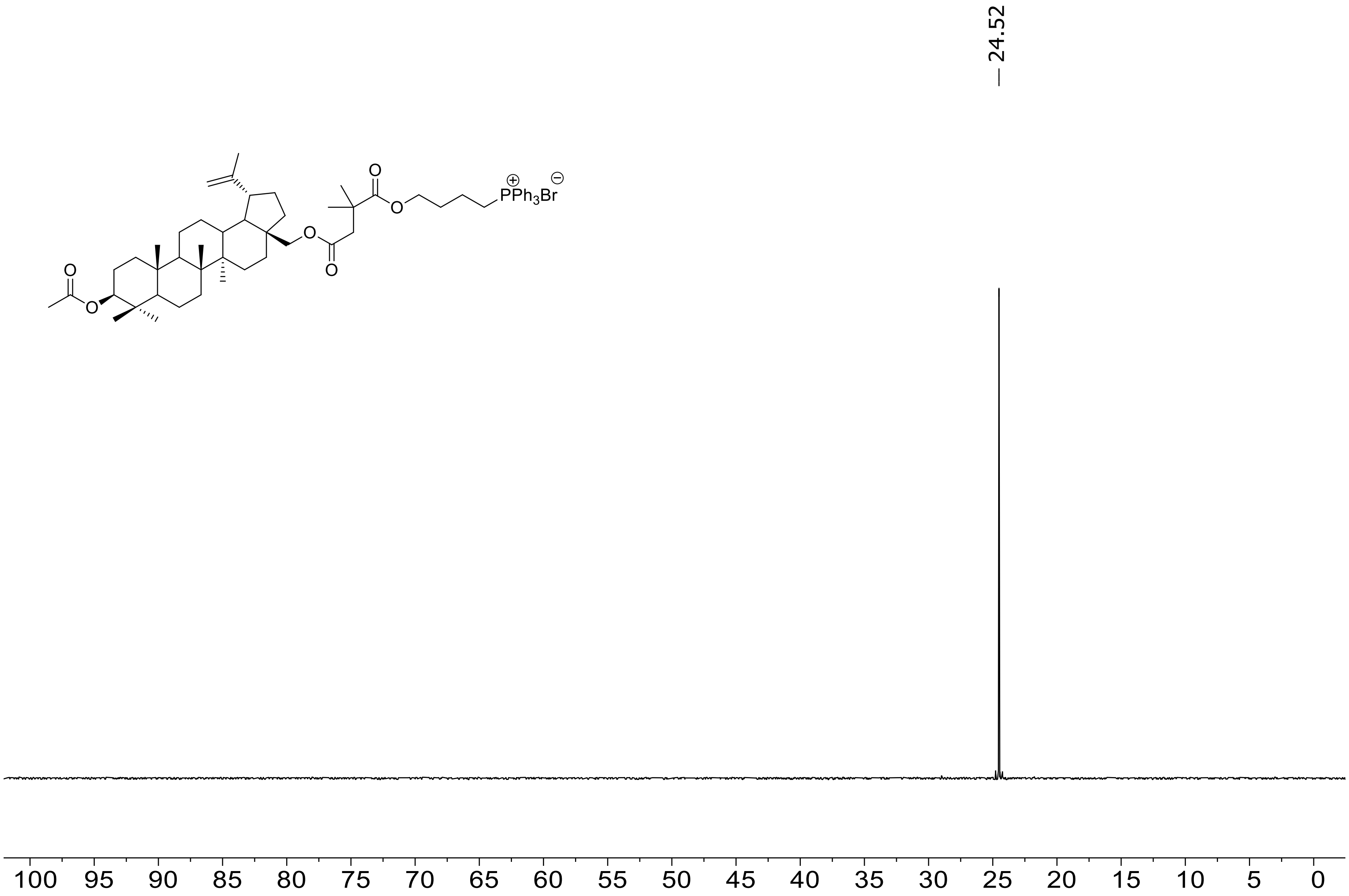

^{31}P NMR spectrum of analogue **7e**; 161.9 MHz/$CDCl_3$/TMS; δ (ppm).

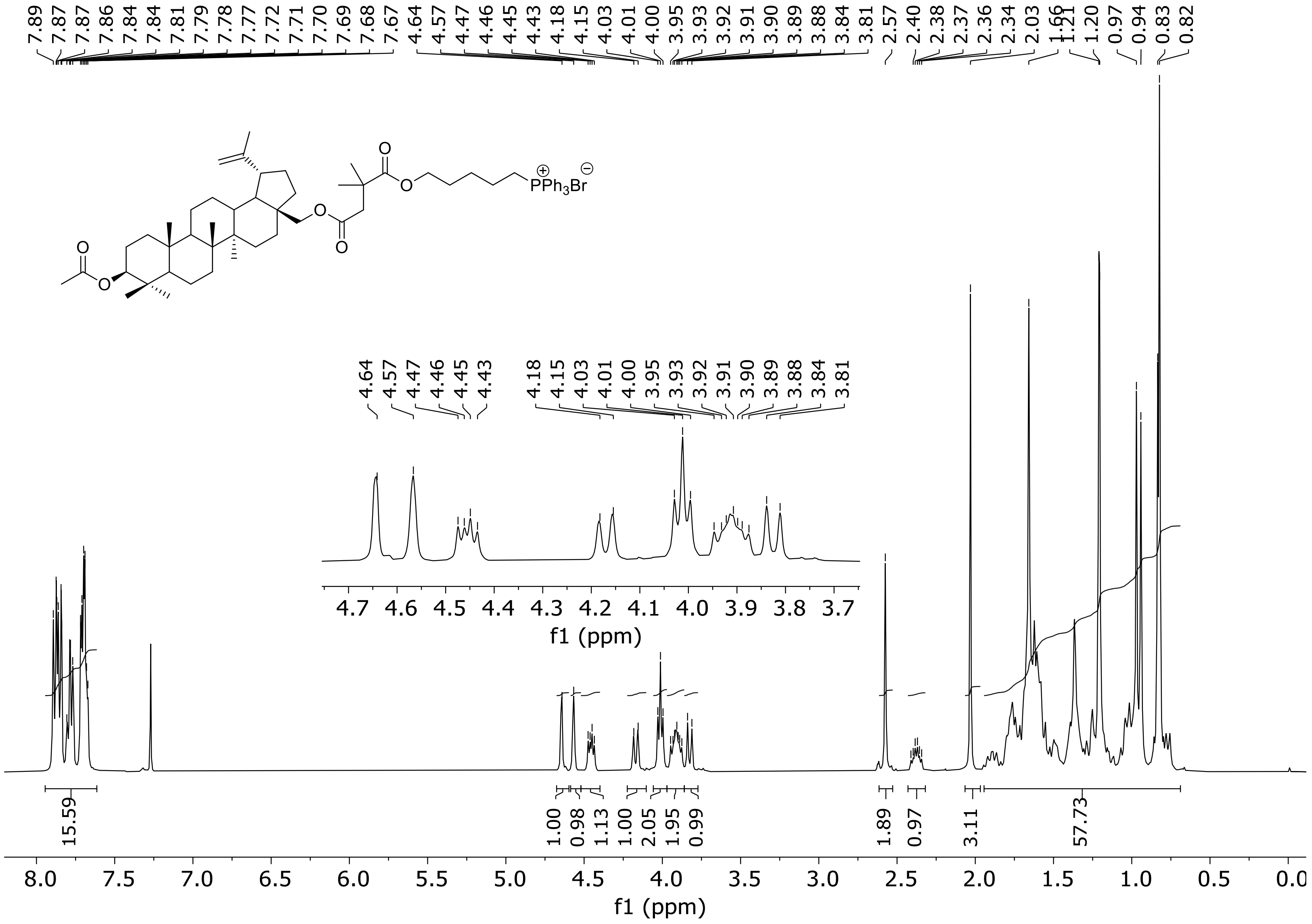

^{1}H NMR spectrum of analogue **7f**; 400 MHz/$CDCl_3$/TMS; *δ* (ppm).

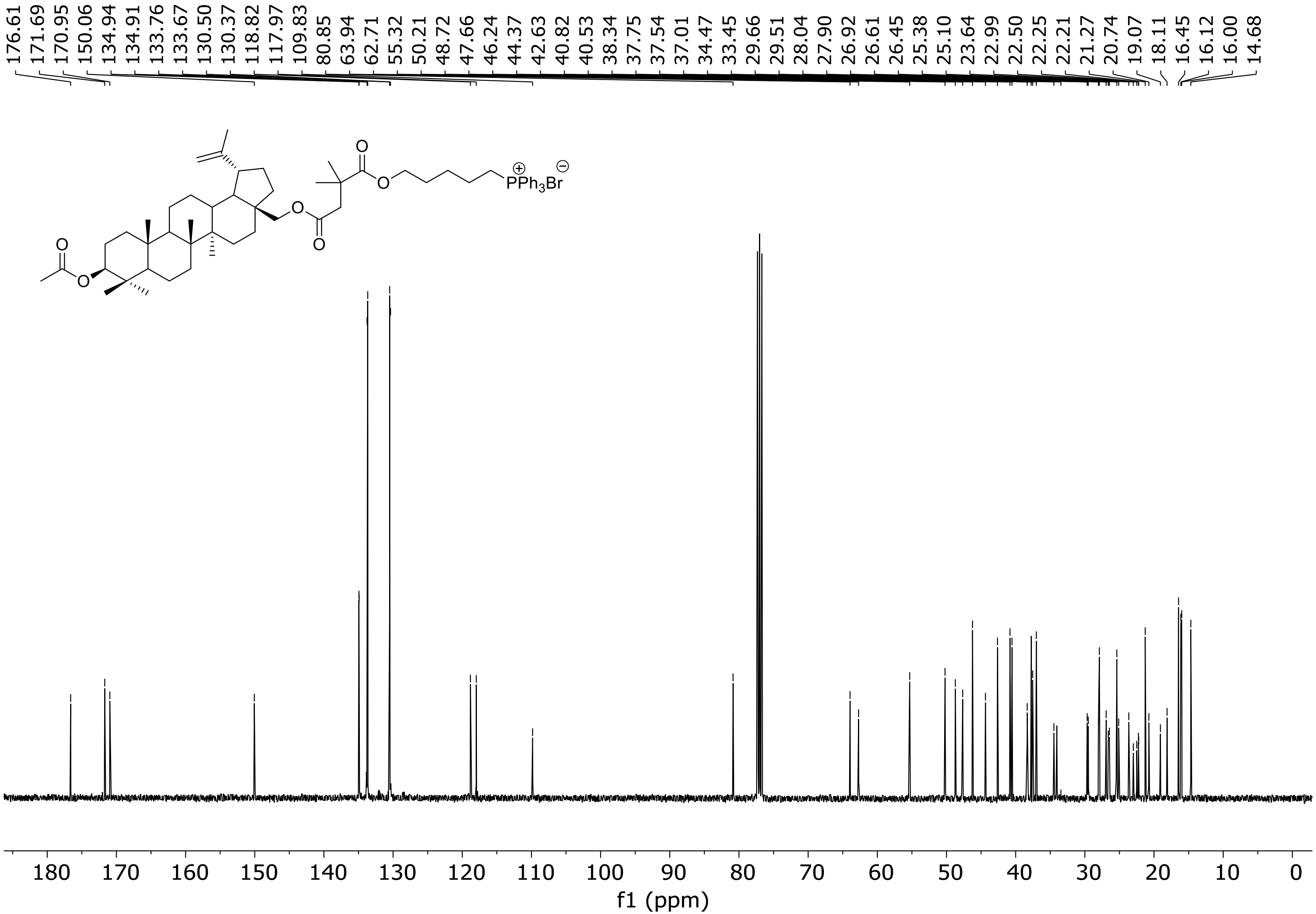

^{13}C NMR spectrum of analogue **7f**; 100 MHz/$CDCl_3$/TMS; δ (ppm).

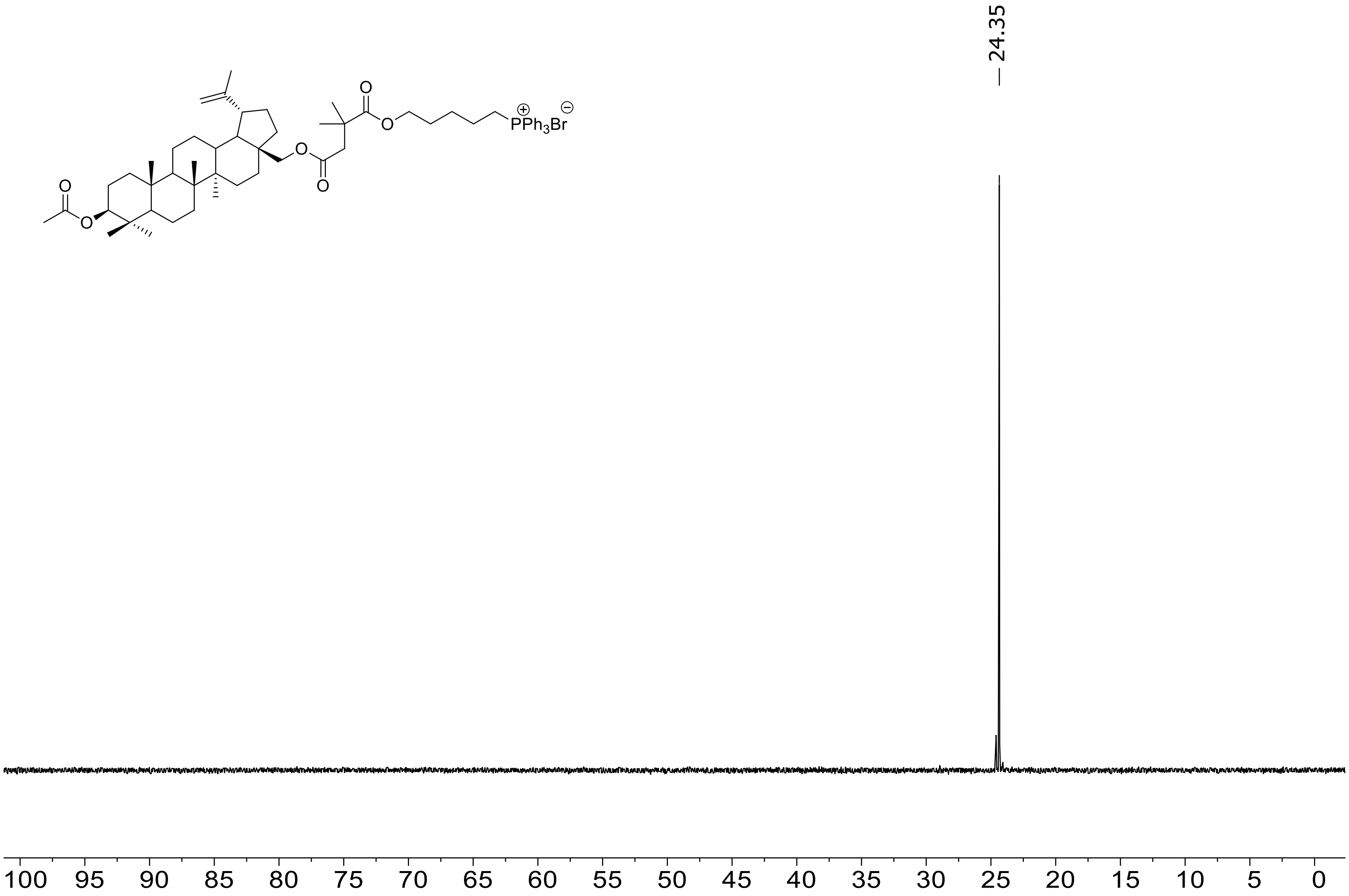

^{31}P NMR spectrum of analogue **7f**; 161.9 MHz/$CDCl_3$/TMS; δ (ppm).

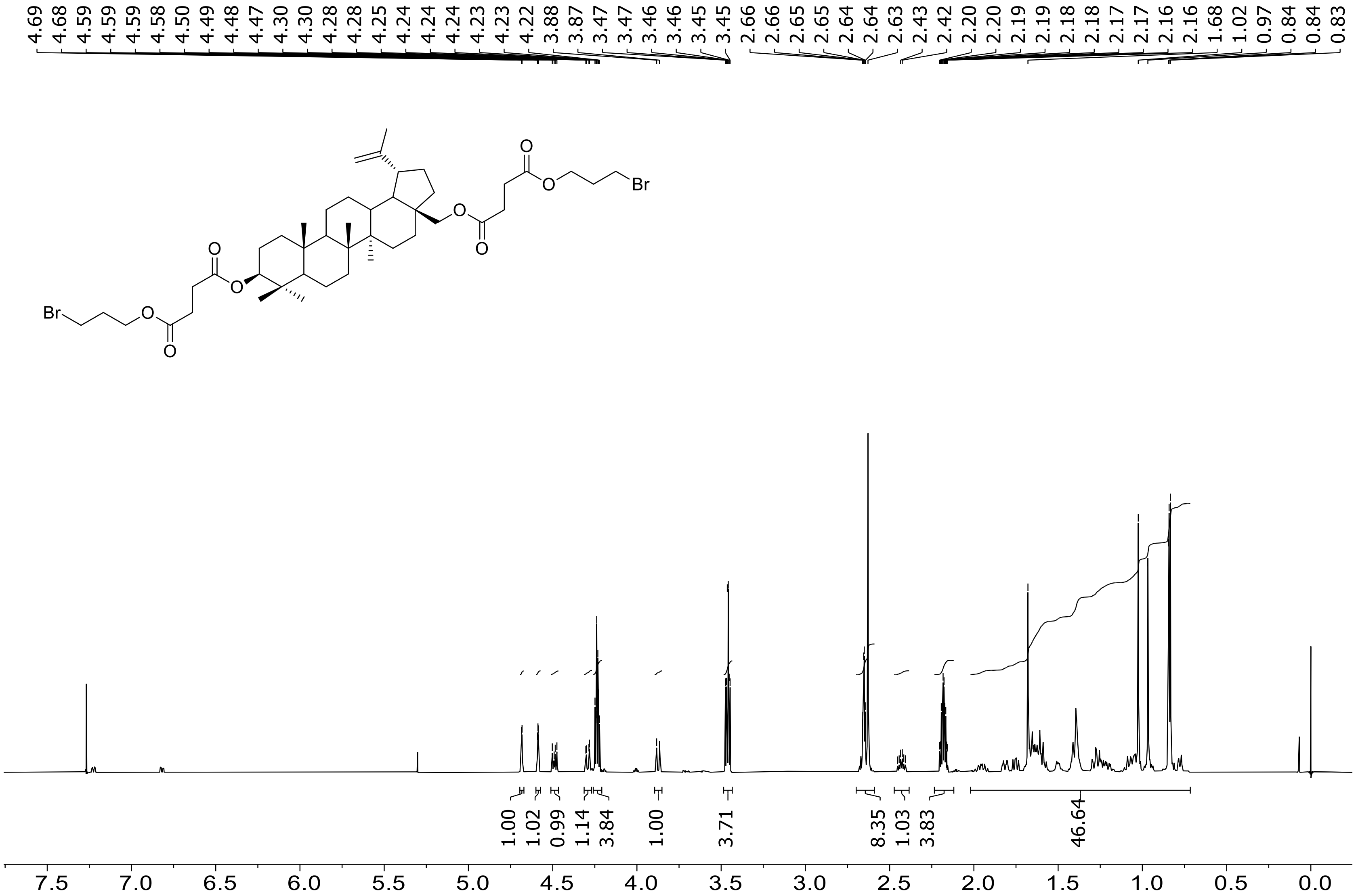

^{1}H NMR spectrum of analogue **8a**; 600 MHz/$CDCl_3$/TMS; δ (ppm).

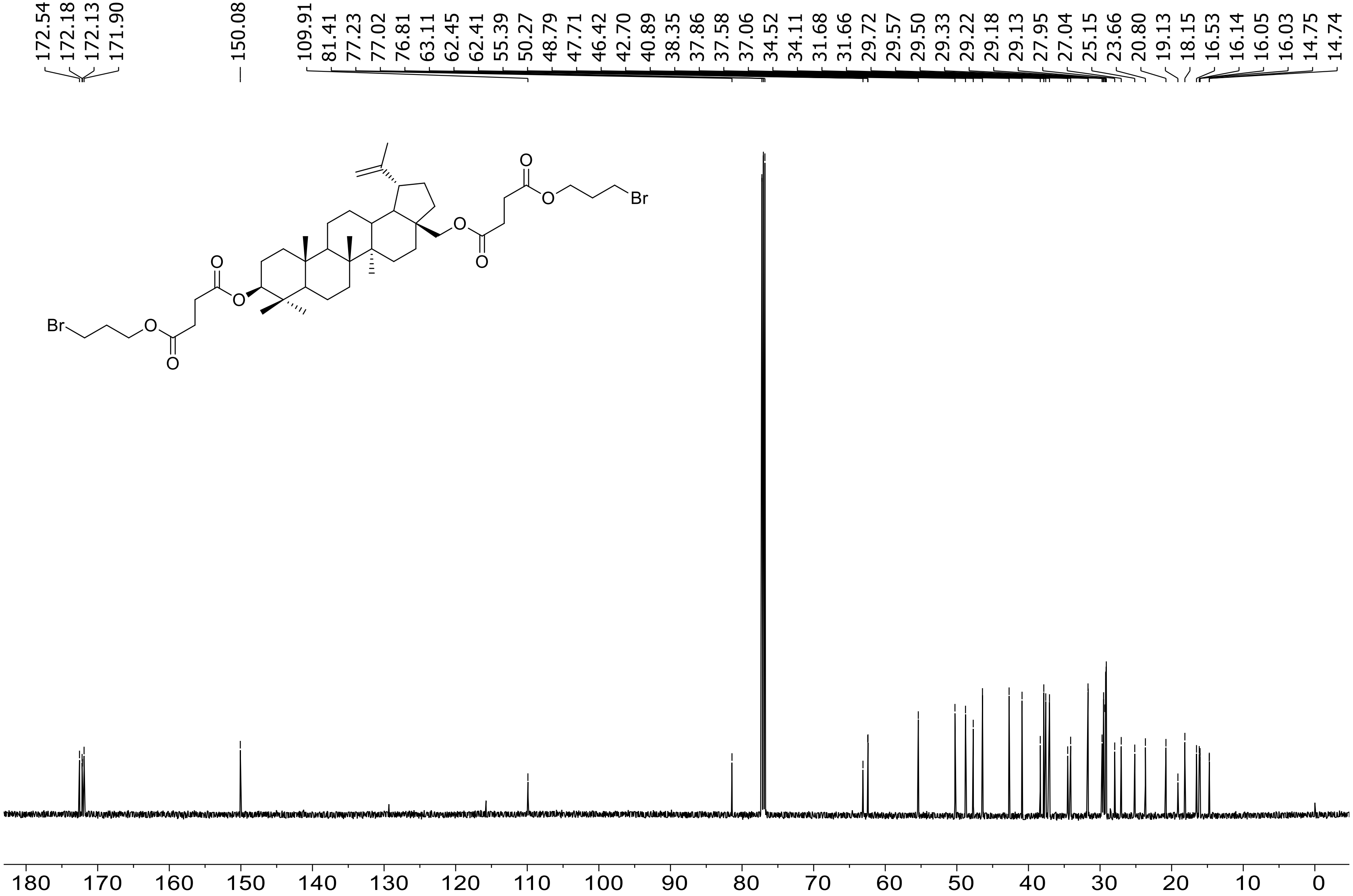

^{13}C NMR spectrum of analogue **8a**; 150 MHz/CDCl$_3$/TMS; δ (ppm).

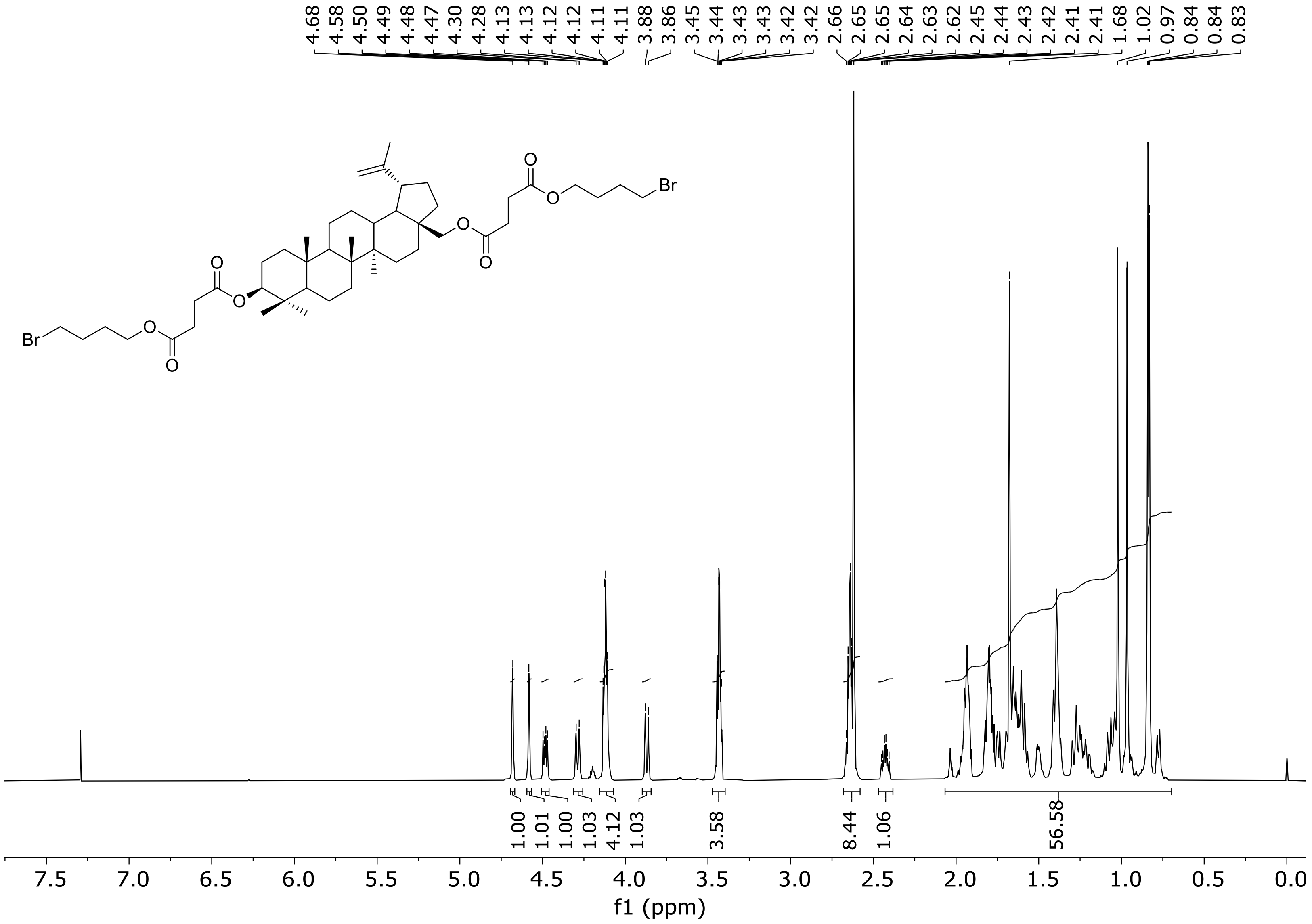

^{1}H NMR spectrum of analogue **8b**; 600 MHz/$CDCl_3$/TMS; δ (ppm).

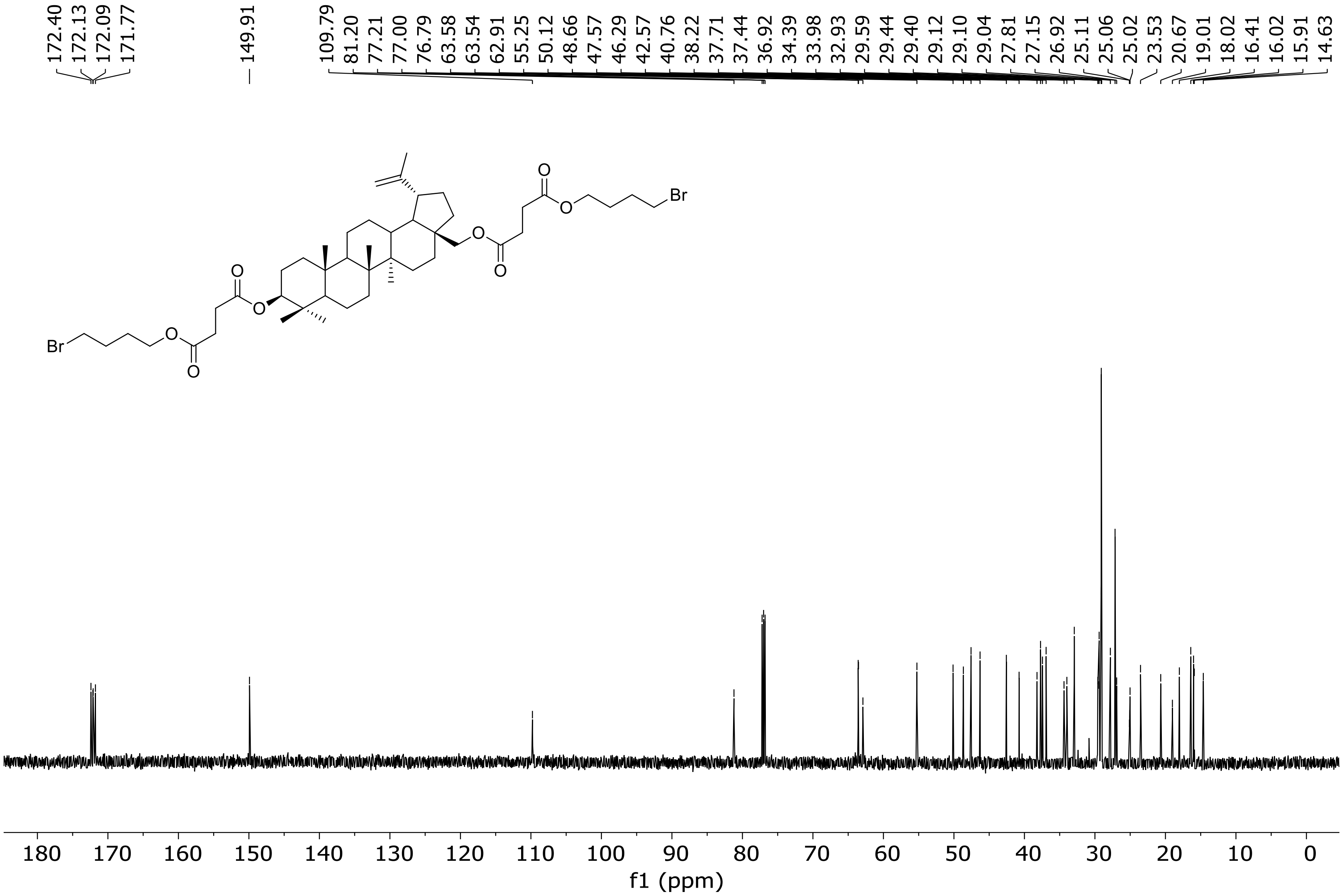

^{13}C NMR spectrum of analogue **8b**; 150 MHz/$CDCl_3$/TMS; δ (ppm).

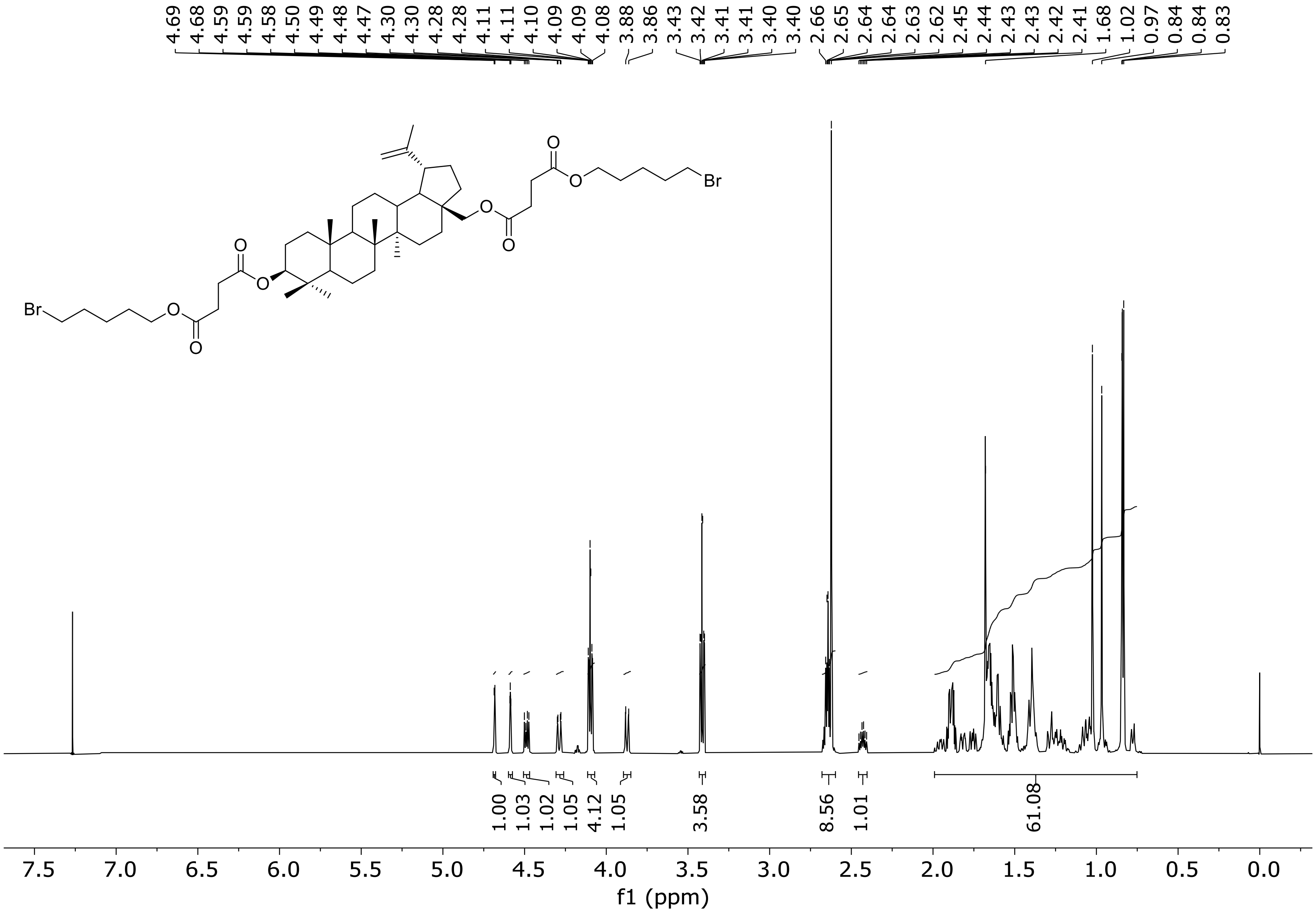

^{1}H NMR spectrum of analogue **8c**; 600 MHz/$CDCl_3$/TMS; δ (ppm).

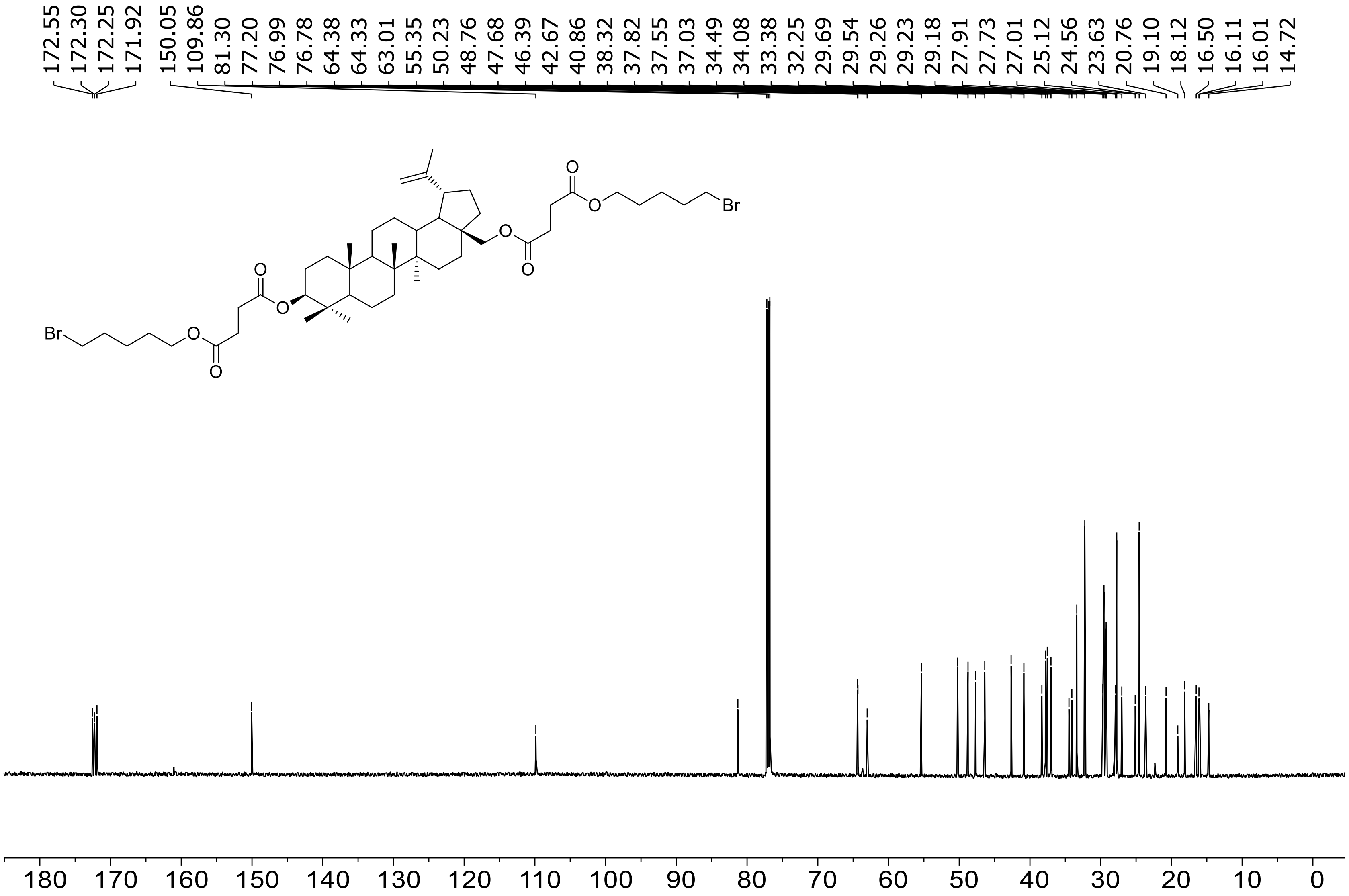

^{13}C NMR spectrum of analogue **8c**; 150 MHz/CDCl$_3$/TMS; δ (ppm).

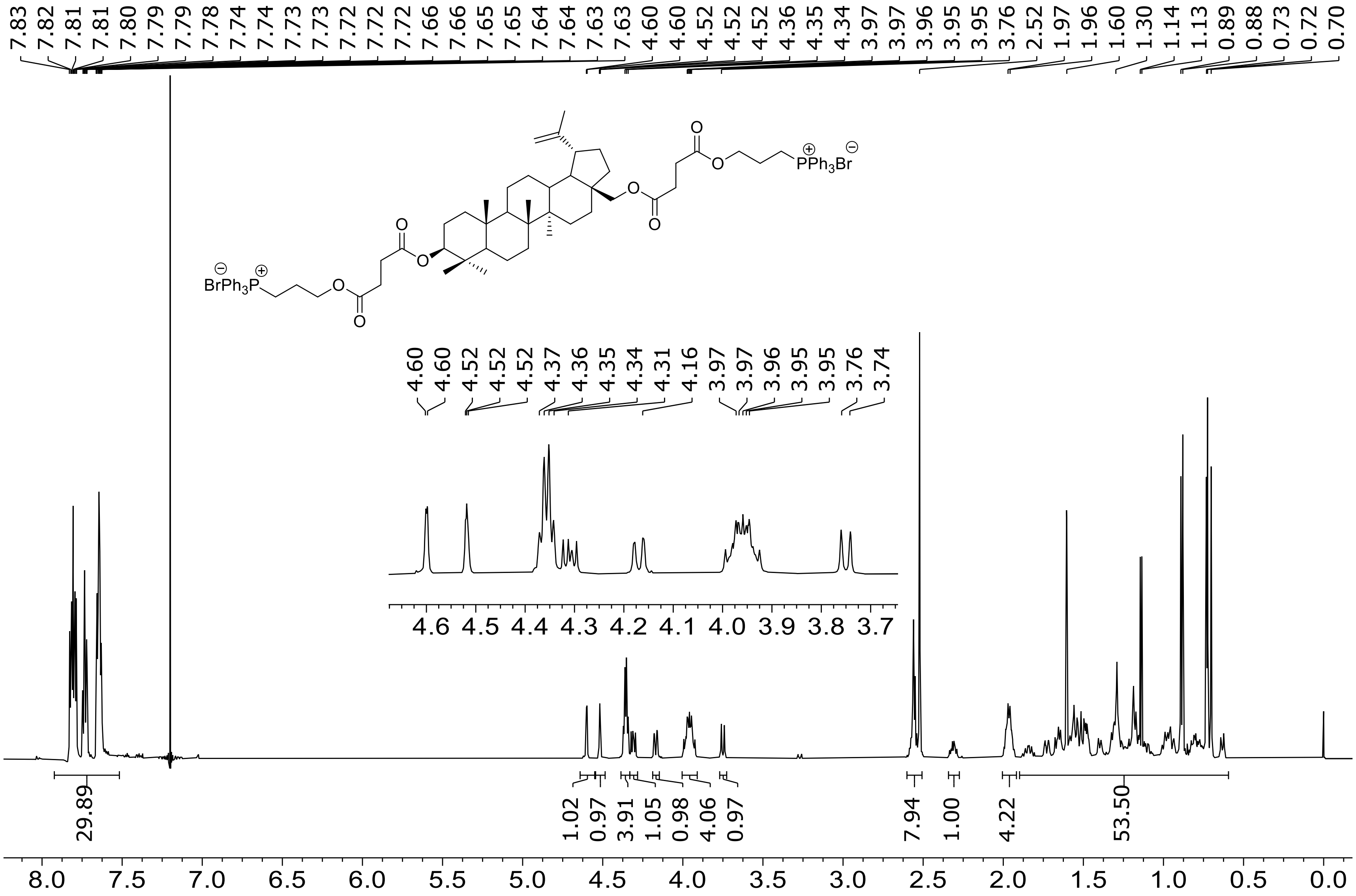

^{1}H NMR spectrum of analogue **9a**; 600 MHz/$CDCl_3$/TMS; δ (ppm).

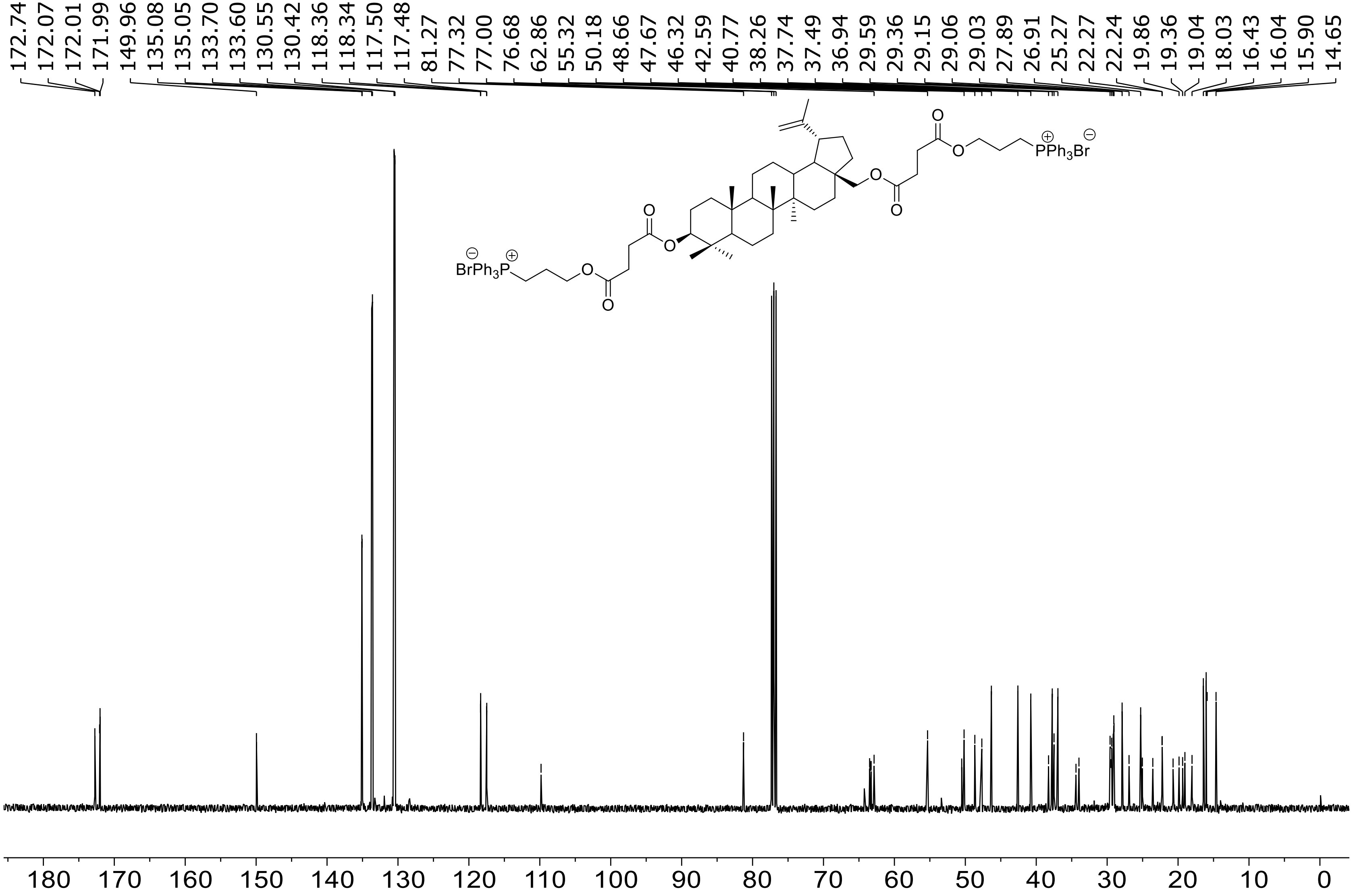

^{13}C NMR spectrum of analogue **9a**; 150 MHz/$CDCl_3$/TMS; δ (ppm).

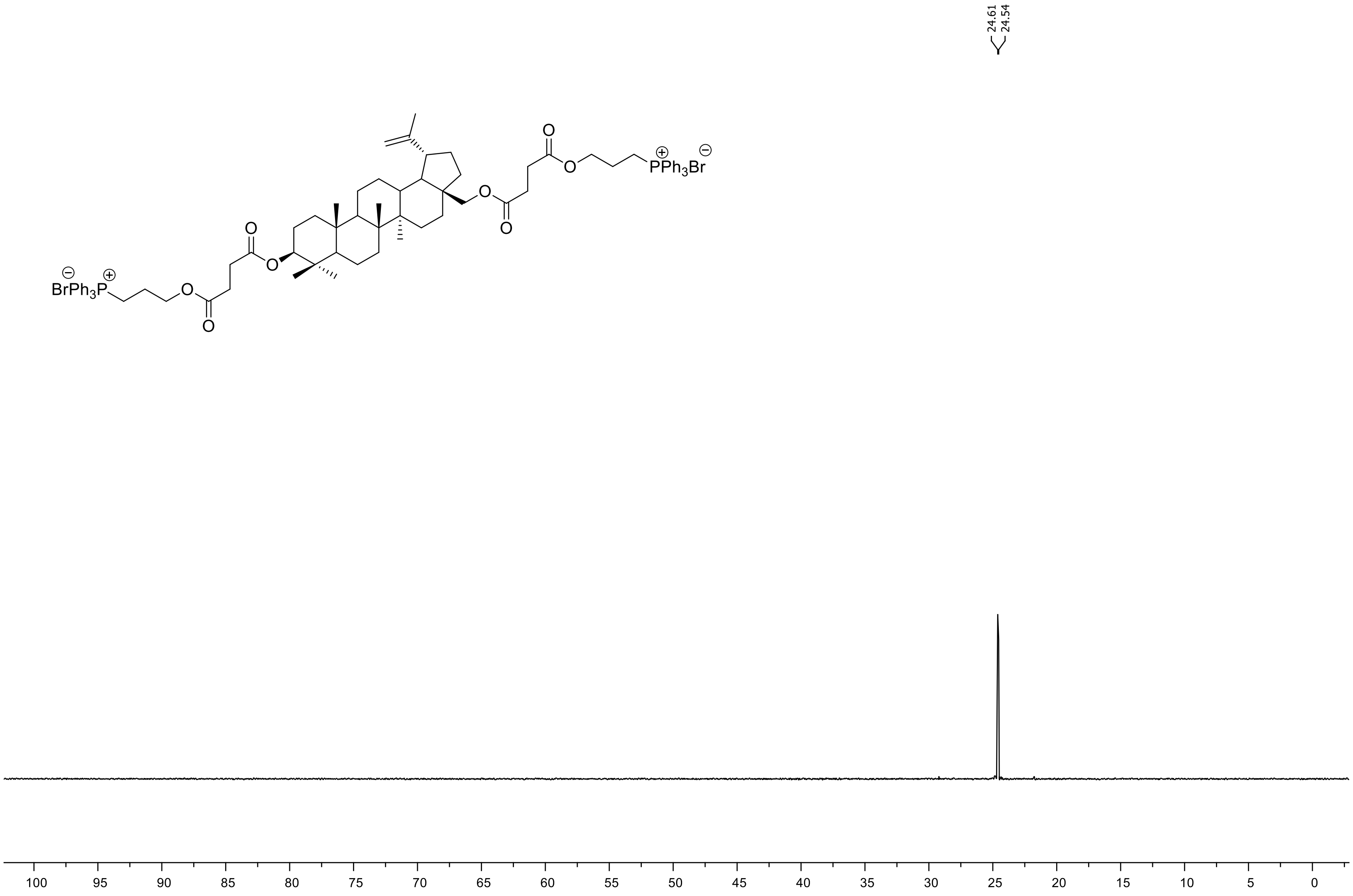

^{31}P NMR spectrum of analogue **9a**; 161.9 MHz/$CDCl_3$/TMS; δ (ppm).

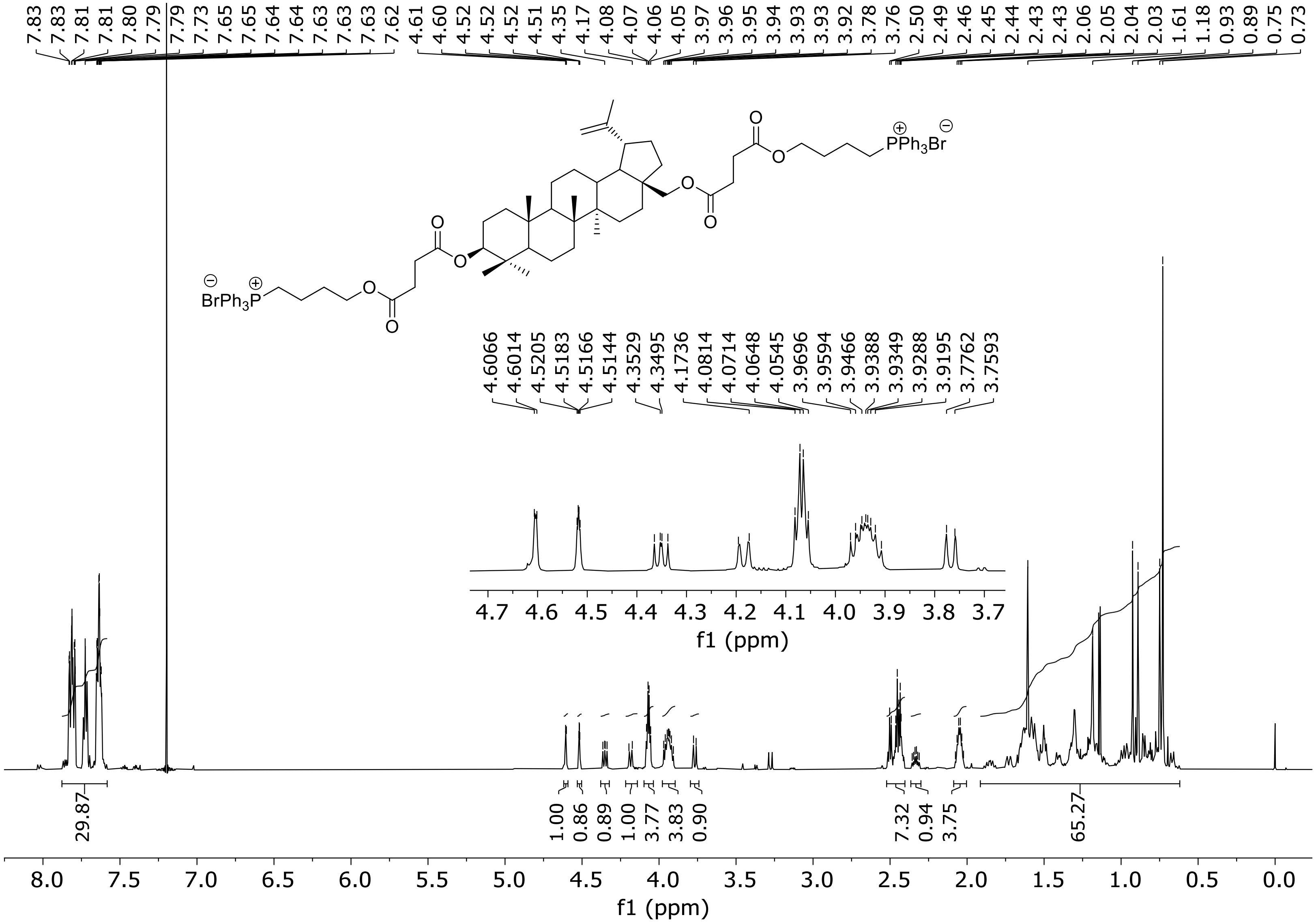

^{1}H NMR spectrum of analogue **9b**; 600 MHz/$CDCl_3$/TMS; δ (ppm).

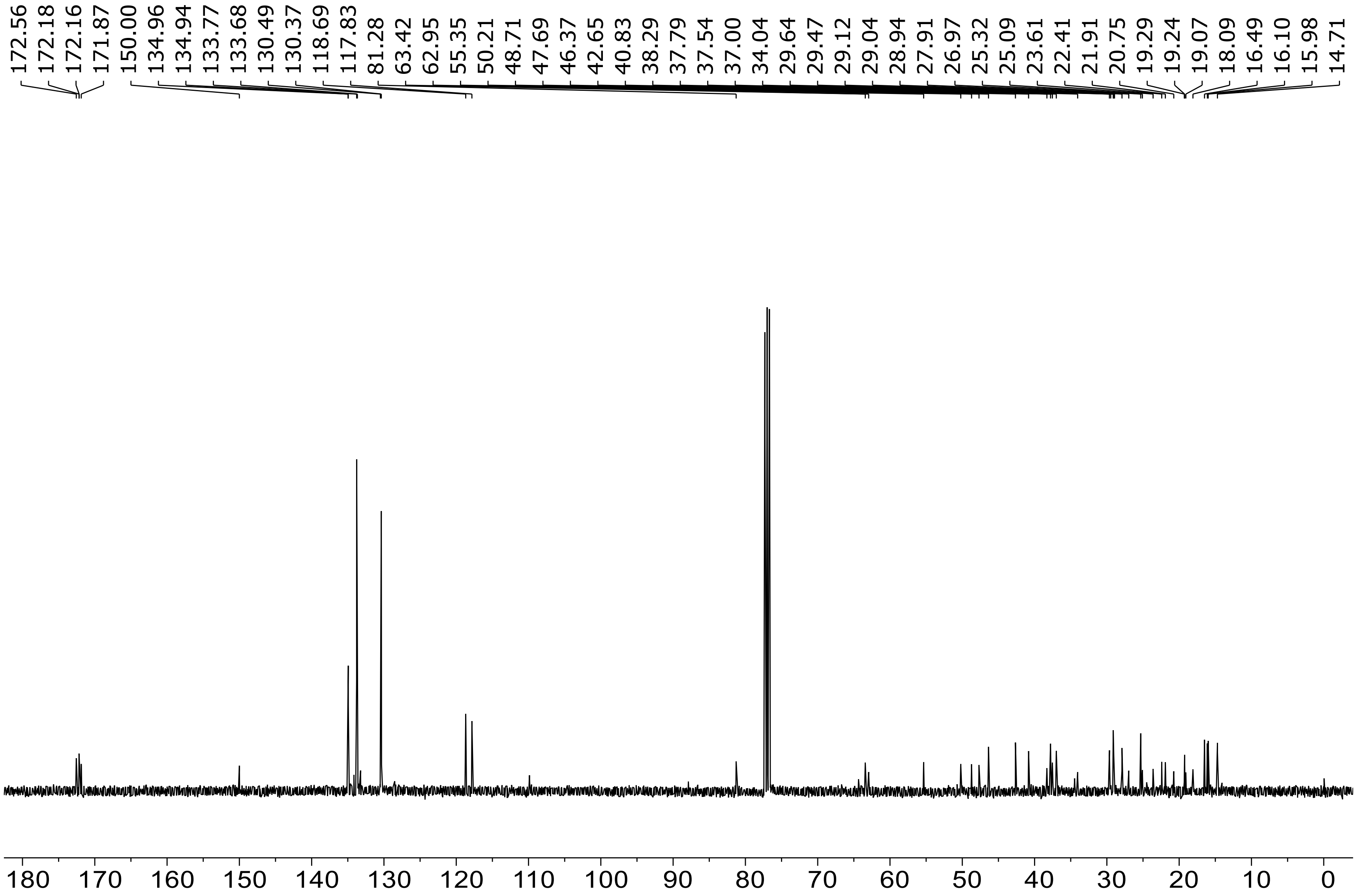

^{13}C NMR spectrum of analogue **9b**; 100 MHz/$CDCl_3$/TMS; δ (ppm).

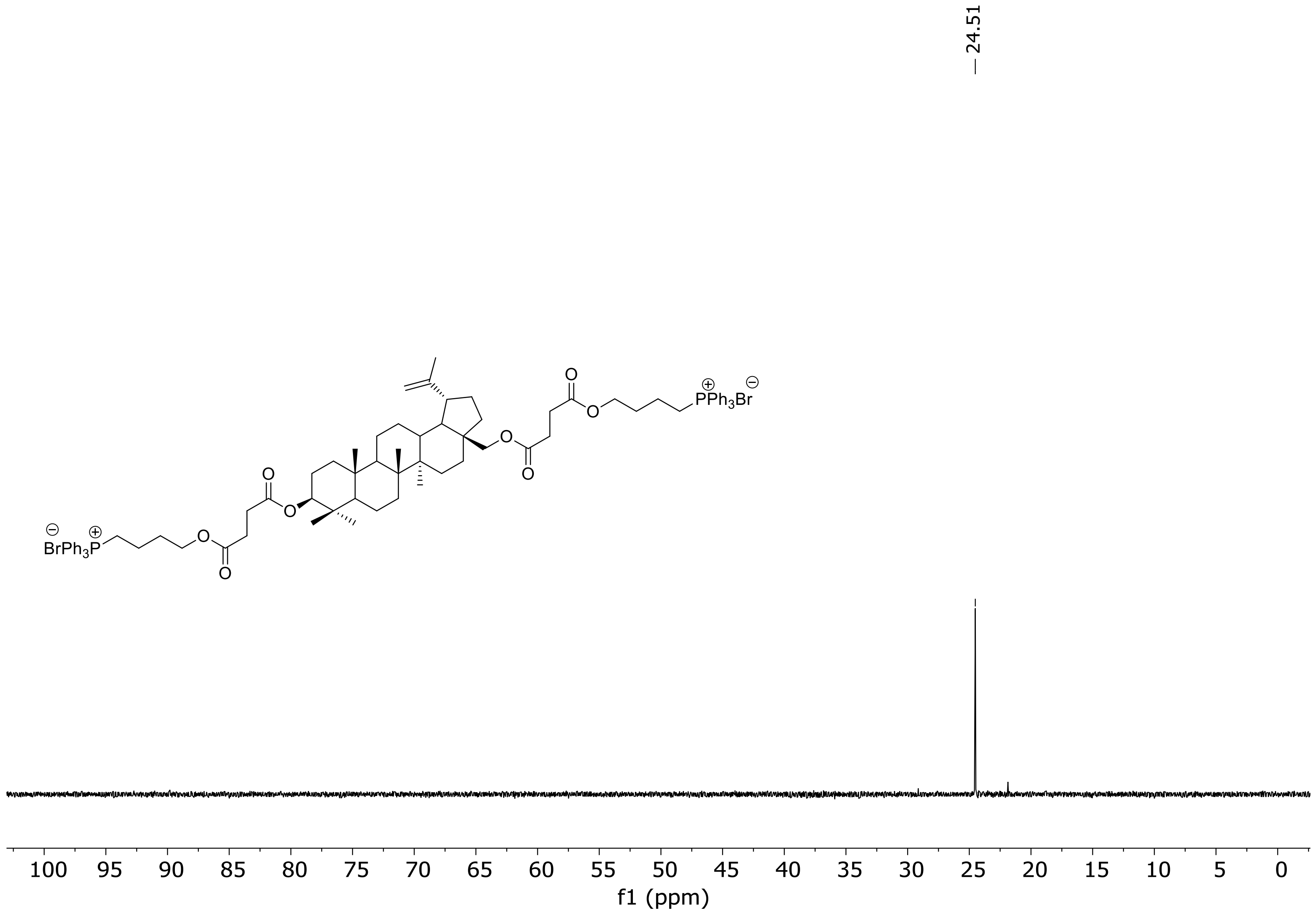

^{31}P NMR spectrum of analogue **9b**; 161.9 MHz/$CDCl_3$/TMS; *δ* (ppm).

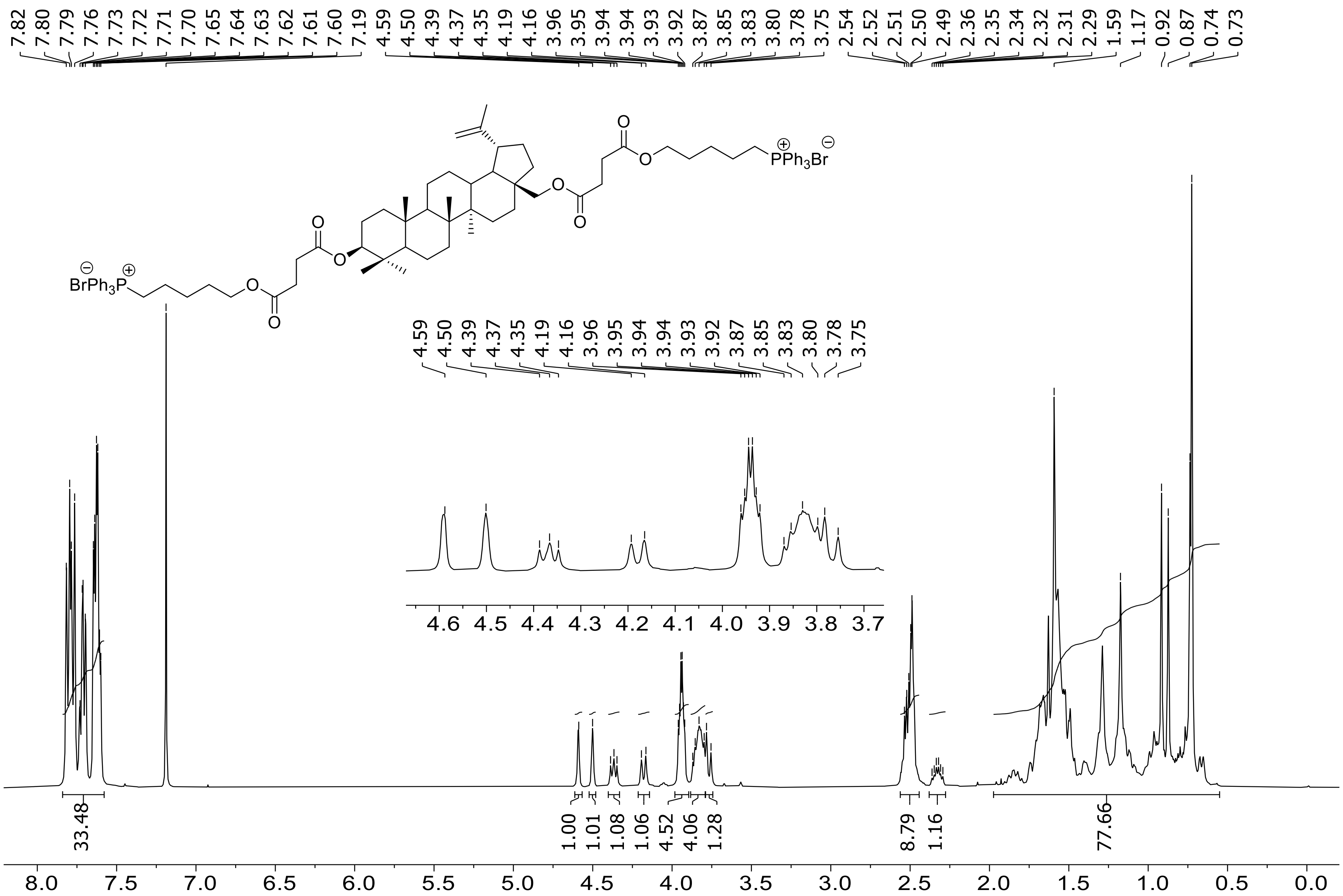

^{1}H NMR spectrum of analogue **9c**; 400 MHz/$CDCl_3$/TMS; δ (ppm).

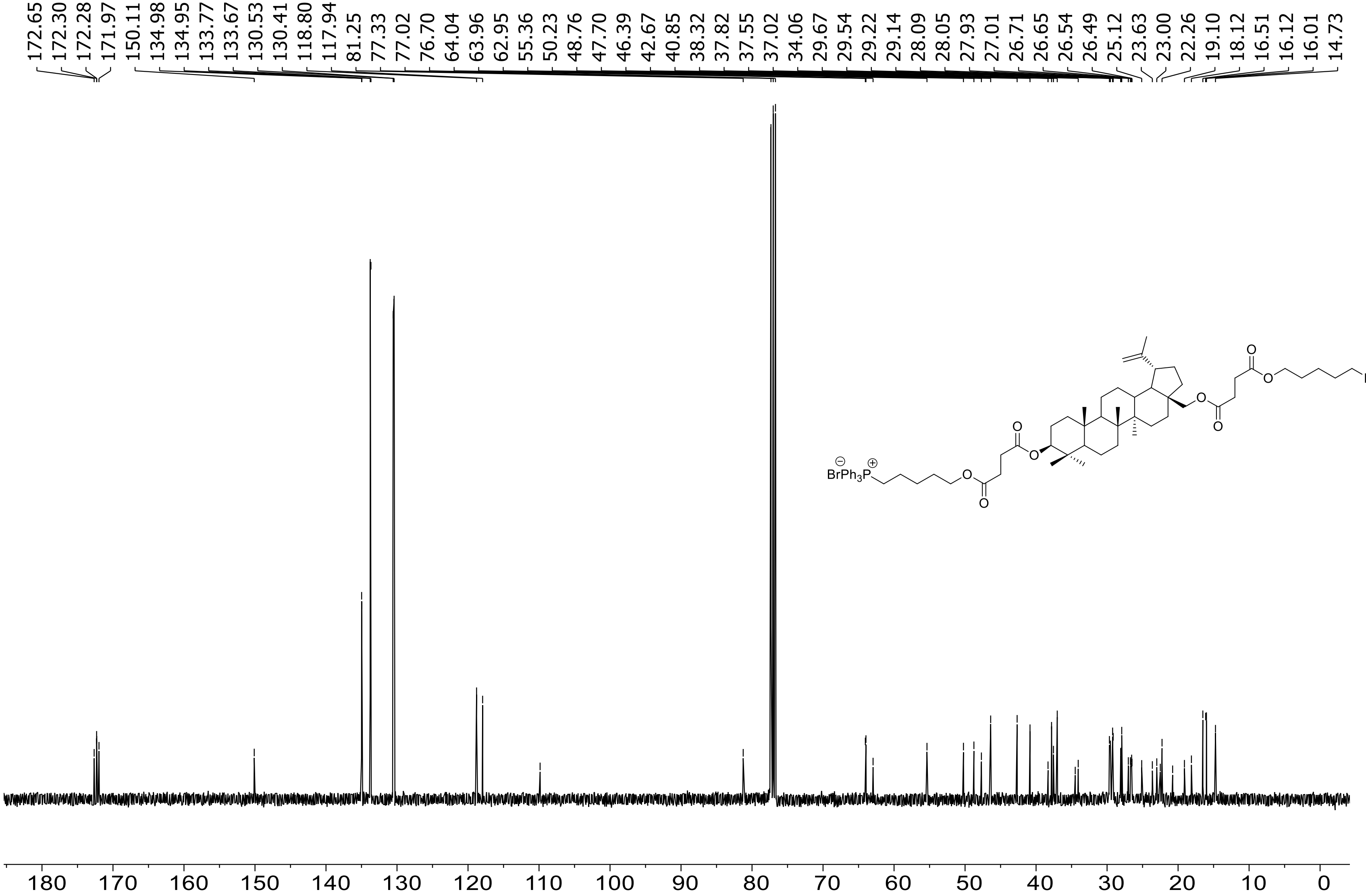

^{13}C NMR spectrum of analogue **9c**; 100 MHz/CDCl$_3$/TMS; δ (ppm).

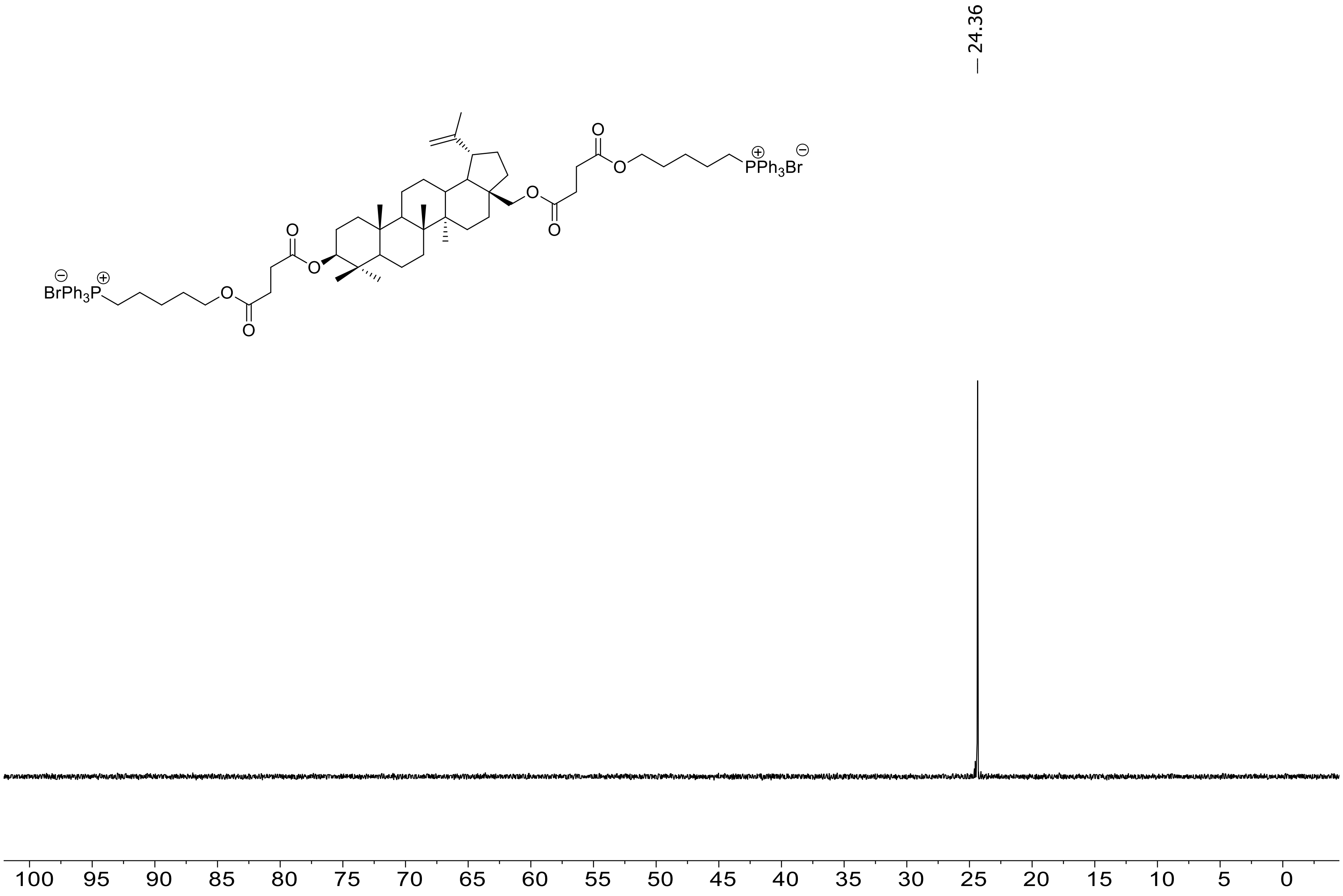

^{31}P NMR spectrum of analogue **9c**; 161.9 MHz/$CDCl_3$/TMS; δ (ppm).

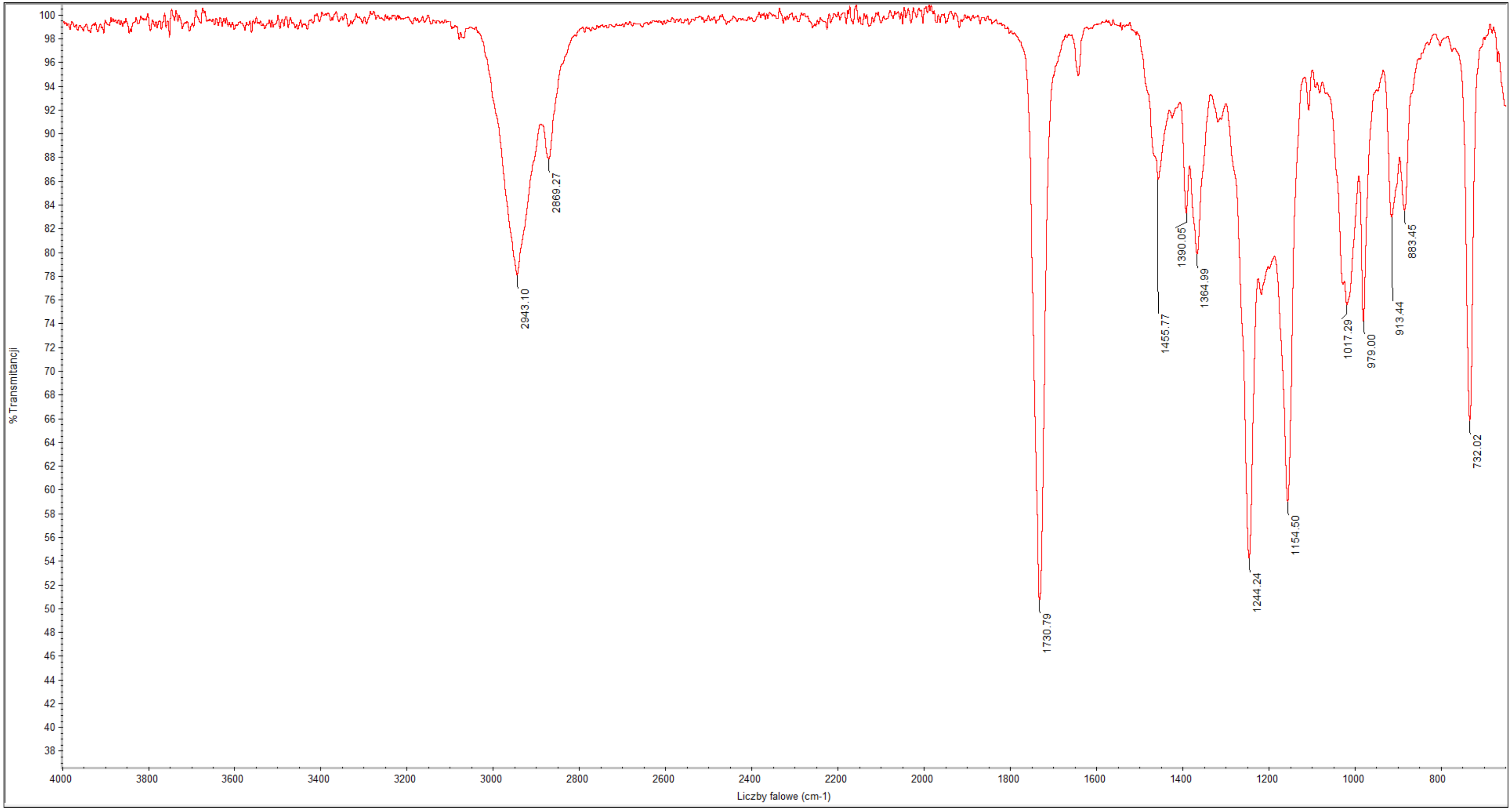

IR spectrum of product **6a**; ATR (cm^{-1}).

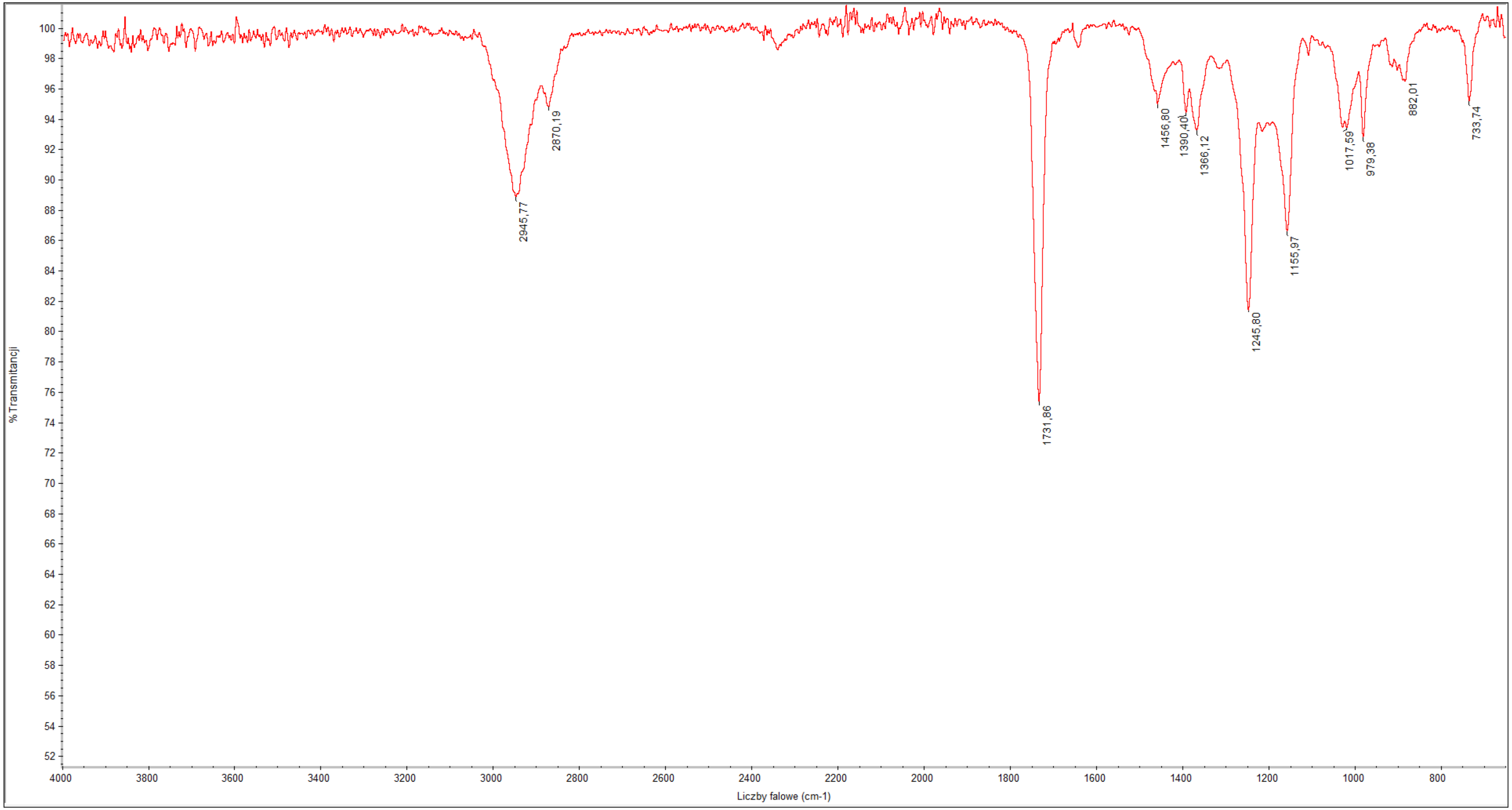

IR spectrum of product **6b**; ATR (cm^{-1}).

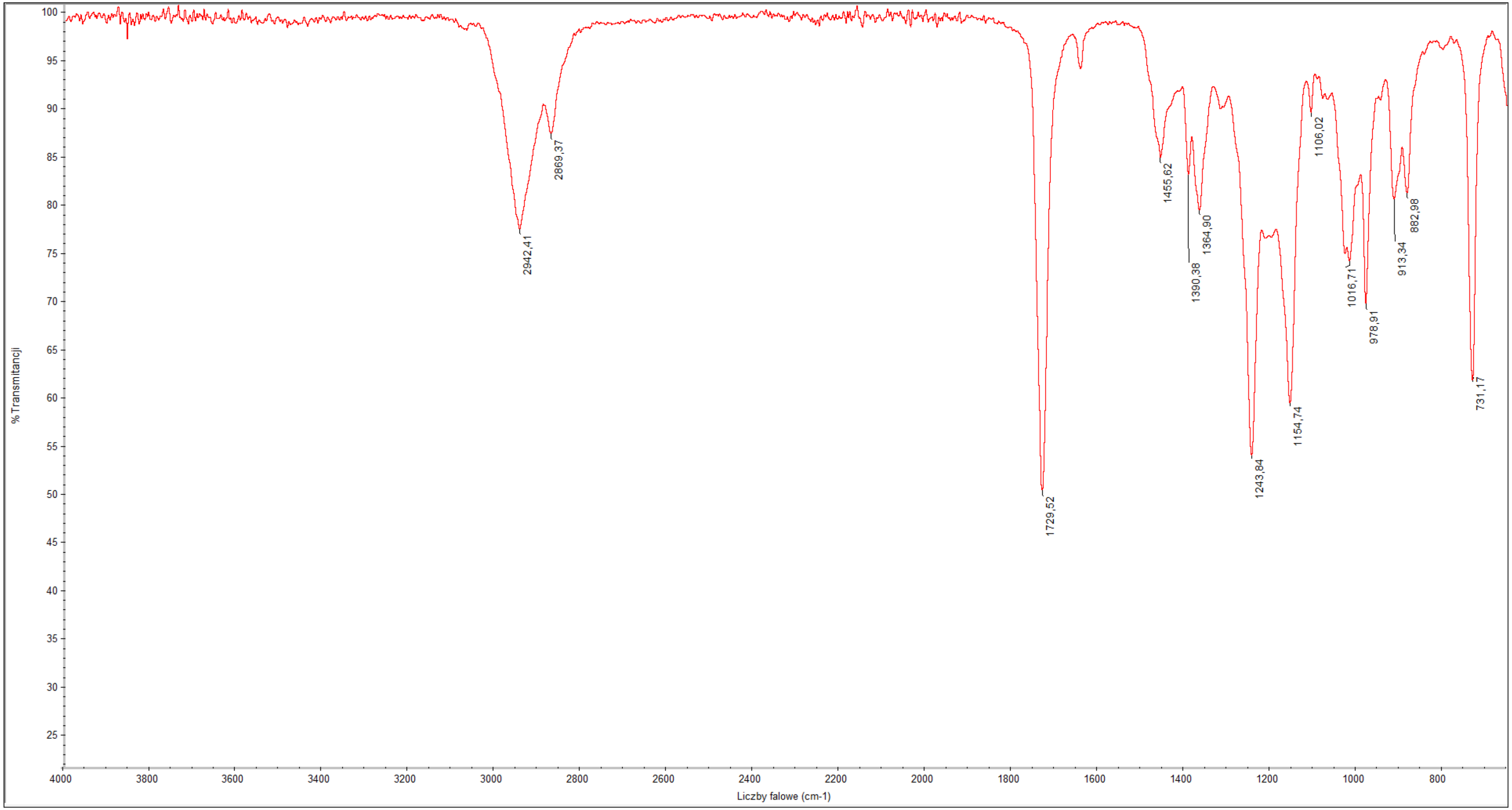

IR spectrum of product **6c**; ATR (cm^{-1}).

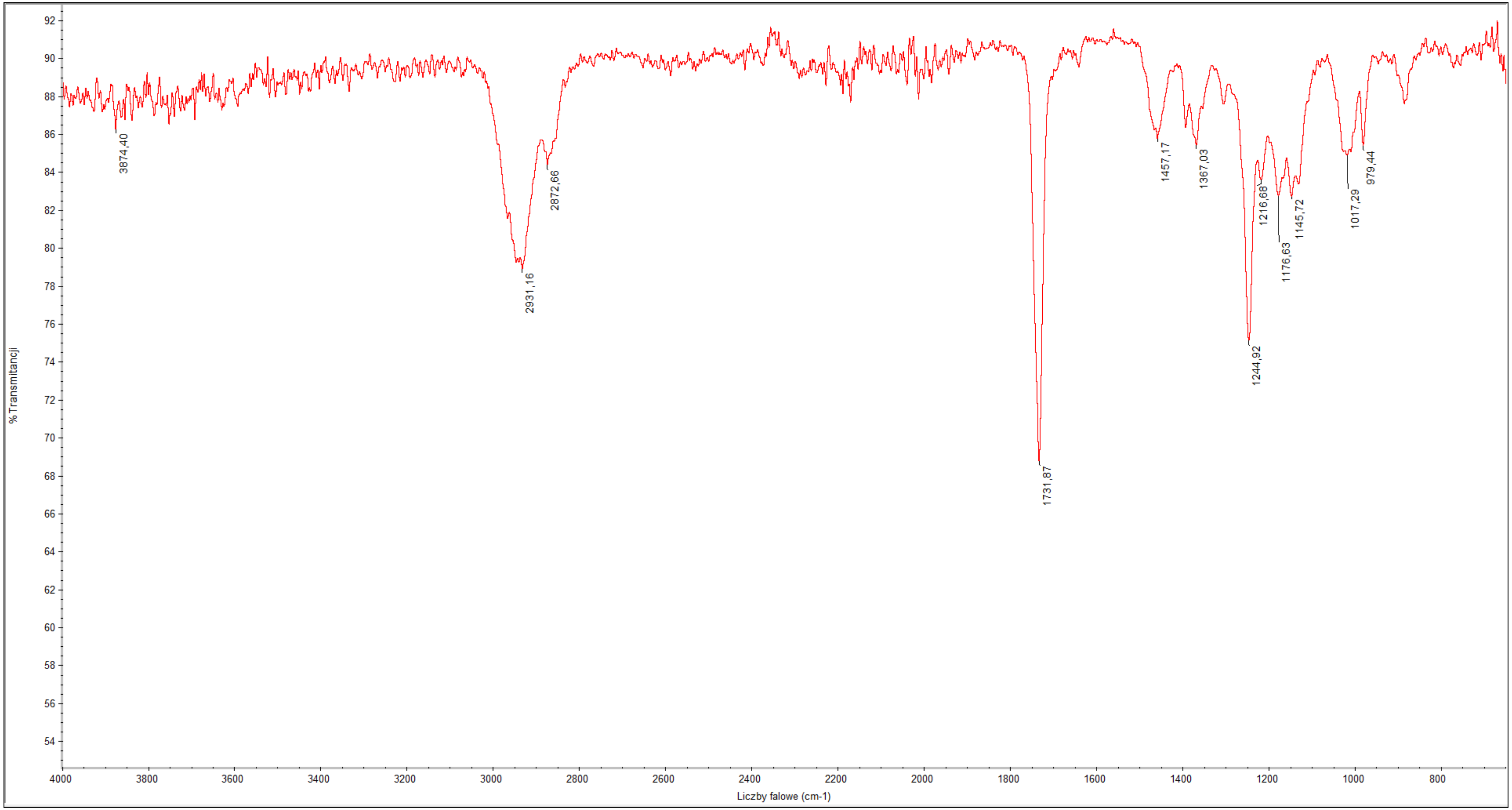

IR spectrum of product **6d**; ATR (cm^{-1}).

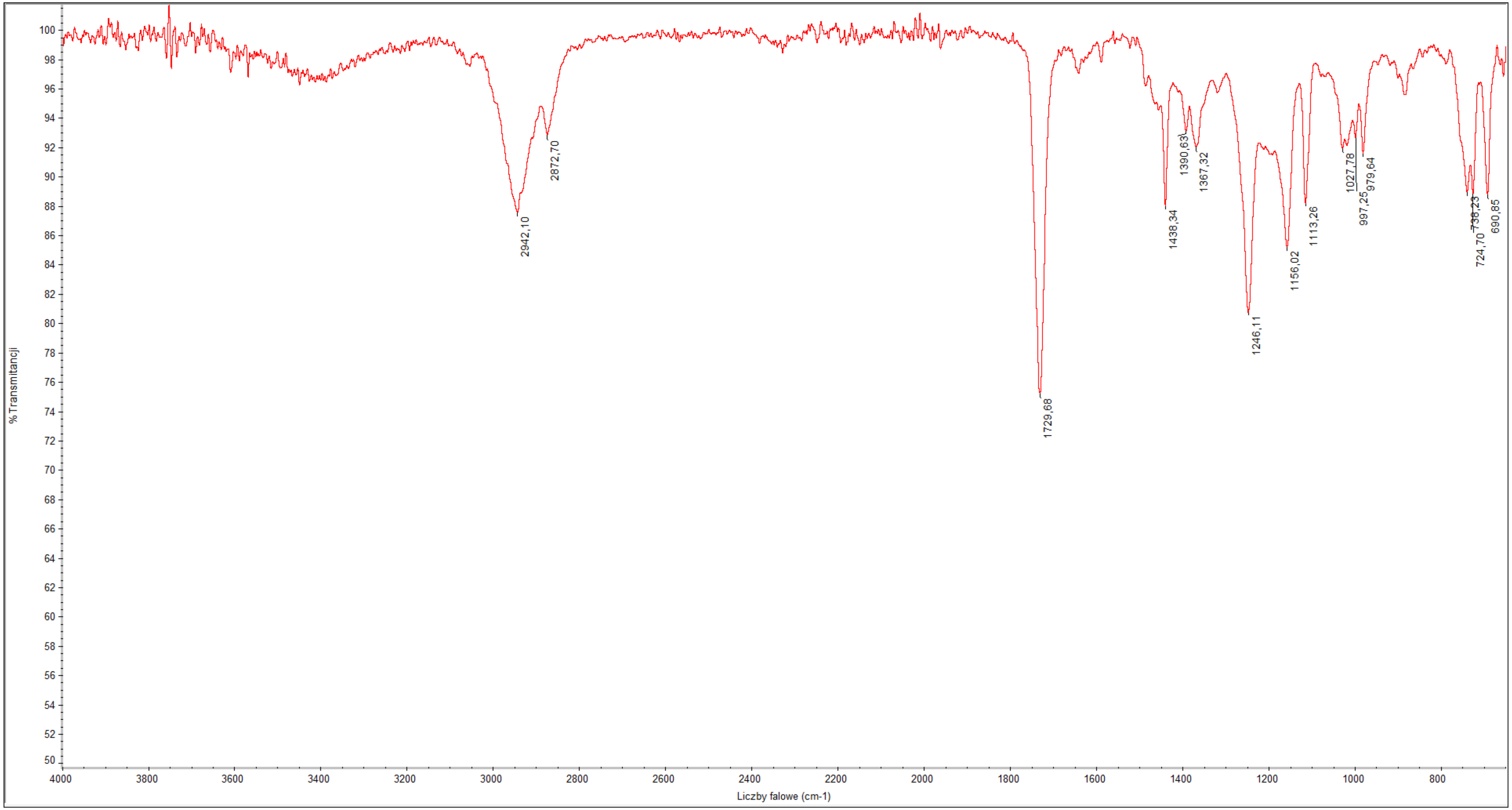

IR spectrum of product **7a**; ATR (cm-1).

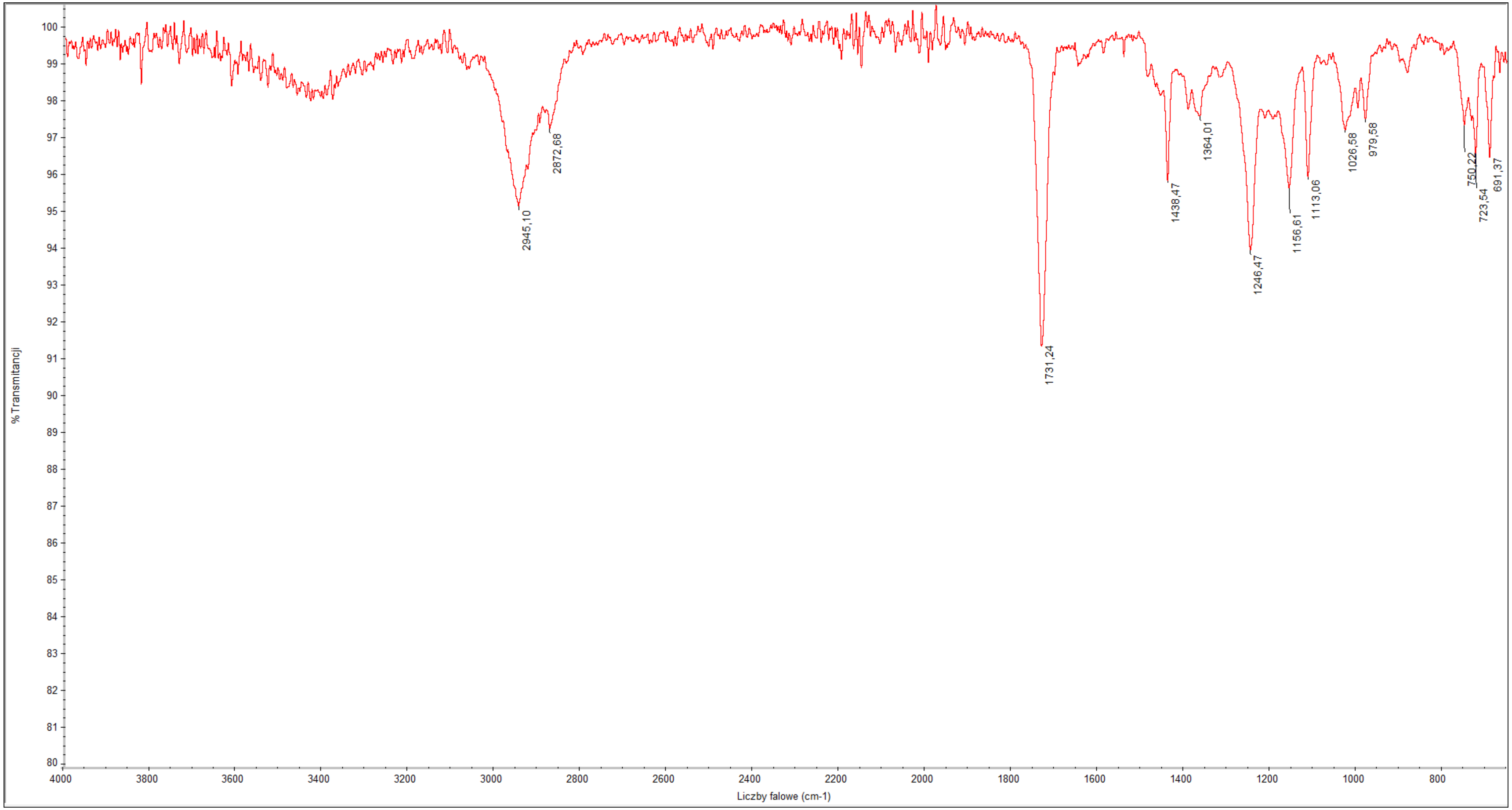

IR spectrum of product **7b**; ATR (cm^{-1}).

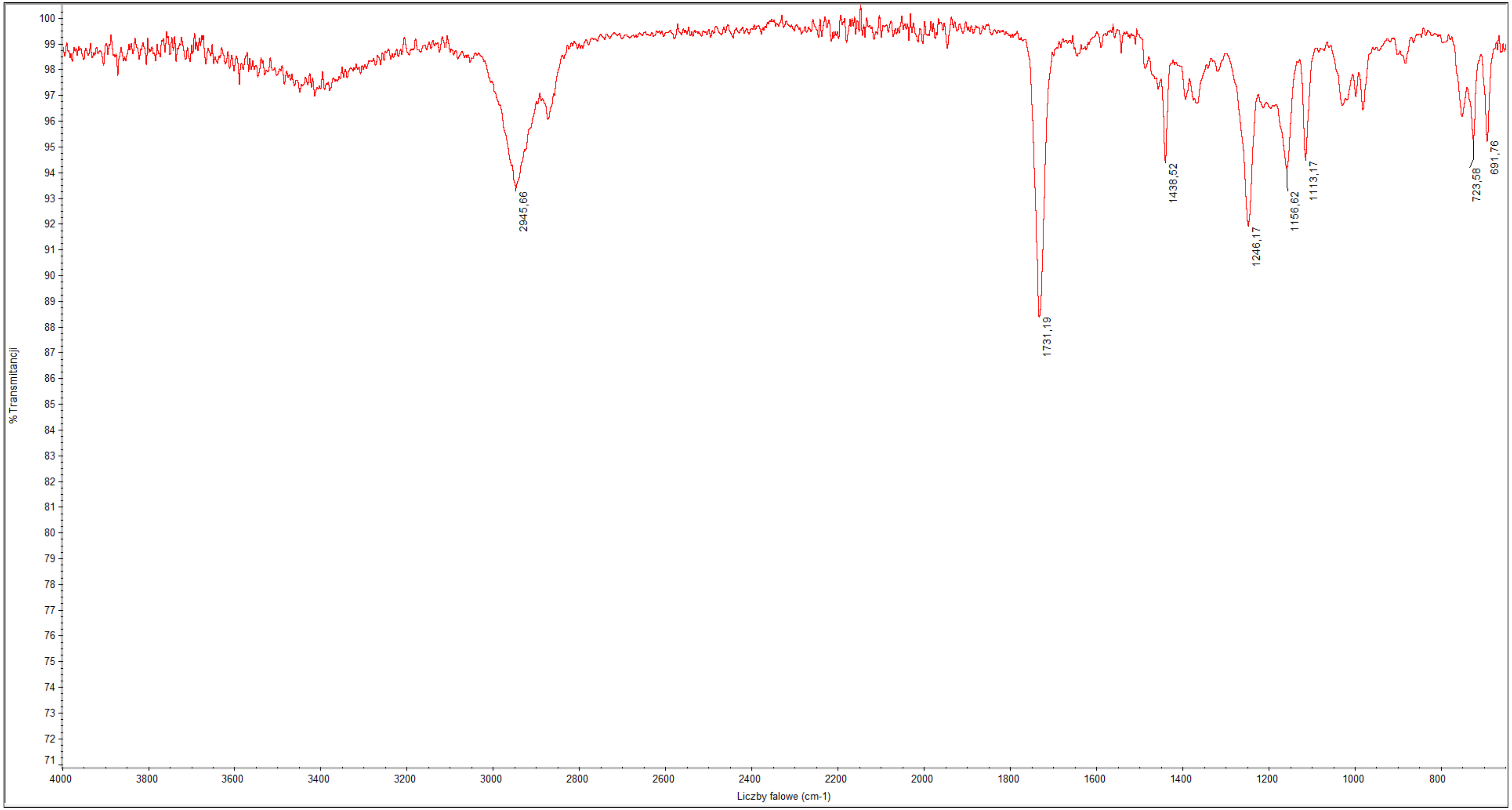

IR spectrum of product **7c**; ATR (cm^{-1}).

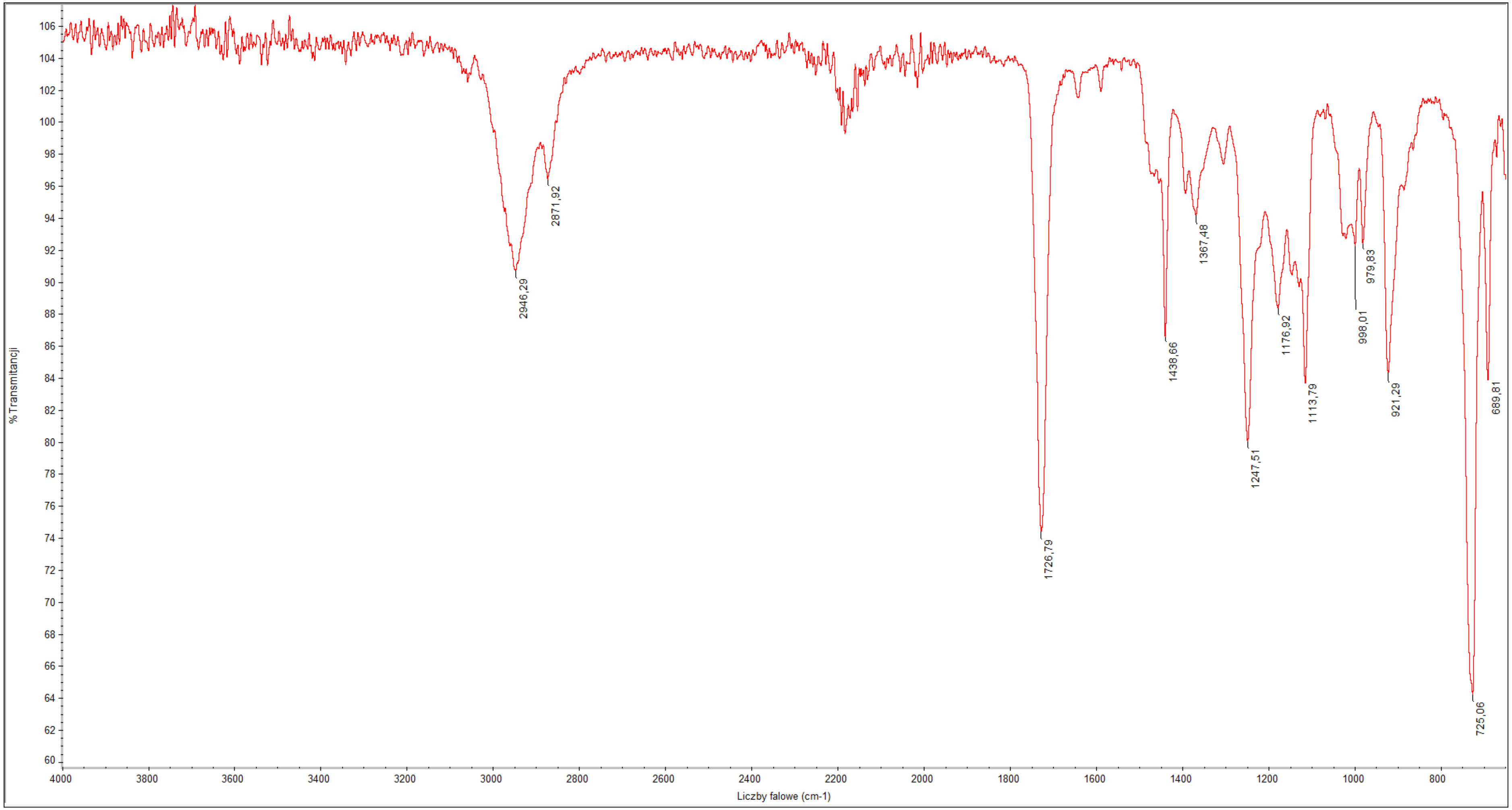

IR spectrum of product **7d**; ATR (cm^{-1}).

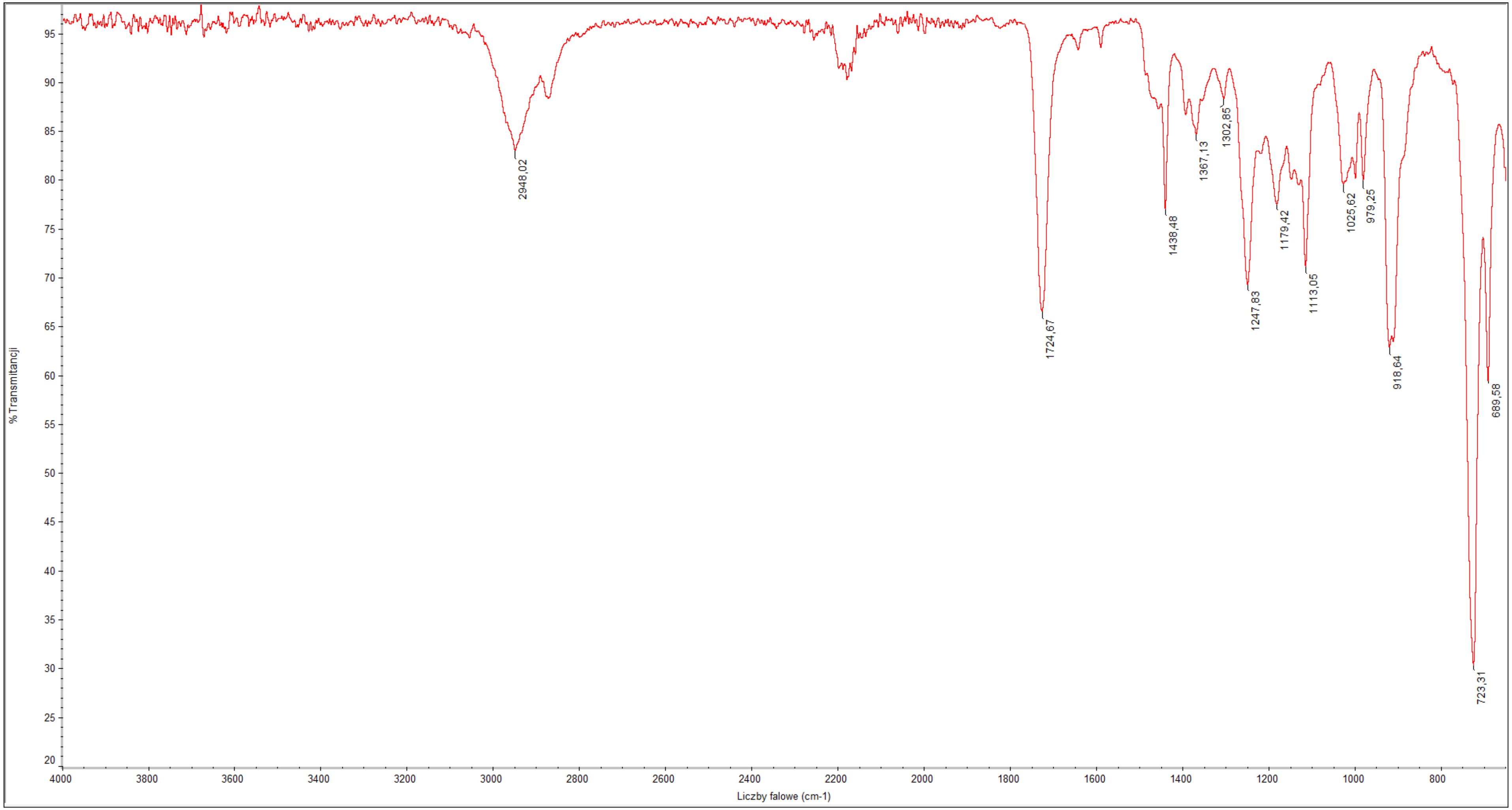

IR spectrum of product **7e**; ATR (cm^{-1}).

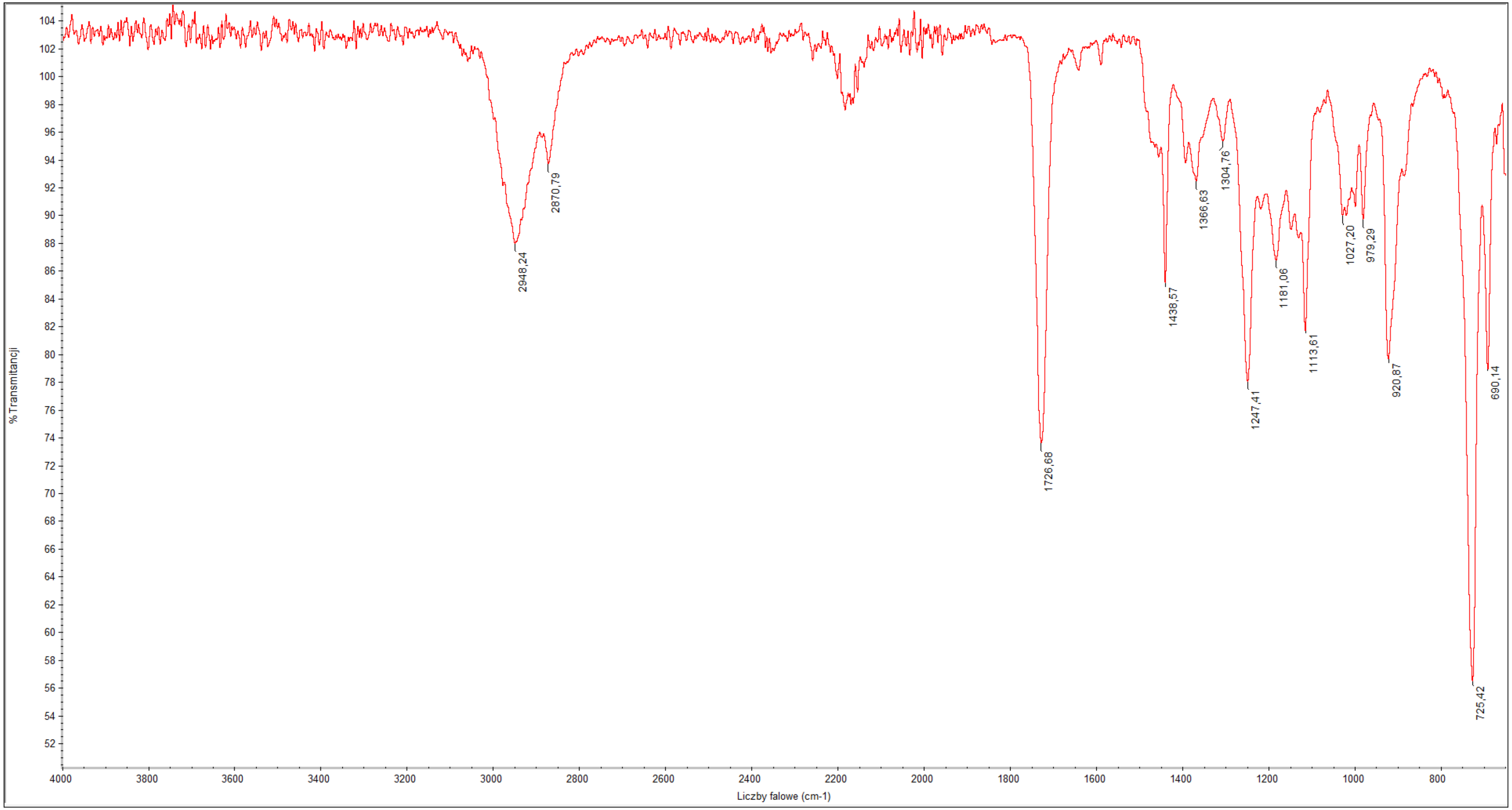

IR spectrum of product **7f**; ATR (cm^{-1}).

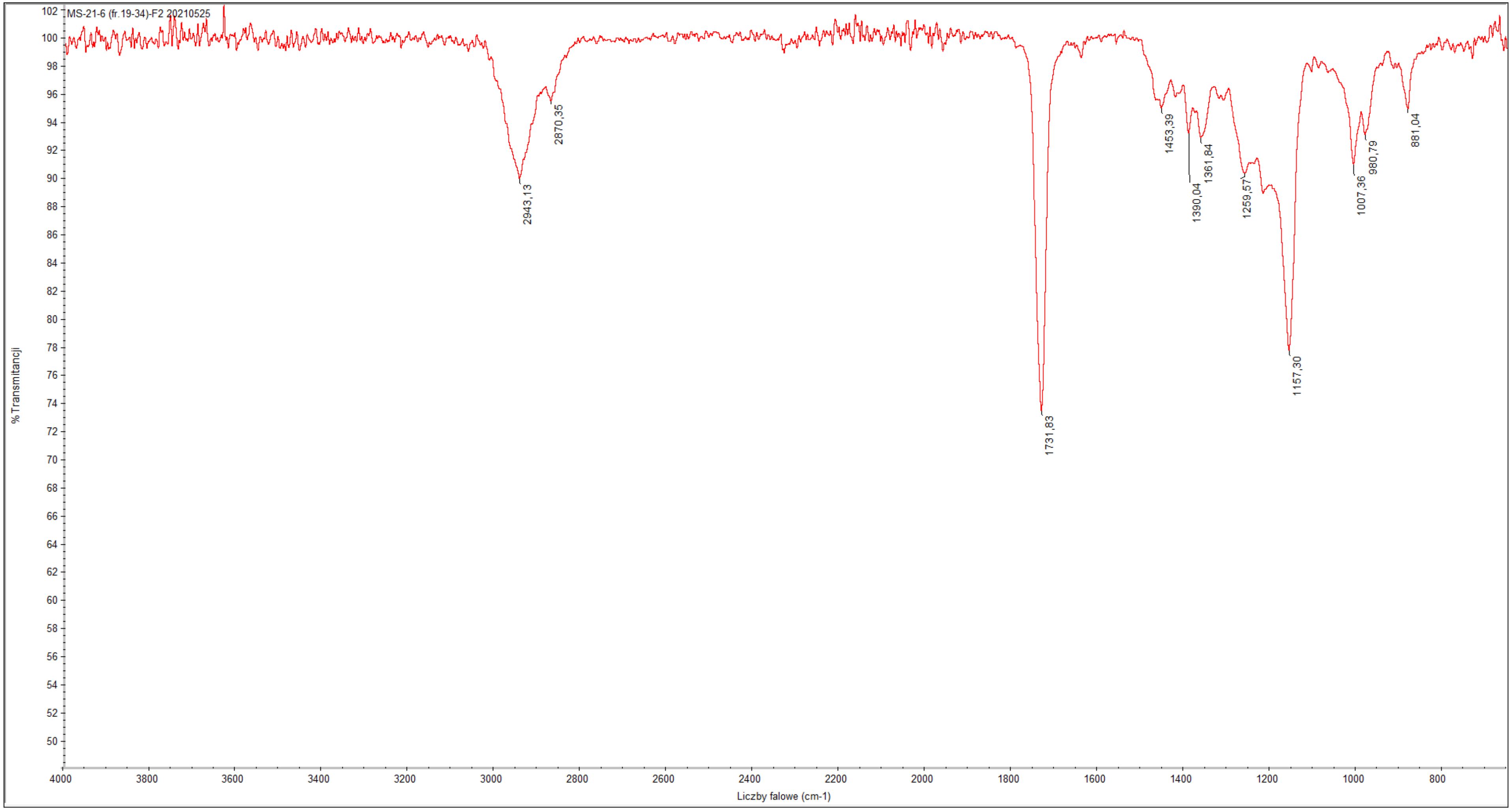

IR spectrum of product **8a**; ATR (cm^{-1}).

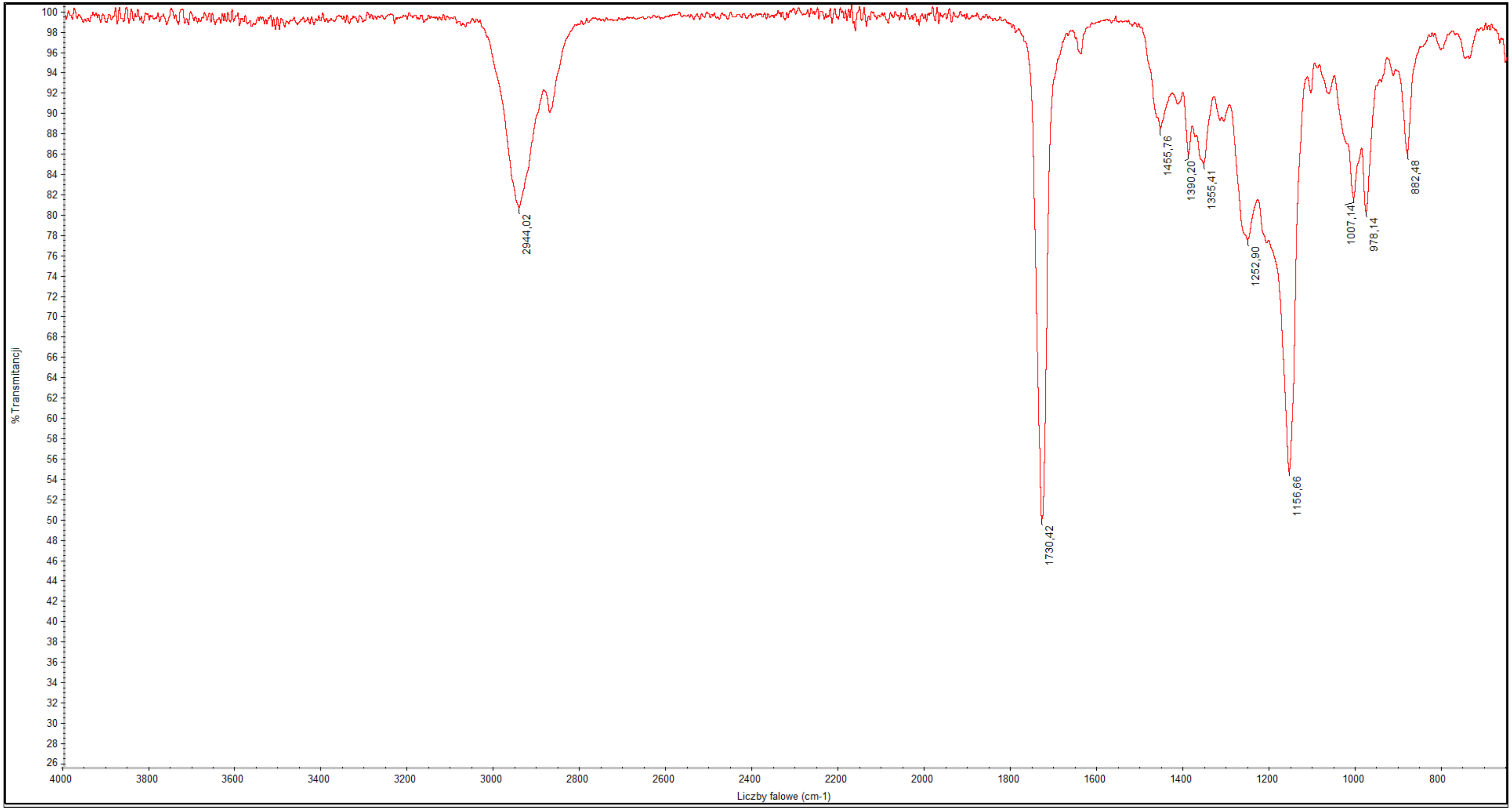

IR spectrum of product **8b**; ATR (cm^{-1}).

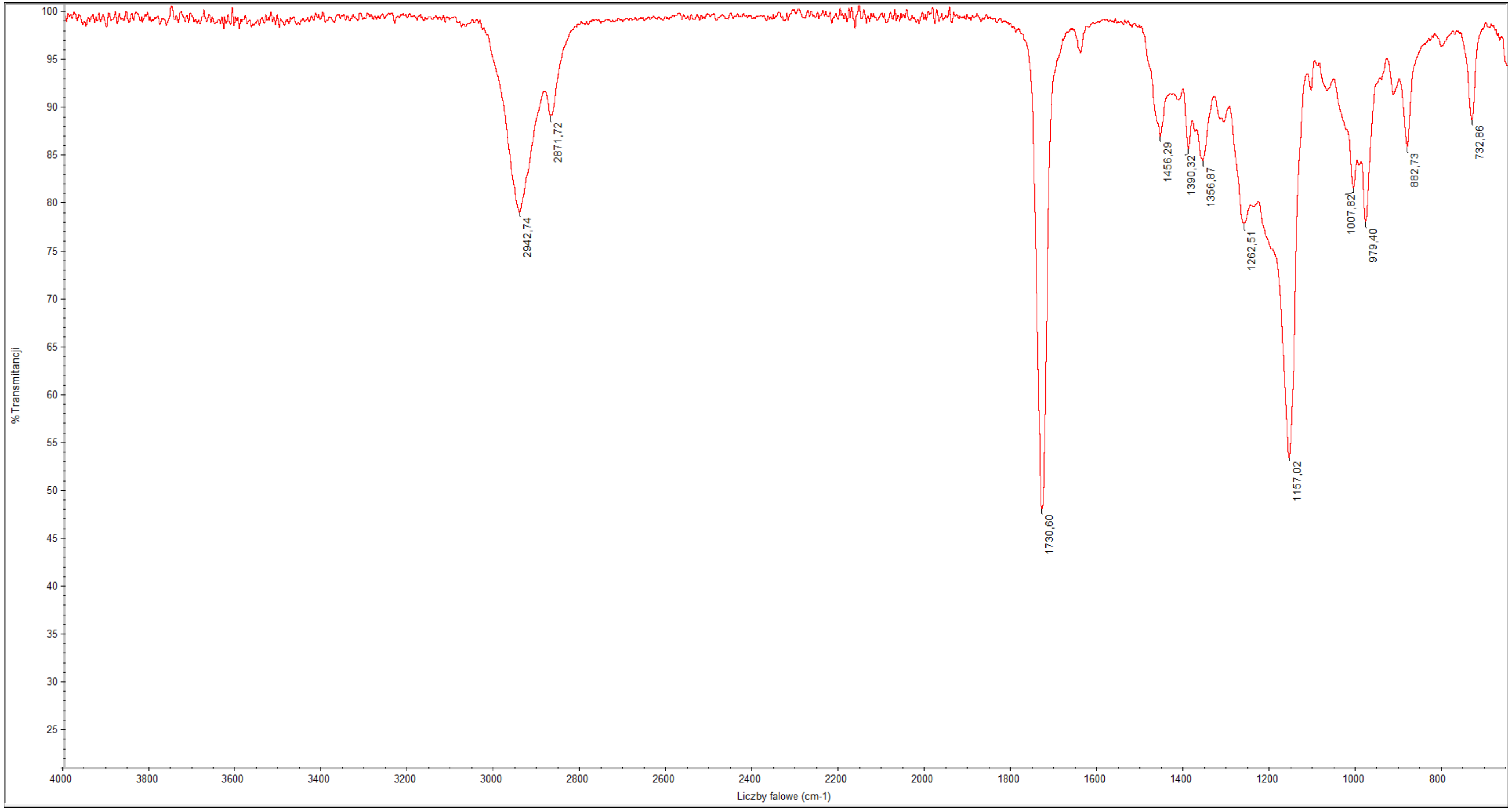

IR spectrum of product **8c**; ATR (cm^{-1}).

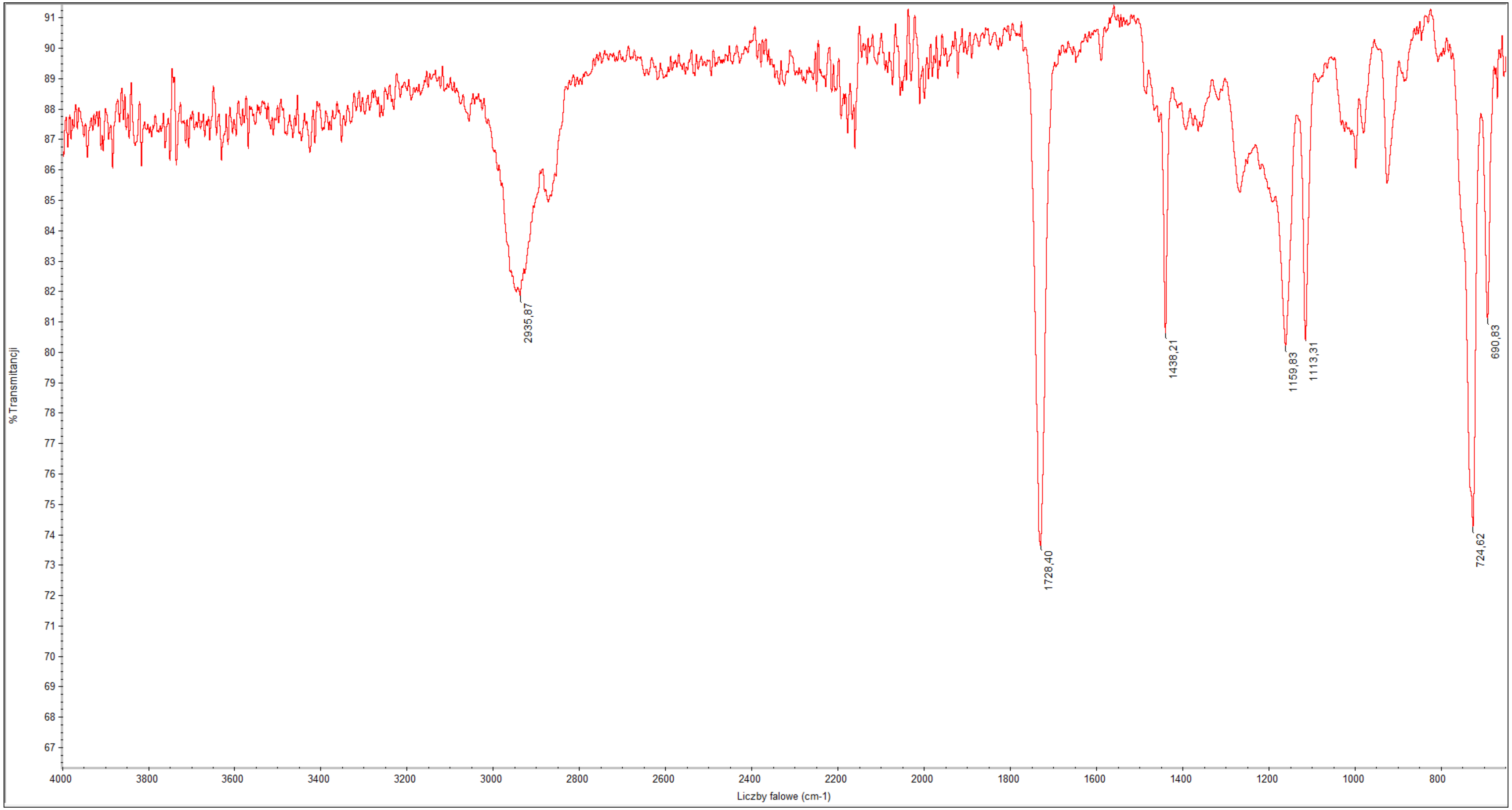

IR spectrum of product **9b**; ATR (cm^{-1}).

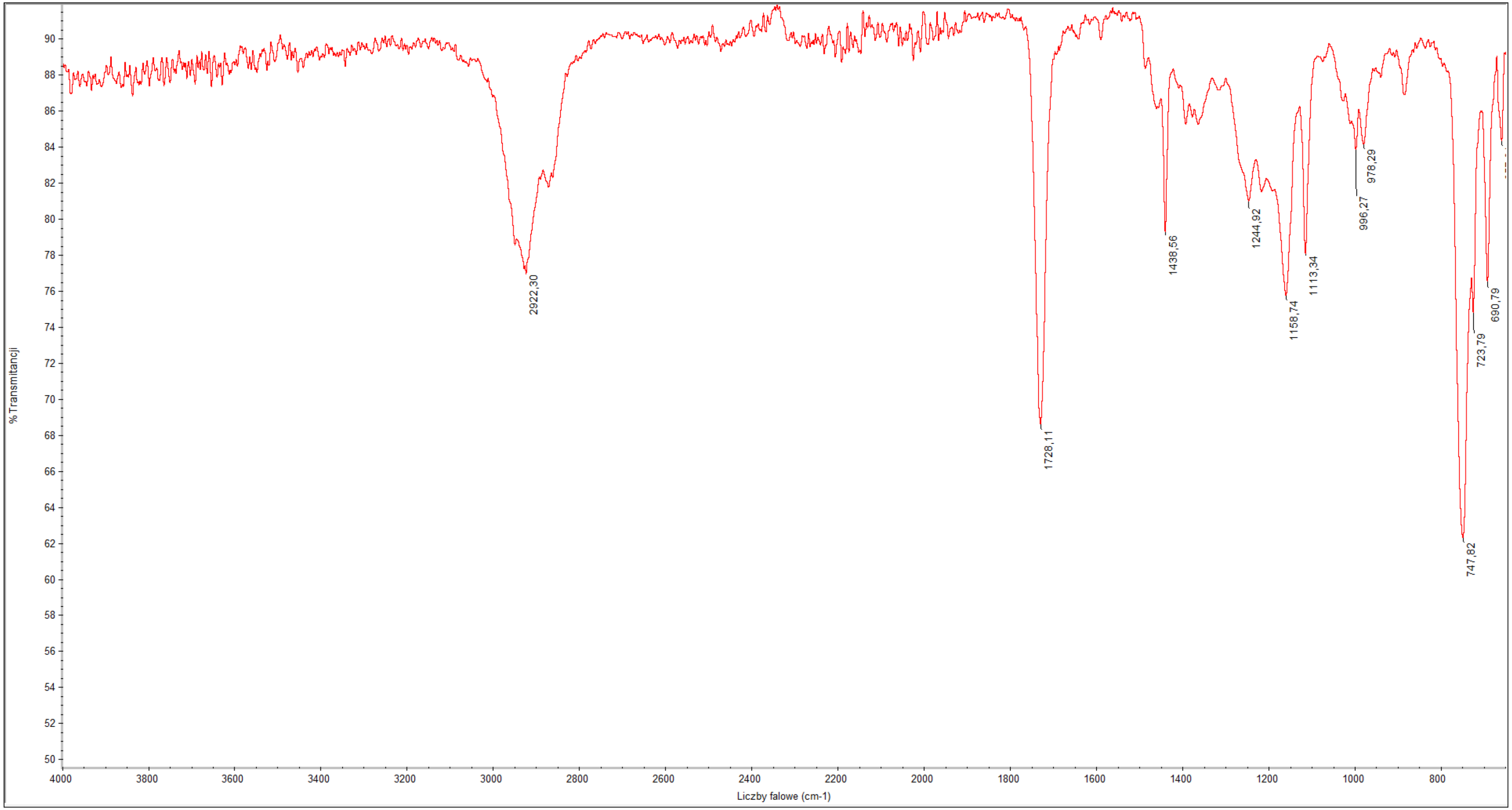

IR spectrum of product **9c**; ATR (cm^{-1}).
